# Diastereoselective Cascade Cyclization of Diazoimides with Alkylidene Pyrazolones for Preparation of Pyrazole-Fused Oxa-Bridged Oxazocines

**DOI:** 10.3390/molecules28207021

**Published:** 2023-10-10

**Authors:** Kuo Wang, Yue Zhang, Lu-Yu Cai, Xiu-Qing Song, Chen Wang, Jia-Rui Zhang, Yi-Fan Pan, Hong-Wu Zhao

**Affiliations:** College of Life Science and Bio-Engineering, Beijing University of Technology, No.100 Pingleyuan, Chaoyang District, Beijing 100124, China; wk1632748255@bjut.edu.cn (K.W.); zhangyueyue@emails.bjut.edu.cn (Y.Z.); cai@emails.bjut.edu.cn (L.-Y.C.); song@bjut.edu.cn (X.-Q.S.); chenw7132@emails.bjut.edu.cn (C.W.); zhangjiarui@emails.bjut.edu.cn (J.-R.Z.); panyifan@emails.bjut.edu.cn (Y.-F.P.)

**Keywords:** diastereoselectivity, cascade cyclization, diazoimide, alkylidenepyrazolone, oxazocine

## Abstract

Under the catalysis of Rh_2_(OAc)_4_ (10 mol%) and binapbisphosphine ligand (±)-**L3** (20 mol%) in DCE at 80 °C, the cascade cyclization of diazoimides with alkylidenepyrazolones underwent stereoselectively (dr > 20:1), affording pyrazole-fused oxa-bridged oxazocines in reasonable chemical yields. The chemical structure and relative configuration of title products were firmly identified by X-ray diffraction analysis.

## 1. Introduction

**Scheme 1:**
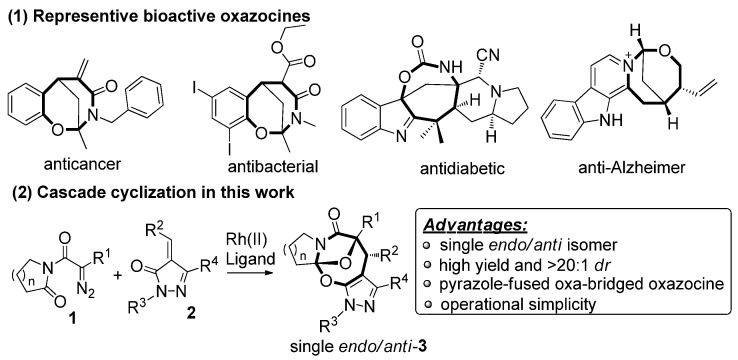
Representative bioactive oxazocines and the cascade cyclization in this work.

Oxazocines belong to a class of medicinally and biologically important chemical skeletons and have a wide range of biological activities such as anticancer, antibacterial, antidiabetic, and anti-Alzheimer properties [1,2] (Scheme 1(1)). Due to the variety of the significant bioactive potentials with oxazocine scaffolds, organic and medicinal chemists have created a variety of synthetic methodologies for their efficient and concise constructions [3,4,5,6,7,8]. Moreover, in this context, non-stereoselective and stereoselective cycloadditions have functioned as the crucial protocols for preparing a variety of structurally diverse and complex bridged and non-bridged oxazocines [9,10,11,12,13,14]. Importantly, elegant and powerful synthetic methodologies have appeared to prepare heterocycle-fused bridged oxazocines, but this area has witnessed slow development in recent years [15]. So, designing and exploring new synthetic protocols is urgently needed to obtain structurally new and unique oxa-bridged oxazocines fused with heterocyclic motifs.

Diazoimides constitute a kind of robust and versatile building block and they are broadly applied to prepare bioactive natural products and drug scaffolds by means of their cycloadditions [16,17,18,19,20,21,22,23]. Normally, when treated with transition metals, diazoimides can deliver structurally diverse acyclic or cyclic carbonyl ylides, enabling many types of cycloadditions with a wide range of dipolarophiles [24,25,26,27,28,29,30]. Primarily, the cycloaddition of cyclic carbonyl ylides derived from diazoimides with structurally various dipolarophiles can be used to more efficiently and concisely deliver oxa-bridged heterocyclic scaffolds [31,32,33,34,35]. On the basis of the previous advances concerning the cyclization of carbonyl yildes [36,37,38,39,40], we envisioned the transition-metal catalyzed novel cascade cyclization of diazoimides **1** with alkylidenepyrazolones **2**, which found a few examples of the cycloadditions centering on their enone moieties [41,42,43,44] (Scheme 1(2)). Pleasingly, in the presence of Rh_2_(OAc)_4_ (10 mol%) and binapbisphosphine ligand (±)-**L3** (20 mol%) in DCE at 80 °C, the newly designed cascade cyclization proceeded readily and diastereoselectively furnished pyrazole-fused oxa-bridged products **3** in reasonable chemical yields with excellent diastereoselectivities (>20:1 dr). Presumably, the formation of the aromatic pyrazole ring in the products substantially facilitates the cascade cyclization explored in our work. To the best of our knowledge, such a work has not been reported in the literature to date.

## 2. Results and Discussion

Initially, in benzene at 80 °C we scrutinized several transition-metal catalysts for their effects on the cascade cyclization of diazoimide **1a** with alkylidenepyrazolone **2a**, as described in Table 1. Generally, all the tested transition-metal catalysts provided product **3aa** with excellent diastereoselectivity (>20:1 dr). By comparison, the chemical yield of **3aa** highly depended on the screened transition-metal catalysts. In the presence of Ru(bpy)_3_Cl_2_.6H_2_O, the cascade cyclization delivered product **3aa** in a trace amount (entry 1). As for catalysts Ru(OAc)_3_ and Rh_2_(Oct)_4_, in contrast with Ru(bpy)_3_Cl_2_.6H_2_O, they behaved well to provide product **3aa** in 43% chemical yield (entries 1 vs. 2 and 3). In the case of Pd_2_(dba)_3_, Pd(PPh_3_)_2_Cl_2_, and Rh(OAc)_3_ as catalysts, they differently increased the chemical yield of the cascade cyclization (entries 1–3 vs. 4–6). Finally, we observed that catalyst Rh_2_(OAc)_4_ acted most efficiently to furnish product **3aa** in the highest chemical yield (entries 1–6 vs. 7).

Next, together with Rh_2_(OAc)_4_ as the catalyst in benzene at 80 °C, we examined a few mono- and bisphosphine ligands to clarify their effects on the cascade cyclization of diazoimide **1a** with alkylidenepyrazolone **2a**, as depicted in Table 2. In the absence of a ligand, the cascade cyclization generated product **3aa** in 82% chemical yield with >20:1 dr (entry 1). Regarding ligands dppf, (±)-**L1**, (±)-**L4**, and (±)-**L6**, they hugely inhibited the occurrence of the cascade cyclization and furnished product **3aa** in a trace amount after 10 h (entries 3–4, 7, and 9). Regarding ligands dppb, (±)-**L2**, and (±)-**L5**, they performed well to deliver product **3aa** in reasonable chemical yields with excellent diastereoselectivities (entries 2, 5, and 8). Lastly, we identified that ligand (±)-**L3** behaved quite efficiently to afford product **3aa** in a chemical yield of 88% (entry 6).

Moreover, with the use of Rh_2_(OAc)_4_ and (±)-**L3** at 80 °C, we examined a variety of differently polar solvents for their effects on the cascade cyclization of diazoimide **1a** with alkylidenepyrazolone **2a**, as outlined in Table 3. Generally, in the screened solvents, the cascade cyclization formed product **3aa** with excellent diastereoselectivity (>20:1 dr) (entries 1–8). However, the attempted solvents largely influenced the chemical yield of the cascade cyclization. Choosing DMF as the reaction solvent completely inhibited the occurrence of the cascade cyclization (entry 6). As for solvents HFIP and DCM, they afforded product **3aa** in a trace amount after 10 h (entries 7 and 9). Using 1,4-dioxane and THF as the reaction solvents, cascade cyclization generated product **3aa** in lower chemical yields (entries 2–3). Satisfyingly, we observed that in the aromatic solvents such as benzene, toluene, and PhCF_3_, the [4+3] cycloaddition produced product **3aa** in reasonable chemical yields (entries 1, 4–5). In the end, we found the cascade cyclization in solvent 1,2-DCE performed very well in terms of reactivity, chemical yield, and diastereoselectivity (entry 8). So, at this point, we determined the optimal reaction conditions for the cascade cyclization, as shown below: **1a**/**2a** = 1.5:1.0 (mmol/mmol), Rh_2_(OAc)_4_ (10 mol%), (±)-**L3** (20 mol%), DCE, 80 °C.

Finally, under optimal reaction conditions, we broadened the reaction scope of the cascade cyclization via diversifying substrates **1** and **2**, as summarized in Table 4. Basically, most of the explored cascade cyclizations behaved quite well in stereocontrols, and provided products **3** with excellent diastereoselectivities (>20:1 dr). By comparison, the chemical yield of the screened [4+3] cycloadditions ranged widely with the used substrates **1** and **2**. In the cascade cyclization with diazoimide **1a**, the substrates **2** with Me as the R^4^ group allowed for the introduction of electron-poor or electron-rich aryls at R^2^ or R^3^ positions, and delivered products **3aa**–**3ag** in 65–98% chemical yields (entries 1–7). Generally, in this context, the **2** substrates bearing an electron-rich aryl as the R^2^ or R^3^ group acted more efficiently than the **2** substrates possessing an electron-poor aryl as the R^2^ or R^3^ group, and gave rise to **3** products in higher chemical yields (e.g., entries 2–3 vs. 4; 6 vs. 7). Concerning the cascade cyclization with diazoimide **1a**, alkylidenepyrazolone **2h** (where R^2^ = R^3^ = R^4^ = Ph) resulted in the formation of **3ah** in 88% chemical yield (entry 8). Unfortunately, the substrates **2i** (R^2^ = Ph, R^3^ = Me, R^4^ = Ph), **2j** (R^2^ = Ph, R^3^ = Me, R^4^ = Me), and **2k** (R^2^ = Ph, R^3^ = Me, R^4^ = Ph) completely failed to conduct the [4+3] cycloaddition with diazoimide **1a** (entries 9–11). So, in this series, positioning an aryl group at the R^2^ position is indispensable to maintain the reactivity of the cascade cyclizations (entries 1 vs. 9; 8 vs. 9).

As for the cascade cyclization with alkylidenepyrazolone **2a**, substrate **1** tolerated the variation in the R^1^ group and the ring size of the lactam motif, and provided products **3ba**–**3ea** in 70–82% chemical yields (entries 12–15). Basically, in this series, the **1** substrates possessing a bigger ring-sized lactam subunit commonly worked better than the **1** substrates incorporating a smaller ring-sized lactam subunit with respect to the chemical yield of the cascade cyclization (entries 13 vs. 14 vs. 15). As for substrate **1f** containing a N-methylacetamide motif, it performed poorly in the cascade cyclization with substrate **2a** and produced product **3fa** in a lower chemical yield (entry 16). Even worse, substrate **1g** bearing a N-methylbenzamide subunit failed to conduct the cascade cyclization with substrate **2a** (entry 17). Therefore, in this context, the **1** substrates containing a lactam motif generally have higher reactivities than the **1** substrates bearing a N-methylamide moiety (entries 12–15 vs. 16–17).

**Scheme 2:**
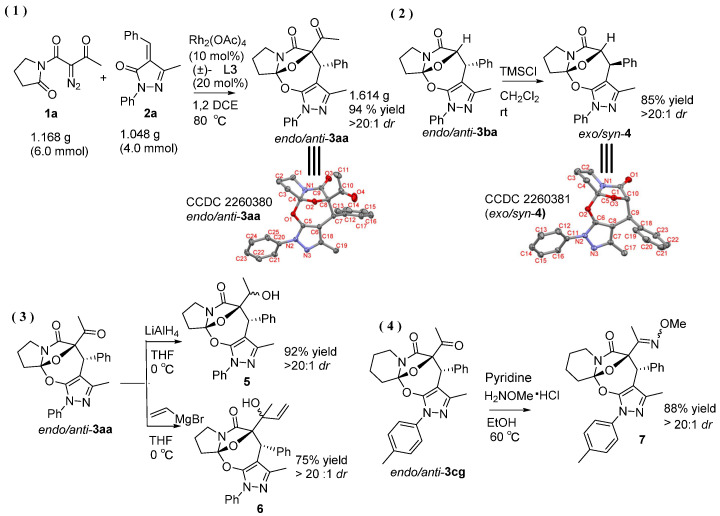
Gram-scale synthesis of **3aa** and chemical transformations of **3ba**, **3aa**, and **3cg**.

Moreover, with an aim of enriching the structural diversity and complexity of **3** products, we accomplished the crossed cascade cyclizations between substrates **1b**–**1e** with a broad spectrum of **2** substrates, as presented in entries 18–41 (Table 4). Apparently, in this regard, all the conducted cascade cyclizations showed excellent diastereoselectivities (>20:1 dr). In the case of the chemical yields of the cascade cyclizations, they highly depended on the selected **1** and **2** substrates and ranged widely from 50% to 95%. Mostly, the crossed cascade cyclizations behaved well, and thus delivered **3** products in moderate to high chemical yields (entries 18–39 and 41). Only the cascade cyclization between **1e** and **2n** behaved poorly, and provided product **3ej** in a quite low chemical yield (entry 40). Moreover, the oxindole-based enone **2q** reacted well with diazoimide **1b**, thus affording cycloadduct **3bl** in excellent chemical yield and diastereoselectivity (entry 41). Meanwhile, we firmly identified the chemical structure and relative stereo-configuration of the desired product **3aa** using single crystal X-ray analysis (CCDC 2260380) [45]; see details in SI) and assigned those of other title compounds by inference. In order to show the synthetic potentials of title **3** compounds, we prepared **3aa** at gram scale and completed several chemical transformations of title compounds **3ba**, **3aa,** and **3cg**, as described in Scheme 2. In the scale-up cascade cyclization, we isolated product **3aa** at a chemical yield of 94% (Scheme 2(1)). Upon treatment with TMSCl in CH_2_Cl_2_ at rt, compound **3ba** easily converted into its exo/syn stereoisomer **4** (CCDC 2260381) [45]; see details in Supplementary Materials) at a chemical yield of 85% (Scheme 2(2)). Furthermore, the reduction reaction of **3aa** with LiAlH_4_ in anhydrous THF at 0 °C gave **5** at a chemical yield of 92%; and the addition reaction of **3aa** with vinylmagnesium bromide in anhydrous THF at 0 °C produced **6** at a chemical yield of 75% (Scheme 2(3)). Lastly, in the presence of pyridine in EtOH at 60 °C, the condensation reaction between **3cg** and H_2_NOMe·HCl furnished **7** at a chemical yield of 88% (Scheme 2(4)).

**Scheme 3:**
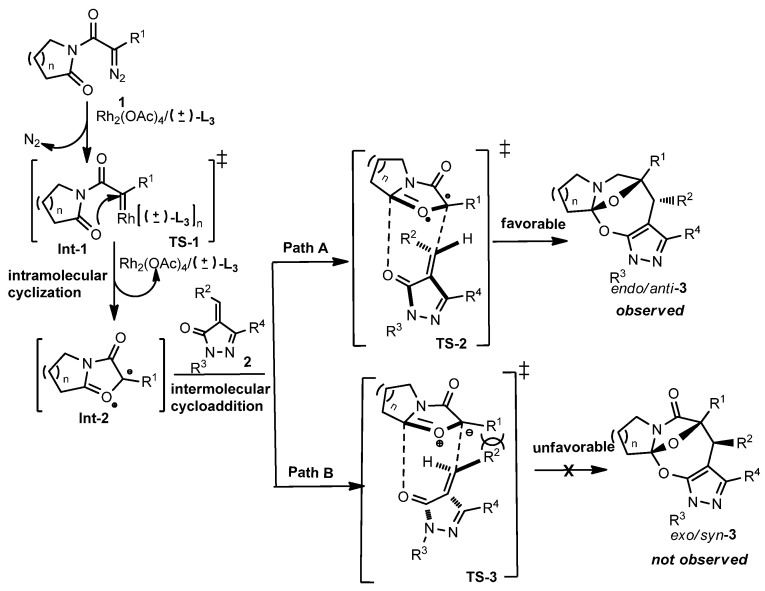
Proposed mechanism for formation of product **3**.

For the sake of clarifying the diastereoselective formation of product **3**, we proposed the reaction mechanism for the cascade cyclization between diazoimide **1** and alkylidenepyrazolone **2**, as illustrated in Scheme 3. Under the catalysis of Rh_2_(OAc)_4_ and (±)-**L3**, by liberating one molecule of N_2_, diazoimide **1** decomposes into Rh(II)-carbenoid **Int-1**. Then, **Int-1** transforms into cyclic carbonyl ylide **Int-2** through transition state **TS-1**. Concerning **Int-2**, which was formed in situ, it can conduct the cascade cyclization with alkylidenepyrazolone **2** by adopting transition states **TS-2** or **TS-3**. In **TS-3**, there exists strong steric repulsion between R^1^ and R^2^ motifs; in contrast, **TS-2** fully avoids this kind of unfavorable interaction. So, **TS-2** is thermodynamically more stable than **TS-3** and mainly accounts for the formation of single endo/anti-**3**.

## 3. Materials and Methods

Proton (^1^H), carbon (^13^C), and fluorine (^19^F) NMR spectra were recorded on a Bruker Avance HD III spectrometer (400 MHz for ^1^H NMR, 100 MHz for ^13^C NMR, and 376 MHz for ^19^F NMR) and calibrated using tetramethylsilane (TMS) as internal reference. High resolution mass spectra (HRMS) were obtained on an Agilent Technologies LC/MSD TOF spectrometer under electrospray ionization (ESI) conditions. The melting point of compounds was determined using a melting point instrument. Flash column chromatography was performed on silica gel (0.035–0.070 mm) using compressed air. X-ray single crystals were obtained on a Rigaku 002 Saturn 944 spectrometer. Thin layer chromatography (TLC) was carried out on 0.25 mm SDS silica gel coated glass plates (60F254). Eluted plates were visualized using a 254 nm UV lamp. Unless otherwise indicated, all reagents were commercially available and used without further purification. All solvents were distilled from the appropriate drying agents immediately before use. Diazoimides **1a**–**1g** were synthesized according to the reported procedures [10,46,47]. Alkylidenepyrazolones **2a**–**2q** were prepared according to literature procedures [48,49,50].

### 3.1. General Procedure for Cascade Cycloaddition Reaction

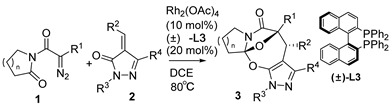



A mixture of diazoimides **1** (1.5 equiv, 0.15 mmol), alkylidenepyrazolones **2** (1.0 equiv, 0.1 mmol), Rh_2_(OAc)_4_ (10.0 mol%), and (±)-**L3** (20.0 mol%) in DCE (1.5 mL) was stirred at 80 °C. After the reaction was completed as indicated by the TLC plate, the solvent was concentrated under reduced pressure and the resulting crude products were purified by flash column chromatography on silica gel (petroleum ether/ethyl acetate = 6:1~2:1) to afford **3** products (35–98% yields).

### 3.2. Gram-Scale Synthesis of Compound ***3aa***

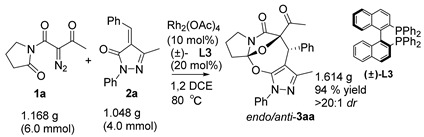



A mixture of diazoimide **1a** (1.5 equiv, 6.0 mmol, 1.168 g), alkylidenepyrazolone **2a** (1.0 equiv, 4.0 mmol, 1.048 g), Rh_2_(OAc)_4_ (10.0 mol%, 0.176 g), and (±)-**L3** (20.0 mol%, 0.497 g) in DCE (15 mL) was stirred at 80 °C. After the reaction was completed as indicated by the TLC plate, the solvent was concentrated under reduced pressure and the resulting crude product was purified by flash column chromatography on silica gel (petroleum ether/ethyl acetate = 4:1) to afford product **3aa** as a white solid (1.614 g, 94% yield).

### 3.3. Chemical Transformations of ***3ba***, ***3aa***, and ***3cg***

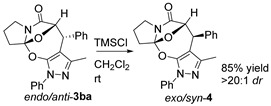



To a well-stirred solution of **3ba** (1.0 equiv, 0.1 mmol, 38.7 mg) in CH_2_Cl_2_ (1.0 mL), TMSCl (1.0 equiv, 0.1 mmol, 10.8 mg) was added. Then, the reaction mixture was stirred at room temperature for 12 h. After the reaction was finished as indicated by the TLC plate, the reaction mixture was concentrated under reduced pressure. The resulting reaction residue was purified using flash column chromatography on silica gel (petroleum ether/ethyl acetate = 2:1) to afford product **4** as a white solid (32.8 mg, 85% yield). M.P. = 75.3–75.8 °C; ^1^H NMR (400 MHz, CDCl_3_): *δ* 7.81 (d, *J* = 7.8 Hz, 2H), 7.44 (t, *J* = 7.6 Hz, 2H), 7.35–7.24 (m, 4H), 7.13 (d, *J* = 6.3 Hz, 2H), 5.69 (d, *J* = 1.8 Hz, 1H), 4.43 (d, *J* = 1.7 Hz, 1H), 3.96–3.75 (m, 2H), 2.87–2.62 (m, 2H), 2.30–2.16 (m, 2H), 2.10 (s, 3H) ppm;^13^C NMR (100 MHz, CDCl_3_): *δ*171.5, 148.3, 146.6, 140.2, 138.7, 128.9, 128.8, 128.0, 127.4, 126.4, 122.7, 122.5, 121.6, 100.4, 89.1, 45.7, 41.4, 34.4, 25.0, 12.9 ppm; HRMS (ESI-TOF) *m*/*z*: [M + H]^+^ Calcd for C_23_H_21_N_3_O_3_ 388.1656; Found 388.1655.

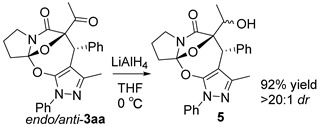



To a well-stirred solution of **3aa** (1.0 equiv, 0.1 mmol, 42.9 mg) in dry THF (1.0 mL), LiAlH_4_ (2.0 equiv, 0.2 mmol, 7.6 mg) was added. Then, the reaction mixture was stirred at 0 °C for 1 h. After the reaction was finished as indicated by the TLC plate, the reaction mixture was concentrated under reduced pressure. The resulting reaction residue was purified using flash column chromatography on silica gel (petroleum ether/ethyl acetate = 1:1) to afford product **5** as a white solid (39.7 mg, 92% yield). M.P. = 215.7–216.1 °C; ^1^H NMR (400 MHz, CDCl_3_): *δ*7.64 (d, *J* = 7.8 Hz, 2H), 7.47 (d, *J* = 7.1 Hz, 1H), 7.44 (t, *J* = 7.7 Hz, 2H), 7.36–7.25 (m, 4H), 7.19 (d, *J* = 4.8 Hz, 1H), 5.15 (s, 1H), 4.03–3.91 (m, 1H), 3.77–3.67 (m, 1H), 3.41–3.29 (m, 1H), 2.60–2.47 (m, 1H), 2.41–2.25 (m, 1H), 2.25–2.12 (m, 3H) 1.51 (s, 3H), 1.13 (d, *J* = 6.3 Hz, 3H) ppm;^13^C NMR (100 MHz, CDCl_3_): *δ* 171.5, 149.1, 145.8, 138.8, 136.8, 130.6, 130.0, 128.8, 128.1, 128.0, 127.7, 126.2, 122.6, 119.1, 100.7, 94.3, 66.3, 47.3, 42.4, 35.0, 24.9, 17.9, 14.7, 14.2 ppm; HRMS (ESI-TOF) *m*/*z*: [M + H]^+^ Calcd for C_25_H_25_N_3_O_4_ 432.1917; Found 432.1872.

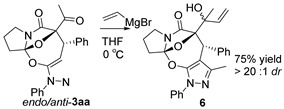



To a well-stirred solution of **3aa** (1.0 equiv, 0.1 mmol, 42.9 mg) in anhydrous THF (1.0 mL), 1.0 M vinylmagnesium bromide (2.0 equiv, 0.2 mmol, 0.2 mL) was added under N_2_. Then, the reaction mixture was stirred at 0 °C for 1 h. After the reaction was finished as indicated by the TLC plate, saturated NH_4_Cl solution was added to quench the reaction at 0 °C. Then, the reaction mixture was extracted with ethyl acetate, and the combined organic layers were dried over anhydrous Na_2_SO_4_. After filtration, the organic layers were concentrated under reduced pressure to give crude product. Finally, the crude product was purified using flash column chromatography on silica gel (petroleum ether/ethyl acetate = 2:1) to afford product **6** as a white solid (34.3 mg, 75% yield). M.P. = 92.7–93.1 °C; ^1^H NMR (400 MHz, CDCl_3_): δ 7.64 (dd, *J* = 8.7, 1.2 Hz, 2H), 7.44 (t, *J* = 7.6 Hz, 2H), 7.28 (td, *J* = 7.4,1.1 Hz, 3H), 7.26–7.12 (br, 3H), 6.06 (q, *J* = 10.8 Hz, 1H), 5.28 (dd, *J* = 17.3, 1.1 Hz, 1H), 5.14 (dd, *J* = 10.8, 1.2 Hz, 1H), 5.06 (s, 1H), 3.82–3.68 (m, 1H), 3.42 (s, 1H), 3.39–3.30 (m, 1H), 2.59–2.45 (m, 1H), 2.37–2.13 (m, 3H), 1.58 (s, 3H) 1.53 (s, 3H) ppm;^13^C NMR (100 MHz, CDCl_3_): δ173.4, 149.0, 145.1, 141.1, 138.8, 138.4, 128.8, 127.2, 126.2, 122.6, 119.4, 115.2, 101.7, 92.3, 48.2, 42.4, 35.1, 25.0, 22.5, 14.3 ppm; HRMS (ESI-TOF) *m*/*z*: [M + H]^+^ Calcd for C_27_H_27_N_3_O_4_ 458.2074; Found 458.2082.

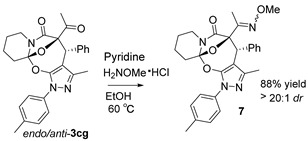



To a well-stirred solution of **3cg** (1.0 equiv, 0.1 mmol, 45.7 mg) in dry EtOH (1.0 mL), H_2_NOMe·HCl (2.0 equiv, 0.2 mmol, 16.6 mg) and pyridine (2.0 equiv, 0.2 mmol, 15.8 mg) were added. Then, the reaction mixture was stirred at 60 °C for 2 h. After the reaction was finished as indicated by the TLC plate, the reaction mixture was concentrated under reduced pressure. The resulting reaction residue was purified using flash column chromatography on silica gel (petroleum ether/ethyl acetate = 6:1) to afford product **7** as a white solid (42.7 mg, 88% yield). M.P. = 195.4–196.0 °C; ^1^H NMR (400 MHz, CDCl_3_): δ 7.55 (d, *J* = 8.2 Hz, 2H), 7.41 (d, *J* = 7.2 Hz, 2H), 7.29–7.17 (m, 5H), 4.70 (s, 1H), 3.91 (s, 3H), 3.86 (dd, *J* = 13.4, 4.4 Hz, 1H), 2.71 (d, *J* = 12.5 Hz, 1H), 2.52 (td, *J* = 12.8, 2.6 Hz, 1H), 2.39 (s, 3H), 2.04 (s, 3H), 1.96 (d, *J* = 12.7 Hz, 1H), 1.86 (td, *J* = 13.0, 4.0 Hz, 1H), 1.81–1.70 (m, 3H), 1.67 (s, 3H), 1.52–1.41 (m, 3H) ppm;^13^C NMR (100 MHz, CDCl_3_): δ168.6, 151.3, 147.9, 145.0, 138.4, 136.2, 136.0, 130.4, 129.4, 127.8, 127.0, 122.3, 110.5, 102.4, 87.3, 62.1, 47.5, 38.7, 34.2, 23.3, 21.0, 20.9, 12.9, 11.1 ppm; HRMS (ESI-TOF) *m*/*z*: [M + H]^+^ Calcd for C_28_H_30_N_4_O_4_ 487.2340; Found 487.2350.

### 3.4. Charaterization of 3 Products

*5-acetyl-3-methyl-1,4-diphenyl-4,5,9,10-tetrahydro-8H-5,10a-epoxypyrazolo[4,3-g]pyrrolo[2,1-b][1,3]oxazocin-6(1H)-one* (**3aa**)*:* White solid (yield: 42.0 mg, 98%). M.P. = 152.9–153.1 °C; ^1^H NMR (400 MHz, CDCl_3_): δ 7.64 (d, *J* = 7.7 Hz, 2H), 7.54–7.48 (br, 1H), 7.45 (t, *J* = 7.6 Hz, 2H), 7.30 (t, *J* = 7.4 Hz, 3H), 7.28–7.25 (br, 1H)), 7.20–7.14 (br, 1H), 5.12 (s, 1H), 3.92–3.81 (m, 1H), 3.50–3.38 (m, 1H), 2.68–2.56 (m, 1H), 2.47–2.24 (m, 3H), 2.16 (s, 3H), 1.56 (s, 3H) ppm; ^13^C NMR (100 MHz, CDCl_3_): δ 198.3, 169.4, 148.9, 145.3, 138.6, 135.8, 128.8, 128.0, 127.7, 126.3, 122.6, 119.8, 100.2, 93.4, 47.5, 42.8, 35.4, 26.7, 25.0, 14.3 ppm; HRMS (ESI-TOF) *m*/*z*: [M + H]^+^ Calcd for C_25_H_23_N_3_O_4_ 430.1761; Found 430.1759.
*5-acetyl-4-(4-bromophenyl)-3-methyl-1-phenyl-4,5,9,10-tetrahydro-8H-5,10a-epoxypyrazolo[4,3-g]pyrrolo[2,1-b][1,3]oxazocin-6(1H)-one* (**3ab**): White solid (yield: 37.0 mg, 73%). M.P. = 93.7–93.9 °C; ^1^H NMR (400 MHz, CDCl_3_): *δ* 7.63 (d, *J* = 8.7 Hz, 2H), 7.47 (s, 1H), 7.46 (s, 2H), 7.44 (s, 1H), 7.38 (d, *J* = 8.2 Hz, 1H), 7.32 (t, *J* = 7.4 Hz, 1H), 7.02 (d, *J* = 8.3 Hz, 1H), 5.12 (s, 1H), 3.93–3.84 (m, 1H), 3.47–3.38 (m, 1H), 2.67–2.56 (m, 1H), 2.46–2.25 (m, 3H), 2.59 (s, 3H), 1.60 (s, 3H) ppm; ^13^C NMR (100 MHz, CDCl_3_): δ 198.1, 169.3, 148.6, 145.3, 138.5, 135.2, 131.2, 131.1, 128.8, 126.4, 122.6, 121.8, 120.0, 99.7, 93.2, 46.4, 43.0, 35.5, 26.5, 25.1, 14.4 ppm; HRMS (ESI-TOF) *m*/*z*: [M + H]^+^ Calcd for C_25_H_22_BrN_3_O_4_ 508.0866; Found 508.0867.
*5-acetyl-3-methyl-4-(4-nitrophenyl)-1-phenyl-4,5,9,10-tetrahydro-8H-5,10a-epoxypyrazolo[4,3-g]pyrrolo[2,1-b][1,3]oxazocin-6(1H)-one* (**3ac**): White solid (yield: 30.8 mg, 65%). M.P. = 109.9–110.3 °C; ^1^H NMR (400 MHz, CDCl_3_): *δ* 8.20 (d, *J* = 7.6 Hz, 1H), 8.09 (d, *J* = 8.1 Hz, 1H), 7.83 (d, *J* = 7.9 Hz, 1H), 7.63 (dd, *J* = 8.7, 1.2 Hz, 2H), 7.47 (t, *J* = 7.5 Hz, 2H), 7.36–7.27 (m, 2H), 5.30 (s, 1H), 3.92–3.86 (m, 1H), 3.49–3.42 (m, 1H), 2.67–2.62 (m, 1H), 2.48–2.34 (m, 3H), 2.21 (s, 3H), 1.58 (s, 3H) ppm; ^13^C NMR (100 MHz, CDCl_3_): *δ* 197.9, 169.1, 148.2, 147.2, 145.4, 144.2, 138.4, 132.7, 130.6, 129.0, 126.7, 123.1, 122.7, 120.5, 99.2, 93.2, 46.3, 43.1, 35.5, 26.2, 25.2, 14.3ppm; HRMS (ESI-TOF) *m*/*z*: [M + H]^+^ Calcd for C_25_H_22_N_4_O_6_ 475.1612; Found 475.1631.
*5-acetyl-4-(4-methoxyphenyl)-3-methyl-1-phenyl-4,5,9,10-tetrahydro-8H-5,10a-epoxypyrazolo[4,3-g]pyrrolo[2,1-b][1,3]oxazocin-6(1H)-one* (**3ad**): White solid (yield:37.6 mg, 82%). M.P. = 87.7–88.1 °C; ^1^H NMR (400 MHz, CDCl_3_): *δ*7.63 (dd, *J* = 8.7, 1.2 Hz, 2H), 7.45 (t, *J* = 7.5 Hz, 2H), 7.40 (d, *J* = 7.6 Hz, 1H), 7.30 (t, *J* = 7.7 Hz, 1H), 7.07 (d, *J* = 8.0 Hz, 1H), 6.82 (t, *J* = 4.4 Hz 2H), 5.06 (s, 1H), 3.90–3.83 (m, 1H), 3.80 (s, 3H), 3.45–3.39 (m, 1H), 2.65–2.58 (m, 1H), 2.42–2.31 (m, 3H), 2.16 (s, 3H), 1.58 (s, 3H) ppm; ^13^C NMR (100 MHz, CDCl_3_): *δ* 198.6, 169.5, 159.0, 149.0, 145.2, 138.7, 132.5, 130.7, 128.9, 127.6, 126.3, 122.6, 119.6, 113.7, 113.1, 100.4, 93.4, 55.1, 46.9, 42.8, 35.5, 26.8, 25.0, 14.4 ppm; HRMS (ESI-TOF) *m*/*z*: [M + H]^+^ Calcd for C_26_H_25_N_3_O_5_ 460.1867; Found 460.1864.
*5-acetyl-3-methyl-4-(naphthalen-2-yl)-1-phenyl-4,5,9,10-tetrahydro-8H-5,10a-epoxypyrazolo[4,3-g]pyrrolo[2,1-b][1,3]oxazocin-6(1H)-one* (**3ae**): White solid (yield: 35.9 mg, 75%). M.P. = 168.5–168.8 °C; ^1^H NMR (400 MHz, CDCl_3_): *δ* 7.89–7.68 (m, 3H), 7.51–7.47 (m, 3H), 7.48 (t, *J* = 7.4 Hz, 4H), 7.33 (t, *J* = 7.4 Hz, 2H), 5.36 (s, 1H), 3.90–3.85 (m, 1H), 3.49–3.42 (m, 1H), 2.66–2.59 (m, 1H), 2.48–2.24 (m, 3H), 2.16 (s, 3H), 1.53 (s, 3H) ppm; ^13^C NMR (100 MHz, CDCl_3_): *δ* 198.6, 198.3, 169.5, 148.9, 145.4, 138.7, 132.8, 128.9, 128.1, 127.7, 127.6, 126.4, 126.1, 126.0, 122.6, 119.8, 99.8, 93.2, 47.6, 42.9, 35.5, 26.8, 25.0, 14.4 ppm; HRMS (ESI-TOF) *m*/*z*: [M + H]^+^ Calcd for C_29_H_25_N_3_O_4_ 480.1917; Found 480.1922.
*5-acetyl-1-(4-chlorophenyl)-3-methyl-4-phenyl-4,5,9,10-tetrahydro-8H-5,10a-epoxypyrazolo[4,3-g]pyrrolo[2,1-b][1,3]oxazocin-6(1H)-one* (**3af**): White solid (yield: 33.3 mg, 72%). M.P. = 143.2–143.5 °C; ^1^H NMR (400 MHz, CDCl_3_): *δ* 7.60 (dd, *J* = 8.8, 2.0 Hz, 2H), 7.51–7.47 (br, 1H), 7.41 (dt, *J* = 8.9, 2.7 Hz, 2H), 7.34–7.22 (br, 3H), 7.18–7.08 (br, 1H), 5.12 (s, 1H), 3.91–3.81 (m, 1H), 3.46–3.37 (m, 1H), 2.64–2.56 (m, 1H), 2.42–2.56 (m, 3H), 2.16 (s, 3H), 1.55 (s, 3H) ppm; ^13^C NMR (100 MHz, CDCl_3_): *δ* 198.2, 169.3, 149.3, 145.4, 137.2, 135.6, 131.7, 131.5, 128.9, 128.0, 127.8, 123.6, 119.8, 100.4, 93.4, 47.4, 42.9, 35.5, 26.7, 25.0, 14.3 ppm; HRMS (ESI-TOF) *m*/*z*: [M + H]^+^ Calcd for C_25_H_22_ClN_3_O_4_ 464.1371; Found 464.1369.
*5-acetyl-3-methyl-4-phenyl-1-(p-tolyl)-4,5,9,10-tetrahydro-8H-5,10a-epoxypyrazolo[4,3-g]pyrrolo[2,1-b][1,3]oxazocin-6(1H)-one* (**3ag**): White solid (yield: 35.4 mg, 80%). M.P. = 149.2–149.6 °C; ^1^H NMR (400 MHz, CDCl_3_): *δ* 7.50 (d, *J* = 8.4 Hz, 3H), 7.36–7.26 (br, 2H), 7.26 (t, *J* = 7.8 Hz, 3H), 7.21–7.12 (br, 1H), 5.12 (s, 1H), 3.90–3.82 (m, 1H), 3.45–3.37 (m, 1H), 2.65–2.55 (m, 1H), 2.41 (s, 3H), 2.40–2.25 (m, 3H), 2.15 (s, 3H), 1.55 (s, 3H) ppm; ^13^C NMR (100 MHz, CDCl_3_): *δ* 198.5, 169.4, 148.6, 145.3, 136.2, 135.9, 131.5, 129.7, 129.4, 128.6, 127.7, 122.7, 120.0, 100.0, 93.5, 47.6, 42.8, 35.5, 26.8, 25.0, 21.1, 14.3 ppm; HRMS (ESI-TOF) *m*/*z*: [M + H]^+^ Calcd for C_26_H_25_N_3_O_4_ 444.1917; Found 444.1920.
*5-acetyl-1,3,4-triphenyl-4,5,9,10-tetrahydro-8H-5,10a-epoxypyrazolo[4,3-g]pyrrolo[2,1-b][1,3]oxazocin-6(1H)-one* (**3ah**): White solid (yield: 42.2 mg, 86%). M.P. = 76.2–76.6 °C; ^1^H NMR (400 MHz, CDCl_3_): *δ* 7.75 (d, *J* = 5.7 Hz, 2H), 7.49 (t, *J* = 8.3 Hz, 2H), 7.34 (t, *J* = 7.4 Hz, 1H), 7.14–7.01 (m, 7H), 6.99 (d, *J* = 1.8 Hz, 3H), 5.39 (s, 1H), 3.90–3.82 (m, 1H), 3.48–3.39 (m, 1H), 2.67–2.55 (m, 1H), 2.46–2.25 (m, 3H), 2.14 (s, 3H) ppm; ^13^C NMR (100 MHz, CDCl_3_): *δ*198.5, 169.4, 152.0, 145.7, 138.7, 135.6, 133.7, 128.9, 128.5, 127.5, 127.4, 127.2, 126.7, 123.0, 119.9, 100.7, 93.9, 47.4, 42.9, 35.5, 26.8, 25.0 ppm; HRMS (ESI-TOF) *m*/*z*: [M + H]^+^ Calcd for C_30_H_25_N_3_O_4_ 492.1918; Found 492.1932.
*3-methyl-1,4-diphenyl-4,5,9,10-tetrahydro-8H-5,10a-epoxypyrazolo[4,3-g]pyrrolo[2,1-b][1,3]oxazocin-6(1H)-one* (**3ba**): White solid (yield: 29.8 mg, 77%). M.P. = 146.8–147.0 °C; ^1^H NMR (400 MHz, CDCl_3_): *δ*7.64 (d, *J* = 7.9 Hz, 2H), 7.46 (t, *J* = 7.6 Hz, 2H), 7.40–7.32 (m, 4H), 7.30–7.26 (m, 2H), 4.78 (dd, *J* = 13.6, 6.6 Hz, 2H), 3.78–3.71 (m, 1H), 3.35–3.29 (m, 1H), 2.55–2.51 (m, 1H), 3.34–2.21 (m, 3H), 1.61 (s, 3H) ppm; ^13^C NMR (100 MHz, CDCl_3_): *δ* 171.0, 148.6, 146.5, 138.7, 135.0, 129.5, 129.5, 128.9, 128.8, 128.8, 128.5, 128.3, 128.0, 126.4, 122.7, 122.5, 120.8, 99.8, 86.1, 47.8, 42.2, 35.1, 24.8, 14.9 ppm; HRMS (ESI-TOF) *m*/*z*: [M + H]^+^ Calcd for C_23_H_21_N_3_O_3_ 388.1656; Found 388.1622.
*5-acetyl-3-methyl-1,4-diphenyl-4,5,8,9,10,11-hexahydro-5,11a-epoxypyrazolo[4,3-g]pyrido[2,1-b][1,3]oxazocin-6(1H)-one* (**3ca**): White solid (yield: 31.0 mg, 70%). M.P. = 213.7–213.9 °C; ^1^H NMR (400 MHz, CDCl_3_): δ 7.67 (d, *J* = 7.6 Hz, 2H), 7.16 (t, *J* = 7.6 Hz, 2H), 7.35–7.21 (m, 6H), 4.80 (s, 1H), 3.89 (dd, *J* = 13.3, 4.6 Hz, 1H), 2.76 (d, *J* = 12.8 Hz, 1H), 2.55 (td, *J* = 12.9, 3.0 Hz, 1H), 2.00–1.95 (br, 1H), 1.99 (s, 3H), 1.93 (s, 3H), 1.90–1.83 (m, 1H), 1.82–1.70 (m, 2H), 1.53–1.39 (m, 1H) ppm; ^13^C NMR (100 MHz, CDCl_3_): *δ* 200.5, 165.5, 148.3, 145.1, 138.3, 136.7, 129.7, 128.9, 128.4, 127.7, 126.6, 122.4, 110.4, 101.2, 91.9, 48.0, 38.9, 34.4, 27.4, 23.3, 20.8, 12.9 ppm; HRMS (ESI-TOF) *m*/*z*: [M + H]^+^ Calcd for C_26_H_25_N_3_O_4_ 444.1918; Found 444.1872.
*5-acetyl-3-methyl-1,4-diphenyl-4,5,9,10,11,12-hexahydro-8H-5,12a-epoxyazepino[2,1-b]pyrazolo[4,3-g][1,3]oxazocin-6(1H)-one* (**3da**): White solid (yield: 34.2 mg, 75%). M.P. = 195.0–195.3 °C; ^1^H NMR (400 MHz, CDCl_3_): *δ* 7.64 (d, *J* = 7.7 Hz, 2H), 7.45 (t, *J* = 7.6 Hz, 2H), 7.34–7.21 (m, 6H), 4.79 (s, 1H), 3.85 (d, *J* = 13.9 Hz, 1H), 2.90–2.79 (m, 2H), 2.40–2.31 (m, 1H), 1.98 (s, 3H), 1.96 (s, 3H) 1.94–1.85 (m, 2H), 1.78–1.69 (br, 1H), 1.53–1.33 (m, 3H) ppm; ^13^C NMR (100 MHz, CDCl_3_): *δ* 200.6, 166.4, 148.1, 145.3, 138.4, 136.9, 129.6, 128.9, 128.4, 127.7, 126.5, 122.5, 115.4, 100.8, 91.6, 48.0, 40.5, 38.4, 29.5, 28.5, 27.6, 22.5, 12.9 ppm; HRMS (ESI-TOF) *m*/*z*: [M + H]^+^ Calcd for C_27_H_27_N_3_O_4_ 458.2074; Found 458.2039.
*5-acetyl-3-methyl-1,4-diphenyl-4,5,8,9,10,11,12,13-octahydro-5,13a-epoxyazocino[2,1-b]pyrazolo[4,3-g][1,3]oxazocin-6(1H)-one* (**3ea**): White solid (yield: 38.6 mg, 82%). M.P. = 149.5–149.8 °C; ^1^H NMR (400 MHz, CDCl_3_): *δ* 7.64 (d, *J* = 7.7 Hz, 2H), 7.45 (t, *J* = 7.6 Hz, 2H), 7.34–7.21 (m, 6H), 4.80 (s, 1H), 3.70 (dt, *J* = 14.3, 4.0 Hz, 1H), 2.80 (td, *J* = 11.9, 3.6 Hz, 1H), 2.66 (dt, *J* = 16.1, 4.4 Hz, 1H), 2.50 (td, *J* = 10.8, 4.4 Hz, 1H), 1.98 (s, 3H), 1.96 (s, 3H), 1.86–1.75 (m, 3H), 1.70–1.55 (m, 3H), 1.53–1.35 (m, 2H) ppm; ^13^C NMR (100 MHz, CDCl_3_): *δ* 200.6, 167.0, 148.2, 145.2, 138.5, 136.8, 129.6, 128.9, 128.4, 127.7, 126.4, 122.3, 114.1, 100.9, 91.4, 48.4, 40.4, 31.7, 27.6, 27.0, 25.0, 23.8, 22.1, 12.9 ppm; HRMS (ESI-TOF) *m*/*z*: [M + H]^+^ Calcd for C_28_H_29_N_3_O_4_ 472.2230; Found 472.2236.
*5-acetyl-3,7,8-trimethyl-1,4-diphenyl-4,5,7,8-tetrahydro-5,8-epoxypyrazolo[4,3-g][1,3]oxazocin-6(1H)-one* (**3fa**): White solid (yield: 14.6 mg, 35%). M.P. = 192.5–192.9 °C; ^1^H NMR (400 MHz, CDCl_3_): *δ* 7.64 (dd, *J* = 8.7, 1.2 Hz, 2H), 7.45 (t, *J* = 7.6 Hz, 2H), 7.38–7.25 (m, 6H), 4.80 (s, 1H), 2.76 (s, 3H), 2.09 (s, 3H), 1.98 (s, 3H), 1.93 (s, 3H), ppm; ^13^C NMR (100 MHz, CDCl_3_): *δ* 200.2, 167.3, 148.2, 145.1, 138.3, 136.7, 129.7, 129.0, 128.4, 127.7, 126.6, 122.4, 112.5, 101.0, 91.8, 48.1, 27.3, 25.9, 23.0, 12.9 ppm; HRMS (ESI-TOF) *m*/*z*: [M + H]^+^ Calcd for C_24_H_23_N_3_O_4_ 418.1761; Found 418.1767.
*4-(4-bromophenyl)-3-methyl-1-phenyl-4,5,9,10-tetrahydro-8H-5,10a-epoxypyrazolo[4,3-g]pyrrolo[2,1-b][1,3]oxazocin-6(1H)-one* (**3bb**): White solid (yield: 41.8 mg, 90%). M.P. = 106.6–106.9 °C; ^1^H NMR (400 MHz, CDCl_3_): *δ* 7.63 (d, *J* = 7.8 Hz, 2H), 7.53 (dd, *J* = 8.1, 1.8 Hz, 1H), 7.50–7.41 (m, 3H), 7.31 (d, *J* = 7.4 Hz, 1H), 7.25 (dd, *J* = 8.0, 2.0 Hz, 1H), 7.15 (dd, *J* = 8.4, 2.1 Hz, 1H), 4.74 (dd, *J* = 14.9, 6.6 Hz, 2H), 3.78–3.69 (m, 1H), 3.35–3.27 (m, 1H), 2.57–2.45 (m, 1H), 2.36–2.21 (m, 3H), 1.63 (s, 3H) ppm; ^13^C NMR (100 MHz, CDCl_3_): *δ* 170.8, 148.3, 146.5, 138.6, 134.2, 131.7, 131.6, 131.3, 131.0, 128.9, 126.5, 122.7, 122.0, 120.9, 99.3, 85.7, 47.2, 42.2, 35.0, 26.9, 24.8, 15.0 ppm; HRMS (ESI-TOF) *m*/*z*: [M + H]^+^ Calcd for C_23_H_20_BrN_3_O_3_ 466.0760; Found 466.0769.
*3-methyl-4-(4-nitrophenyl)-1-phenyl-4,5,9,10-tetrahydro-8H-5,10a-epoxypyrazolo[4,3-g]pyrrolo[2,1-b][1,3]oxazocin-6(1H)-one* (**3bc**): White solid (yield: 34.5 mg, 80%). M.P. = 122.3–122.7 °C; ^1^H NMR (400 MHz, CDCl_3_): *δ* 8.28 (dd, *J* = 8.3, 2.2 Hz, 1H), 8.20 (dd, *J* = 8.3, 2.2 Hz, 1H), 7.63 (d, *J* = 8.0 Hz, 2H), 7.56 (dd, *J* = 8.4, 1.4 Hz, 1H), 7.46 (t, *J* = 7.4 Hz, 3H), 7.33 (t, *J* = 7.4 Hz, 1H), 4.93 (d, *J* = 6.6 Hz, 1H), 4.77 (d, *J* = 6.6 Hz, 1H), 3.80–3.69 (m, 1H), 3.38–3.27 (m, 1H), 2.57–2.45 (m, 1H), 2.36–2.21 (m, 3H), 1.62 (s, 3H) ppm; ^13^C NMR (100 MHz, CDCl_3_): *δ* 171.2, 159.2, 148.7, 146.4, 138.7, 130.6, 130.5, 129.0 128.9 128.8, 127.0, 126.3, 122.7, 122.4, 120.7, 114.1, 113.8, 113.8, 100.1, 86.2, 55.2, 47.0, 42.1, 35.0, 24.8, 14.9 ppm; HRMS (ESI-TOF) *m*/*z*: [M + H]^+^ Calcd for C_23_H_20_N_4_O_5_ 433.1506; Found 433.1510.
*3-methyl-1-phenyl-4-(3,4,5-trimethoxyphenyl)-4,5,9,10-tetrahydro-8H-5,10a-epoxypyrazolo[4,3-g]pyrrolo[2,1-b][1,3]oxazocin-6(1H)-one* (**3bl**): White solid (yield: 36.7 mg, 77%). M.P. = 89.7–90.0 °C; ^1^H NMR (400 MHz, CDCl_3_): *δ* 7.63 (d, *J* = 7.7 Hz, 2H), 7.44 (t, *J* = 7.6 Hz, 2H), 7.30 (d, *J* = 7.4 Hz, 1H), 6.54 (dd, *J* = 28.8, 1.6, Hz, 2H), 4.73 (d, *J* = 6.0 Hz, 1H), 4.65 (d, *J* = 6.0 Hz, 1H), 3.91 (s, 3H), 3.89 (s, 3H), 3.78 (s, 3H), 3.76–3.69 (m, 1H), 3.39–3.30 (m, 1H), 2.54–2.48 (m, 1H), 2.35–2.20 (m, 3H), 1.67 (s, 3H) ppm; ^13^C NMR (100 MHz, CDCl_3_): *δ* 171.2, 153.3, 153.0 148.6, 146.5, 138.7, 137.7, 130.6, 128.8, 126.4, 122.6, 122.4, 120.6, 107.1, 106.8, 100.0, 86.0 61.0, 56.4, 56.2, 48.3, 42.3, 35.0, 24.9, 14.8 ppm; HRMS (ESI-TOF) *m*/*z*: [M + H]^+^ Calcd for C_26_H_27_N_3_O_6_478.1972; Found 478.1980.
*3-methyl-1-phenyl-4-(4-(trifluoromethyl)phenyl)-4,5,9,10-tetrahydro-8H-5,10a-epoxypyrazolo[4,3-g]pyrrolo[2,1-b][1,3]oxazocin-6(1H)-one* (**3bm**): White solid (yield: 40.0 mg, 88%). M.P. = 168.7–168.9 °C; ^1^H NMR (400 MHz, CDCl_3_): *δ* 7.70 -7.56 (m, 4H),7.52–7.42 (m, 3H), 7.40 (d, *J* = 8.2 Hz, 1H), 7.32 (t, *J* = 7.4 Hz, 1H), 4.87 (d, *J* = 6.6 Hz, 1H), 4.76 (d, *J* = 6.6 Hz, 1H), 3.80–3.69 (m, 1H), 3.39–3.28 (m, 1H), 2.57–2.48 (m, 1H), 2.35–2.20 (m, 3H), 1.60 (s, 3H) ppm; ^13^C NMR (100 MHz, CDCl_3_): *δ* 170.6, 148.1, 146.6, 139.3, 138.6, 130.3, 130.0, 130.0, 129.7, 128.9, 126.6, 125.6, 125.3, 125.3, 122.8, 121.0, 99.0, 85.7, 47.6, 42.2, 35.0, 26.9, 24.8, 15.0 ppm; ^19^F NMR (376 MHz, CDCl_3_): *δ* 62.4 ppm; HRMS (ESI-TOF) *m*/*z*: [M + H]^+^ Calcd for C_24_H_20_F_3_N_3_O_3_ 456.1529; Found 456.1534.
*4-(2-methoxyphenyl)-3-methyl-1-phenyl-4,5,9,10-tetrahydro-8H-5,10a-epoxypyrazolo[4,3-g]pyrrolo[2,1-b][1,3]oxazocin-6(1H)-one* (**3bn**): White solid (yield: 38.7 mg, 93%). M.P. = 158.2–158.5 °C; ^1^H NMR (400 MHz, CDCl_3_): *δ*7.66 (d, *J* = 7.6 Hz, 2H), 7.45 (t, *J* = 7.6 Hz, 2H), 7.34–7.26 (m, 2H), 7.18 (dd, *J* = 7.6, 1.5 Hz, 1H), 6.95 (t, *J* = 7.6 Hz, 2H), 5.35 (d, *J* = 7.0 Hz, 1H), 4.91 (d, *J* = 7.0 Hz, 1H), 3.93 (s, 3H), 3.78–3.65 (m, 1H), 3.35–3.27 (m, 1H), 2.57–2.46 (m, 1H), 2.35–2.17 (m, 3H), 1.69 (s, 3H) ppm; ^13^C NMR (100 MHz, CDCl_3_): *δ* 171.5, 156.8, 148.6, 146.9, 138.9, 130.5, 129.5, 128.8, 128.5, 126.2, 123.1, 122.6, 122.4, 122.3, 121.6, 120.9, 120.6, 120.4, 110.1, 99.7, 87.0, 84.1, 55.4, 42.1, 39.4, 35.2, 24.8, 14.7 ppm; HRMS (ESI-TOF) *m*/*z*: [M + H]^+^ Calcd for C_24_H_23_N_3_O_4_ 418.1761; Found 418.1763.
*3,4,4-trimethyl-1-phenyl-4,5,9,10-tetrahydro-8H-5,10a-epoxypyrazolo[4,3-g]pyrrolo[2,1-b][1,3]oxazocin-6(1H)-one* (**3bo**): White solid (yield: 32.2 mg, 95%). M.P. = 94.4–94.9 °C; ^1^H NMR (400 MHz, CDCl_3_): *δ*7.54 (dd, *J* = 8.7, 1.2 Hz, 2H), 7.40 (t, *J* = 8.2 Hz, 2H), 7.27 (d, *J* = 7.2 Hz, 1H), 4.25 (s, 1H), 3.77–3.56 (m, 1H), 3.30–3.06 (m, 1H), 2.50–2.40 (m, 1H), 2.37 (s, 3H), 2.29–2.17 (m, 3H), 1.57 (s, 3H), 1.41 (s, 3H) ppm; ^13^C NMR (100 MHz, CDCl_3_): *δ* 171.7, 146.9, 144.7, 138.6, 128.7, 126.4, 123.0, 120.0, 105.9, 92.2, 41.5, 39.5, 34.4, 28.6, 24.9, 22.1, 16.5 ppm; HRMS (ESI-TOF) *m*/*z*: [M + H]^+^ Calcd for C_19_H_21_N_3_O_3_ 340.1656; Found 340.1660.
*5-acetyl-3-methyl-4-(4-nitrophenyl)-1-phenyl-4,5,8,9,10,11-hexahydro-5,11a-epoxypyrazolo[4,3-g]pyrido[2,1-b][1,3]oxazocin-6(1H)-one* (**3cc**): White solid (yield: 39.0 mg, 80%). M.P. = 231.6–231.9 °C); ^1^H NMR (400 MHz, CDCl_3_): *δ* 8.15 (d, *J* = 8.7 Hz, 2H), 7.66 (d, *J* = 8.0 Hz, 2H), 7.53 (d, *J* = 8.7 Hz, 2H), 7.47 (t, *J* = 7.7 Hz, 2H), 7.34 (t, *J* = 7.4 Hz, 1H), 4.95 (s, 1H), 3.91 (dd, *J* = 13.2, 4.5 Hz, 1H), 2.79 (d, *J* = 13.2 Hz, 1H), 2.56 (td, *J* = 13.0, 3.0 Hz, 1H), 2.03 (d, *J* = 13.5 Hz, 1H), 1.98 (s, 6H), 1.91 (dd, *J* = 13.3, 4.1 Hz, 1H), 1.86–1.68 (m, 2H), 1.56–1.42 (m, 1H) ppm; ^13^C NMR (100 MHz, CDCl_3_): *δ* 200.0, 164.9, 147.9, 147.4, 145.2, 144.5, 138.0, 130.8, 129.0, 126.9, 123.5, 122.5, 110.8, 100.2, 91.5, 46.8, 39.2, 34.4, 27.2, 23.2, 20.8, 12.9 ppm; HRMS (ESI-TOF) *m*/*z*: [M + H]^+^ Calcd for C_26_H_24_N_4_O_6_ 489.1768; Found 489.1773.
*5-acetyl-4-(4-methoxyphenyl)-3-methyl-1-phenyl-4,5,8,9,10,11-hexahydro-5,11a-epoxypyrazolo[4,3-g]pyrido[2,1-b][1,3]oxazocin-6(1H)-one* (**3cd**): White solid (yield: 42.5 mg, 90%). M.P. = 155.4–155.8 °C; ^1^H NMR (400 MHz, CDCl_3_): *δ* 7.66 (d, *J* = 7.6 Hz, 2H), 7.50 (t, *J* = 5.8 Hz, 2H), 7.30 (t, *J* = 7.4 Hz, 1H), 7.23 (d, *J* = 8.7 Hz, 2H), 6.80 (d, *J* = 8.7 Hz, 2H), 4.76 (s, 1H), 3.87 (dd, *J* = 13.3, 4.6 Hz, 1H), 3.77 (s, 3H), 2.75 (d, *J* = 13.2 Hz, 1H), 2.55 (td, *J* = 13.0, 3.0 Hz, 1H), 2.03–1.99 (m, 1H), 1.98 (s, 3H), 1.94 (s, 3H), 1.86 (dd, *J* = 13.3, 4.1 Hz, 1H), 1.82–1.67 (m, 2H), 1.52–1.41 (m, 1H) ppm; ^13^C NMR (100 MHz, CDCl_3_): *δ* 200.7, 165.5, 158.9, 148.3, 145.0, 138.3, 130.7, 128.9, 128.8, 126.5, 122.4, 113.7, 110.3, 101.4, 92.0, 55.2, 47.3, 38.9, 34.4, 27.5, 23.2, 20.8, 12.9 ppm; HRMS (ESI-TOF) *m*/*z*: [M + H]^+^ Calcd for C_27_H_27_N_3_O_5_ 474.2023; Found 474.2032.
*5-acetyl-3-methyl-4-(naphthalen-2-yl)-1-phenyl-4,5,8,9,10,11-hexahydro-5,11a-epoxypyrazolo[4,3-g]pyrido[2,1-b][1,3]oxazocin-6(1H)-one* (**3ce**): White solid. (yield: 46.8 mg, 95%). M.P. = 202.2–202.5 °C; ^1^H NMR (400 MHz, CDCl_3_): *δ* 7.87 -7.78 (m, 2H), 7.77 (t, *J* = 4.5 Hz, 2H), 7.71 (d, *J* = 7.6 Hz, 2H), 7.52–7.45 (m, 5H), 7.33 (t, *J* = 7.4 Hz, 1H), 5.00 (s, 1H), 3.93 (dd, *J* = 13.3, 4.6 Hz, 1H), 2.81 (d, *J* = 13.2 Hz, 1H), 2.60 (td, *J* = 13.0, 3.0 Hz, 1H), 2.06–2.01 (m, 1H), 2.00 (s, 3H), 1.92 (s, 3H), 1.89 (d, *J* = 3.8 Hz, 1H), 1.86–1.74 (m, 2H), 1.55–1.43 (m, 1H) ppm; ^13^C NMR (100 MHz, CDCl_3_): *δ* 200.5, 165.5, 148.4, 145.2, 138.3, 134.3, 133.0, 132.8, 129.0, 128.8, 128.2, 128.1, 127.6, 127.4, 126.6, 126.2, 126.1, 122.4, 110.5, 101.2, 92.1, 48.0, 39.0, 34.5, 27.5, 23.3, 20.9, 12.9 ppm; HRMS (ESI-TOF) *m*/*z*: [M + H]^+^ Calcd for C_30_H_27_N_3_O_4_ 494.2074; Found 494.2079.
*5-acetyl-1-(4-chlorophenyl)-3-methyl-4-phenyl-4,5,8,9,10,11-hexahydro-5,11a-epoxypyrazolo[4,3-g]pyrido[2,1-b][1,3]oxazocin-6(1H)-one* (**3cf**): White solid (yield: 38.1 mg, 80%). M.P. = 113.3–113.7 °C; ^1^H NMR (400 MHz, CDCl_3_): *δ* 7.64 (d, *J* = 8.8 Hz, 2H), 7.44 (d, *J* = 8.8 Hz, 2H), 7.33–7.22 (m, 5H), 4.80 (s, 1H), 3.89 (dd, *J* = 13.3, 4.6 Hz, 1H), 2.76 (d, *J* = 13.2 Hz, 1H), 2.52 (td, *J* = 13.0, 3.0 Hz, 1H), 2.08 (d, *J* = 13.4 Hz, 1H), 1.98 (s, 3H), 1.94 (s, 3H), 1.92–1.64 (m, 3H), 1.57–1.39 (m, 1H) ppm; ^13^C NMR (100 MHz, CDCl_3_): *δ* 200.3, 165.4, 148.7, 145.2, 136.9, 136.5, 132.0, 129.6, 129.1, 128.4, 127.7, 123.3, 110.6, 101.5, 91.8, 48.0, 39.0, 34.4, 27.4, 23.2, 20.9, 12.9 ppm; HRMS (ESI-TOF) *m*/*z*: [M + H]^+^ Calcd for C_26_H_24_ClN_3_O_4_ 478.1528; Found 478.1532.
*5-acetyl-3-methyl-4-phenyl-1-(p-tolyl)-4,5,8,9,10,11-hexahydro-5,11a-epoxypyrazolo[4,3-g]pyrido[2,1-b][1,3]oxazocin-6(1H)-one* (**3cg**): White solid (yield: 38.3 mg, 84%). M.P. = 144.7–148.1 °C; ^1^H NMR (400 MHz, CDCl_3_): *δ* 7.53 (d, *J* = 8.4 Hz, 2H), 7.31 (td, *J* = 8.5, 1.6 Hz, 2H), 7.29–7.22 (m, 5H), 4.80 (s, 1H), 3.89 (dd, *J* = 13.3, 4.6 Hz, 1H), 2.75 (d, *J* = 13.2 Hz, 1H), 2.52 (td, *J* = 13.0, 3.0 Hz, 1H), 2.41 (s, 3H), 2.04–1.99 (m, 1H), 1.98 (s, 3H), 1.94 (s, 3H), 1.92–1.68 (m, 3H), 1.53–1.39 (m, 1H) ppm; ^13^C NMR (100 MHz, CDCl_3_): *δ* 200.6, 165.5, 147.9, 145.0, 136.8, 136.4, 135.8, 129.7, 129.5, 128.3, 127.6, 122.4, 110.3, 101.0, 91.9, 48.0, 38.9, 34.4, 27.4, 23.3, 21.1, 20.8, 12.9 ppm; HRMS (ESI-TOF) *m*/*z*: [M + H]^+^ Calcd for C_27_H_27_N_3_O_4_ 458.2074; Found 458.2079.
*5-acetyl-3-methyl-1-phenyl-4-(3,4,5-trimethoxyphenyl)-4,5,8,9,10,11-hexahydro-5,11a-epoxypyrazolo[4,3-g]pyrido[2,1-b][1,3]oxazocin-6(1H)-one* (**3cl**): White solid (yield: 39.9 mg, 75%). M.P. = 238.3–238.7 °C; ^1^H NMR (400 MHz, CDCl_3_): *δ* 7.66 (d, *J* = 7.6 Hz, 2H), 7.46 (t, *J* = 7.6 Hz, 2H), 7.32 (t, *J* = 7.4 Hz, 1H), 6.54 (s, 2H), 4.74 (s, 1H), 3.89 (dd, *J* = 13.3, 4.6 Hz, 1H), 3.83 (s, 6H), 3.82 (s, 3H), 2.74 (d, *J* = 13.2 Hz, 1H), 2.57 (td, *J* = 13.0, 3.0 Hz, 1H), 2.04 (s, 3H), 1.98 (s, 3H), 1.90 (td, *J* = 13.1, 3.9 Hz, 1H), 1.85–1.66 (m, 1H),1.56–1.41 (m, 1H) ppm; ^13^C NMR (100 MHz, CDCl_3_): *δ* 200.6, 165.4, 152.8, 148.4, 145.0, 138.3, 137.5, 132.4, 129.0, 126.6, 122.4, 110.4, 106.9, 101.0, 91.9, 60.8, 56.2, 48.2, 39.0, 34.4, 27.6, 23.2, 20.8, 13.0 ppm; HRMS (ESI-TOF) *m*/*z*: [M + H]^+^ Calcd for C_29_H_31_N_3_O_7_534.2234; Found 534.2234.
*5-acetyl-4-(2-methoxyphenyl)-3-methyl-1-phenyl-4,5,8,9,10,11-hexahydro-5,11a-epoxypyrazolo[4,3-g]pyrido[2,1-b][1,3]oxazocin-6(1H)-one* (**3cn**): White solid (yield: 40.6 mg, 86%). M.P. = 162.3–162.5 °C; ^1^H NMR (400 MHz, CDCl_3_): *δ* 7.66 (d, *J* = 7.6 Hz, 2H), 7.45 (t, *J* = 5.8 Hz, 3H), 7.30 (t, *J* = 7.4 Hz, 1H), 7.21 (td, *J* = 8.5, 1.6 Hz, 1H), 6.88 (m, 2H), 5.51 (s, 1H), 3.92 (s, 3H), 3.89 (dd, *J* = 13.3, 4.6 Hz, 1H), 2.74 (d, *J* = 13.2 Hz, 1H), 2.56 (td, *J* = 13.0, 3.0 Hz, 1H), 2.20 (s, 3H), 2.00 (s, 3H), 1.96–1.87 (m, 2H), 1.81–1.68 (m, 2H), 1.52–1.37 (m, 1H) ppm; ^13^C NMR (100 MHz, CDCl_3_): *δ* 198.9, 166.2, 155.5, 148.4, 145.1, 138.4 130.9, 129.0, 128.9, 126.4, 125.3, 122.4, 121.0, 110.5, 110.2, 102.1, 92.0, 55.5, 39.1, 38.8, 34.4, 26.8, 23.2, 20.8, 12.7 ppm; HRMS (ESI-TOF) *m*/*z*: [M + H]^+^ Calcd for C_27_H_27_N_3_O_5_. 474.2023; Found 474.2026.
*5-acetyl-3-methyl-1-phenyl-4-(thiophen-2-yl)-4,5,8,9,10,11-hexahydro-5,11a-epoxypyrazolo[4,3-g]pyrido[2,1-b][1,3]oxazocin-6(1H)-one* (**3cp**): White solid (yield: 38.1 mg, 85%). M.P. = 181.6–181.9 °C; ^1^H NMR (400 MHz, CDCl_3_): *δ* 7.65 (d, *J* = 8.7 Hz, 2H), 7.45 (t, *J* = 7.6 Hz, 2H), 7.31 (t, *J* = 7.5 Hz, 1H), 7.19 (d, *J* = 5.0 Hz, 1H), 6.96 (d, *J* = 3.0 Hz, 1H), 6.90 (t, *J* = 14.0 Hz, 2H), 5.17 (s, 1H), 3.87 (dd, *J* = 13.3, 4.6 Hz, 1H), 2.76 (d, *J* = 13.2 Hz, 1H), 2.52 (td, *J* = 13.0, 3.0 Hz, 1H), 2.10 (s, 3H), 2.09 (s, 3H), 2.04–1.84 (m, 2H), 1.82–1.65 (m, 2H), 1.54–1.39 (m, 1H) ppm; ^13^C NMR (100 MHz, CDCl_3_): *δ* 200.3, 164.9, 148.2, 144.4, 139.9, 138.2, 128.9, 127.2, 126.7, 126.5, 125.7, 122.5, 110.5, 101.3, 91.7, 43.1, 39.0, 34.4, 27.3, 23.2, 20.8, 12.8 ppm; HRMS (ESI-TOF) *m*/*z*: [M + H]^+^ Calcd for C_24_H_23_N_3_O_4_S 450.1482; Found 450.1486.
*5-acetyl-4-(4-bromophenyl)-3-methyl-1-phenyl-4,5,9,10,11,12-hexahydro-8H-5,12a-epoxyazepino[2,1-b]pyrazolo[4,3-g][1,3]oxazocin-6(1H)-one* (**3db**): White solid (yield: 32.1 mg, 60%). M.P. = 212.0–212.2 °C; ^1^H NMR (400 MHz, CDCl_3_): *δ*7.63 (d, *J* = 7.5 Hz, 2H), 7.47 (t, *J* = 7.6 Hz, 2H), 7.40 (d, *J* = 8.4 Hz, 2H),7.32 (t, *J* = 7.4 Hz, 1H), 7.20 (d, *J* = 8.5 Hz, 2H), 4.77 (s, 1H), 3.86 (dd, *J* = 13.3, 4.6 Hz, 1H), 2.91–2.75 (m, 2H), 2.37–2.29 (m, 1H), 2.00 (s, 3H), 1.97 (s, 3H), 1.96–1.88 (m, 2H), 1.79–1.71 (m, 1H), 1.54–1.39 (m, 3H) ppm; ^13^C NMR (100 MHz, CDCl_3_): *δ* 200.5, 166.1, 148.0, 145.3, 138.3, 136.1, 131.5, 131.4, 129.0, 126.6, 122.5, 121.8, 115.6, 100.3, 91.4, 47.1, 40.6, 38.4, 29.5, 28.5, 27.7, 22.6, 12.9 ppm; HRMS (ESI-TOF) *m*/*z*: [M + H]^+^ Calcd for C_27_H_26_BrN3O_4_ 536.1179; Found 536.1189.
*5-acetyl-4-(4-methoxyphenyl)-3-methyl-1-phenyl-4,5,9,10,11,12-hexahydro-8H-5,12a-epoxyazepino[2,1-b]pyrazolo[4,3-g][1,3]oxazocin-6(1H)-one* (**3dd**): White solid (yield: 37.9 mg, 78%). M.P. = 161.0–161.4 °C; ^1^H NMR (400 MHz, CDCl_3_): *δ* 7.63 (d, *J* = 7.5 Hz, 2H), 7.45 (t, *J* = 7.6 Hz, 2H), 7.30 (t, *J* = 7.5 Hz, 1H),7.22 (dd, *J* = 8.7, 1.9 Hz, 2H), 6.80 (d, *J* = 8.7 Hz, 2H), 4.75 (s, 1H), 3.84 (d, *J* = 10.6 Hz, 1H), 3.77 (s, 3H), 2.92–2.74 (m, 2H), 2.40–2.30 (m, 1H), 1.97 (s, 6H), 1.96–1.88 (m, 2H), 1.81–1.71 (m, 1H), 1.54–1.27 (m, 3H) ppm; ^13^C NMR (100 MHz, CDCl_3_): *δ* 200.8, 166.4, 158.9, 148.1, 145.2, 138.5, 130.7, 128.9, 126.5, 122.5, 115.4, 113.7, 100.9, 91.7, 55.2, 47.4, 40.5, 38.4, 29.5, 28.5, 27.7, 22.5, 12.9 ppm; HRMS (ESI-TOF) *m*/*z*: [M + H]^+^ Calcd for C_28_H_29_N_3_O_5_ 488.2180; Found 488.2187.
*5-acetyl-3-methyl-4-(naphthalen-1-yl)-1-phenyl-4,5,9,10,11,12-hexahydro-8H-5,12a-epoxyazepino[2,1-b]pyrazolo[4,3-g][1,3]oxazocin-6(1H)-one* (**3de**): White solid (yield: 46.1 mg, 91%). M.P. = 183.5–183.8 °C; ^1^H NMR (400 MHz, CDCl_3_): *δ* 7.86–7.78 (m, 2H), 7.76 (d, *J* = 8.4 Hz, 2H), 7.68 (d, *J* = 7.6 Hz, 2H), 7.52–7.41 (m, 5H), 7.32 (t, *J* = 7.4 Hz, 2H), 4.98 (s, 1H), 3.89 (d, *J* = 10.6 Hz, 1H), 2.98–2.77 (m, 2H), 2.40–2.30 (m, 1H), 1.99 (s, 3H), 1.98–1.94 (br, 1H), 1.95 (s, 3H), 1.94–1.91 (br, 1H), 1.81–1.71 (m, 1H), 1.54–1.27 (m, 3H) ppm; ^13^C NMR (100 MHz, CDCl_3_): *δ* 200.5, 166.4, 148.3, 145.4, 138.5, 134.5, 133.0, 132.8, 129.0, 128.8, 128.2, 128.1, 127.6, 127.3, 126.5, 126.1, 126.1, 122.5, 115.5, 100.7, 91.8, 48.1, 40.6, 38.4, 29.6, 28.5, 27.7, 22.6, 13.0 ppm; HRMS (ESI-TOF) *m*/*z*: [M + H]^+^ Calcd for C_31_H_29_N_3_O_4_ 508.2230; Found 508.2239.
*5-acetyl-3-methyl-4-phenyl-1-(p-tolyl)-4,5,9,10,11,12-hexahydro-8H-5,12a-epoxyazepino[2,1-b]pyrazolo[4,3-g][1,3]oxazocin-6(1H)-one* (**3dg**): White solid (yield: 42.3 mg, 90%). M.P. = 191.0–191.4 °C; ^1^H NMR (400 MHz, CDCl_3_): *δ* 7.50 (d, *J* = 8.4 Hz, 2H), 7.30 (t, *J* = 6.8 Hz, 3H), 7.27–7.21 (m, 4H), 4.79 (s, 1H), 3.84 (d, *J* = 14.0 Hz, 1H), 2.90–2.74 (m, 2H), 2.40 (s, 3H), 2.34 (dd, *J* = 15.6, 10.6 Hz, 1H), 1.97 (s, 3H), 1.96 (s, 3H), 1.94–1.86 (m, 2H), 1.77–1.70 (br, 1H), 1.51–1.38 (m, 3H) ppm; ^13^C NMR (100 MHz, CDCl_3_): *δ* 200.7, 166.4, 147.8, 145.2, 137.0, 136.3, 136.0, 129.6, 129.5, 128.3, 127.6, 122.5, 115.3, 100.6, 91.6, 48.0, 40.5, 38.4, 29.5, 28.5, 27.6, 22.5, 21.1, 12.9 ppm; HRMS (ESI-TOF) *m*/*z*: [M + H]^+^ Calcd for C_28_H_29_N_3_O_4_ 472.2230; Found 472.2236.
*5-acetyl-3-methyl-1-phenyl-4-(3,4,5-trimethoxyphenyl)-4,5,9,10,11,12-hexahydro-8H-5,12a-epoxyazepino[2,1-b]pyrazolo[4,3-g][1,3]oxazocin-6(1H)-one* (**3dl**): White solid (yield: 44.8 mg, 82%). M.P. = 215.2–215.5 °C; ^1^H NMR (400 MHz, CDCl_3_): *δ* 7.61 (d, *J* = 7.6 Hz, 2H), 7.45 (t, *J* = 7.6 Hz, 2H), 7.30 (t, *J* = 7.4 Hz, 1H), 6.52 (s, 2H), 4.71 (s, 1H), 3.89–3.82 (br, 1H), 3.81 (s, 6H), 3.80 (s, 3H), 2.89–2.74 (m, 2H), 2.35 (dd, *J* = 15.6, 10.6 Hz, 1H), 2.02 (s, 3H), 1.99 (s, 3H), 1.96–1.86 (m, 2H), 1.77–1.69 (m, 1H), 1.51–1.32 (m, 3H) ppm; ^13^C NMR (100 MHz, CDCl_3_): *δ* 200.7, 166.3, 152.8, 148.2, 145.2, 138.4, 137.4, 132.5, 129.0, 126.6, 122.4, 115.5, 106.8, 100.6, 91.6, 60.8, 56.2, 48.3, 40.6, 38.4, 29.5, 28.5, 27.8, 22.6, 13.0 ppm; HRMS (ESI-TOF) *m*/*z*: [M + H]^+^ Calcd for C_30_H_33_N_3_O_7_ 548.2391; Found 548.2404.
*5-acetyl-4-(2-methoxyphenyl)-3-methyl-1-phenyl-4,5,9,10,11,12-hexahydro-8H-5,12a-epoxyazepino[2,1-b]pyrazolo[4,3-g][1,3]oxazocin-6(1H)-one* (**3dn**): White solid (yield: 39.4 mg, 81%). M.P. = 190.9–191.3 °C; ^1^H NMR (400 MHz, CDCl_3_): *δ* 7.62 (d, *J* = 7.5 Hz, 2H), 7.43 (t, *J* = 6.9 Hz, 3H), 7.28 (t, *J* = 7.4 Hz, 1H), 7.20 (td, *J* = 8.7, 1.9 Hz, 1H), 6.88 (t, *J* = 6.1 Hz, 2H), 5.53 (s, 1H), 3.92 (s, 3H), 3.85 (dd, *J* = 10.6, 3.0 Hz, 1H), 2.96–2.74 (m, 2H), 2.37–2.27 (m, 1H), 2.26 (s, 3H), 2.00 (s, 3H), 1.96–1.88 (m, 2H), 1.81–1.71 (m, 1H), 1.54–1.27 (m, 3H) ppm; ^13^C NMR (100 MHz, CDCl_3_): *δ* 199.0, 167.2, 155.4, 148.3, 145.2, 138.5, 130.9, 129.0, 128.9, 126.4, 125.5, 122.5, 121.1, 115.3, 110.5, 101.7, 91.8, 55.4, 40.4, 38.8, 38.2, 29.5, 28.5, 26.8, 22.4, 12.7 ppm; HRMS (ESI-TOF) *m*/*z*: [M + H]^+^ Calcd for C_28_H_29_N_3_O_5_ 488.2180; Found 488.2186.
*5-acetyl-4-(4-methoxyphenyl)-3-methyl-1-phenyl-4,5,8,9,10,11,12,13-octahydro-5,13a-epoxyazocino[2,1-b]pyrazolo[4,3-g][1,3]oxazocin-6(1H)-one* (**3ed**): White solid (yield: 42.5 mg, 85%). M.P. = 106.5–106.9 °C; ^1^H NMR (400 MHz, CDCl_3_): *δ* 7.62 (d, *J* = 7.5 Hz, 2H), 7.43 (t, *J* = 7.6 Hz, 2H), 7.28 (t, *J* = 7.4 Hz, 1H), 7.21 (d, *J* = 8.4 Hz, 2H), 6.80 (d, *J* = 11.6 Hz, 2H), 4.75 (s, 1H), 3.76 (s, 3H), 3.85 (dt, *J* = 14.4, 4.0 Hz, 1H), 2.78 (td, *J* = 11.9, 3.5 Hz, 1H), 2.64 (dt, *J* = 15.7, 4.3 Hz, 1H), 2.48 (td, *J* = 10.9, 4.3 Hz, 1H), 1.98 (s, 3H), 1.97 (s, 3H), 1.86–1.71 (m, 3H), 1.70–1.55 (m, 3H), 1.54–1.33 (m, 2H) ppm; ^13^C NMR (100 MHz, CDCl_3_): *δ* 200.8, 167.0, 158.9, 148.1, 145.1, 138.5, 130.7, 128.9, 128.8, 126.4, 122.3, 114.1, 113.7, 101.1, 91.6, 55.2, 47.7, 40.3, 31.7, 27.7, 27.0, 25.0, 23.8, 22.1, 12.9 ppm; HRMS (ESI-TOF) *m*/*z*: [M + H]^+^ Calcd for C_29_H_31_N_3_O_5_ 502.2336; Found 502.2341.
*5-acetyl-3-methyl-1-phenyl-4-(3,4,5-trimethoxyphenyl)-4,5,8,9,10,11,12,13-octahydro-5,13a-epoxyazocino[2,1-b]pyrazolo[4,3-g][1,3]oxazocin-6(1H)-one* (**3el**): White solid (yield: 51.0 mg, 90%). M.P. = 223.2–223.6 °C; ^1^H NMR (400 MHz, CDCl_3_): *δ* 7.60 (d, *J* = 7.8 Hz, 2H), 7.43 (t, *J* = 7.6 Hz, 2H), 7.28 (t, *J* = 7.4 Hz, 1H), 7.21 (d, *J* = 8.4 Hz, 2H), 6.50 (s, 2H), 4.71 (s, 1H), 3.81 (s, 6H), 3.80 (s, 3H), 3.64 (dt, *J* = 14.4, 3.9 Hz, 1H), 2.76 (td, *J* = 11.9, 3.5 Hz, 1H), 2.60 (dt, *J* = 15.7, 4.3 Hz, 1H), 2.53–2.41 (m, 1H), 2.01 (s, 3H), 1.98 (s, 3H), 1.84–1.70 (m, 3H), 1.68–1.53 (m, 3H), 1.52–1.35 (m, 2H) ppm; ^13^C NMR (100 MHz, CDCl_3_): *δ*200.7, 166.9, 152.8, 148.2, 145.1, 138.4, 137.4, 132.4, 129.0, 126.5, 122.2, 114.2, 106.8, 100.8, 91.4, 60.8, 56.2, 48.5, 40.4, 31.8, 27.8, 27.0, 25.0, 23.8, 22.1, 13.0 ppm; HRMS (ESI-TOF) *m*/*z*: [M + H]^+^ Calcd for C_31_H_35_N_3_O_7_ 562.2547; Found 562.2556.
*5-acetyl-4-(2-methoxyphenyl)-3-methyl-1-phenyl-4,5,8,9,10,11,12,13-octahydro-5,13a-epoxyazocino[2,1-b]pyrazolo[4,3-g][1,3]oxazocin-6(1H)-one* (**3en**): White solid (yield: 25.0 mg, 50%). M.P. = 69.6–69.9 °C; ^1^H NMR (400 MHz, CDCl_3_): *δ* 7.62 (d, *J* = 7.5 Hz, 2H), 7.43 (t, *J* = 7.7 Hz, 3H), 7.28 (t, *J* = 7.4 Hz, 1H), 7.21 (td, *J* = 8.4, 1.6 Hz, 1H), 6.92–6.85 (m, 2H), 5.51 (s, 1H), 3.93 (s, 3H), 3.67 (dt, *J* = 14.4, 4.0 Hz, 1H), 2.80 (td, *J* = 11.9, 3.5 Hz, 1H), 2.65 (dq, *J* = 15.7, 3.6 Hz, 1H), 2.44 (td, *J* = 10.9, 4.3 Hz, 1H), 2.24 (s, 3H), 1.99 (s, 3H), 1.86–1.69 (m, 3H), 1.64–1.55 (m, 3H), 1.54–1.39 (m, 2H) ppm; ^13^C NMR (100 MHz, CDCl_3_): *δ* 198.7, 167.9, 155.5, 148.3, 145.1, 138.6, 131.0, 128.9, 128.9, 126.3, 125.3, 122.3, 121.1, 114.0, 110.5, 101.9, 91.5, 55.4, 40.1, 39.3, 32.0, 26.9, 26.8, 25.3, 23.9, 22.1, 12.7 ppm; HRMS (ESI-TOF) *m*/*z*: [M + H]^+^ Calcd for C_21_H_31_N_3_O_5_ 502.2336; Found 502.2338.
*7-benzoyl-12-benzyl-2,3,7,12-tetrahydro-1H-6,13a-epoxypyrrolo[2′,1′:2,3][1,3]oxazocino[8,7-b]indol-5(6H)-one* (**3bq**): White solid (yield: 42.2 mg, 91%). M.P. = 114.5–114.8 °C; ^1^H NMR (400 MHz, CDCl_3_): *δ* 7.38–7.29 (m, 6H), 7.22 (t, *J* = 3.56 Hz, 2H), 7.14 (dd, *J* = 7.4, 0.7 Hz, 1H), 7.07 (d, *J* = 7.8 Hz, 1H), 7.01 (td, *J* = 7.7, 1.2 Hz, 1H), 6.89 (td, *J* = 7.7, 1.2Hz, 1H), 6.47 (d, *J* = 7.8 Hz, 2H), 5.37 (s, 1H), 5.03 (d, *J* = 15.1 Hz, 1H), 4.63 (d, *J* = 15.4 Hz, 1H), 4.59 (s, 1H), 3.83–3.75 (m, 1H), 3.22–3.15 (m, 1H), 2.12–2.03 (m, 1H), 1.89–1.80 (m, 2H), 1.26–1.19 (m, 1H) ppm; ^13^C NMR (100 MHz, CDCl_3_): *δ* 195.4, 173.5, 173.5, 142.5, 136.3, 135.5, 133.0, 128.9, 128.9, 128.3, 127.9, 127.6, 127.4, 126.3, 125.4, 123.0, 108.5, 108.4, 81.4, 59.6, 56.6, 44.3, 42.6, 26.3, 25.8 ppm; HRMS (ESI-TOF) *m*/*z*: [M + H]^+^ Calcd for C_29_H_24_N_2_O_4_ 465.1809; Found 465.1761.

## 4. Conclusions

Conclusively, under the catalysis of Rh_2_(OAc)_4_ and binapbisphosphine ligand (±)-**L3** in DCE at 80 °C, the cascade cyclization of diazoimides with alkylidenepyrazolones underwent diastereoselectively (dr > 20:1), affording pyrazole-fused oxa-bridged oxazocines in reasonable chemical yields. Moreover, the design and exploration of other novel cyclic carbonyl-ylide-based cyloadditions is ongoing in our organic lab, and will be reported in due course.

## Figures and Tables

**Table 1:**
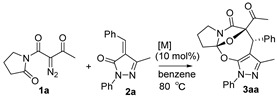
Screening of metal-catalysts ^[a]^.

Entry	[M]	Time (h)	Yield ^[b]^ (%)	dr ^[c]^
1	Ru(bpy)_3_Cl_2_·6H_2_O	10	trace	−
2	Ru(OAc)_3_	10	43	>20:1
3	Rh_2_(Oct)_4_	10	43	>20:1
4	Pd_2_(dba)_3_	10	66	>20:1
5	Pd(PPh_3_)_2_Cl_2_	10	75	>20:1
6	Rh(OAc)_3_	10	70	>20:1
7	Rh_2_(OAc)_4_	2.0	82	>20:1
8 ^[d]^	Rh_2_(OAc)_4_	12	NR ^[e]^	−

^[a]^ Reactions were carried out with **1a** (0.15 mmol), **2a** (0.1 mmol) and [M] (10 mol%) in benzene (1.5 mL) at 80 °C. ^[b]^ Isolated yield. ^[c]^ Determined by ^1^H NMR. ^[d]^ Reactions were carried out with **1a** (0.15 mmol), **2a** (0.1 mmol) and [M] (10 mol%) in benzene (1.5 mL) at 60 °C. ^[e]^ No reaction.

**Table 2:**
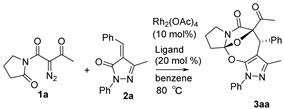

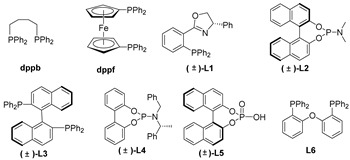
Screening of mono- and bisphosphine ligands ^[a]^.

Entry	Ligand	Time (h)	Yield ^[b]^ (%)	dr ^[c]^
1	-	2.0	82	>20:1
2	dppb	6.0	73	>20:1
3	dppf	10	trace	−
4	(±)-**L1**	10	trace	−
5	(±)-**L2**	6.0	77	>20:1
6	(±)-**L3**	1.0	88	>20:1
7	(±)-**L4**	10	trace	−
8	(±)-**L5**	1.0	78	>20:1
9	**L6**	10	trace	−

^[a]^ Reactions were carried out with **1a** (0.15 mmol), **2a** (0.1 mmol), Rh_2_(OAc)_4_ (10 mol%), and ligand (20 mol%) in benzene (1.5 mL) at 80 °C. ^[b]^ Isolated yield. ^[c]^ Determined by ^1^H NMR.

**Table 3:**
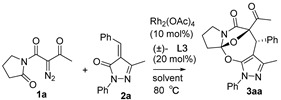
Screening of solvents ^[a]^.

Entry	Solvent	Time (h)	Yield ^[b]^ (%)	dr ^[c]^
1	benzene	1.0	88	>20:1
2	1,4-dioxane	5.0	22	>20:1
3	THF	5.0	35	>20:1
4	toluene	0.5	73	>20:1
5	PhCF_3_	1.0	80	>20:1
6	DMF	10	NR ^[d]^	−
7	HFIP	10	trace	−
8	1,2-DCE	1.0	98	>20:1
9	DCM	10	trace	−

^[a]^ Reactions were carried out with **1a** (0.15 mmol), **2a** (0.1 mmol), Rh_2_(OAc)_4_ (10 mol%), and (±)-**L3** (20 mol%) in solvent (1.5 mL) at 80 °C. ^[b]^ Isolated chemical yield. ^[c]^ Determined by ^1^H NMR. ^[d]^ No reaction.

**Table 4:**
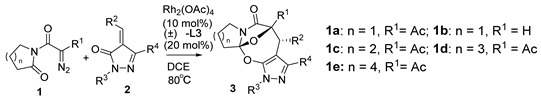
Substrate scope ^[a]^.

Entry	1	2 (R^2^, R^3^, R^4^)	3	Time (h)	Yield ^[b]^ (%)	dr ^[c]^
1	**1a**	**2a** (C_6_H_5_, C_6_H_5_, Me)	**3aa**	1.0	98	>20:1
2	**1a**	**2b** (4-BrC_6_H_4_, C_6_H_5_, Me)	**3ab**	0.5	73	>20:1
3	**1a**	**2c** (4-NO_2_C_6_H_4_, C_6_H_5_, Me)	**3ac**	0.5	65	>20:1
4	**1a**	**2d** (4-MeOC_6_H_4_, C_6_H_5_, Me)	**3ad**	1.0	82	>20:1
5	**1a**	**2e** (2-Naphthyl, C_6_H_5_, Me)	**3ae**	0.5	80	>20:1
6	**1a**	**2f** (C_6_H_5_, 4-ClC_6_H_4_, Me)	**3af**	0.5	72	>20:1
7	**1a**	**2g** (C_6_H_5_, 4-MeC_6_H_4_, Me)	**3ag**	1.0	80	>20:1
8	**1a**	**2h** (C_6_H_5_, C_6_H_5_, C_6_H_5_)	**3ah**	1.0	88	>20:1
9	**1a**	**2i** (C_6_H_5_, Me, C_6_H_5_)	**3ai**	24	NR ^[d]^	−
10	**1a**	**2j** (C_6_H_5_, Me, Me)	**3aj**	24	NR ^[d]^	−
11	**1a**	**2k** (C_6_H_5_, Me, H)	**3ak**	24	NR ^[d]^	−
12	**1b**	**2a** (C_6_H_5_, C_6_H_5_, Me)	**3ba**	0.1	77	>20:1
13	**1c**	**2a** (C_6_H_5_, C_6_H_5_, Me)	**3ca**	0.5	70	>20:1
14	**1d**	**2a** (C_6_H_5_, C_6_H_5_, Me)	**3da**	0.5	75	>20:1
15	**1e**	**2a** (C_6_H_5_, C_6_H_5_, Me)	**3ea**	0.5	82	>20:1
16	**1f** 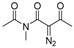	**2a** (C_6_H_5_, C_6_H_5_, Me)	**3fa** 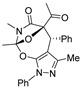	16	35	>20:1
17	**1g** 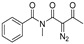	**2a** (C_6_H_5_, C_6_H_5_, Me)	**3ga** 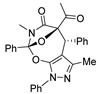	24	NR ^[d]^	−
18	**1b**	**2b** (4-BrC_6_H_4_, C_6_H_5_, Me)	**3bb**	0.1	90	>20:1
19	**1b**	**2c** (4-NO_2_C_6_H_4_, C_6_H_5_, Me)	**3bc**	0.1	80	>20:1
20	**1b**	**2l** (3,4,5-*tri*-MeOC_6_H_2_, C_6_H_5_, Me)	**3bl**	0.1	77	>20:1
21	**1b**	**2m** (4-CF_3_C_6_H_4_, C_6_H_5_, Me)	**3bm**	0.1	88	>20:1
22	**1b**	**2n** (2-MeOC_6_H_4_, C_6_H_5_, Me)	**3bn**	0.1	93	>20:1
23	**1b**	**2o** 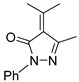	**3bo** 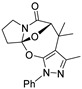	0.1	95	>20:1
24	**1c**	**2c** (4-NO_2_C_6_H_4_, C_6_H_5_, Me)	**3cc**	0.3	80	>20:1
25	**1c**	**2d** (4-MeOC_6_H_4_, C_6_H_5_, Me)	**3cd**	0.3	90	>20:1
26	**1c**	**2e** (2-Naphthyl, C_6_H_5_, Me)	**3ce**	1.0	95	>20:1
27	**1c**	**2f** (C_6_H_5_, 4-ClC_6_H_4_, Me)	**3cf**	1.0	80	>20:1
28	**1c**	**2g** (C_6_H_5_, 4-MeC_6_H_4_, Me)	**3cg**	1.0	84	>20:1
29	**1c**	**2l** (3,4,5-*tri*-MeOC_6_H_2_, C_6_H_5_, Me)	**3cl**	1.0	75	>20:1
30	**1c**	**2n** (2-MeOC_6_H_4_, C_6_H_5_, Me)	**3cn**	1.0	86	>20:1
31	**1c**	**2p** (2-Thienyl, C_6_H_5_, Me)	**3cp**	1.0	85	>20:1
32	**1d**	**2b** (4-BrC_6_H_4_, C_6_H_5_, Me)	**3db**	1.0	60	>20:1
33	**1d**	**2d** (4-MeOC_6_H_4_, C_6_H_5_, Me)	**3dd**	1.0	78	>20:1
34	**1d**	**2e** (2-Naphthyl, C_6_H_5_, Me)	**3de**	1.0	91	>20:1
35	**1d**	**2g** (C_6_H_5_, 4-MeC_6_H_4_, Me)	**3dg**	1.0	90	>20:1
36	**1d**	**2l** (3,4,5-*tri*-MeOC_6_H_2_, C_6_H_5_, Me)	**3dl**	1.0	82	>20:1
37	**1d**	**2n** (2-MeOC_6_H_4_, C_6_H_5_, Me)	**3dn**	1.0	81	>20:1
38	**1e**	**2d** (4-MeOC_6_H_4_, C_6_H_5_, Me)	**3ed**	1.0	85	>20:1
39	**1e**	**2l** (3,4,5-*tri*-MeOC_6_H_2_, C_6_H_5_, Me)	**3el**	1.0	90	>20:1
40	**1e**	**2n** (2-MeOC_6_H_4_, C_6_H_5_, Me)	**3en**	1.0	50	>20:1
41	**1b**	**2q** 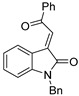	**3bq** 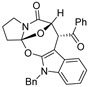	1.0	91	>20:1

^[a]^ Reactions were carried out with **1** (0.15 mmol), **2** (0.1 mmol), Rh_2_(OAc)_4_ (10 mol%), and (±)-**L3** (20 mol%) in DCE (1.5 mL) at 80 °C. ^[b]^ Isolated chemical yield. ^[c]^ Determined by ^1^H NMR. ^[d]^ No reaction.

## Data Availability

Not applicable.

## Supplementary Materials

The following supporting information can be downloaded at: https://www.mdpi.com/article/10.3390/molecules28207021/s1, NMR spectral copies of products **3**–**7**. X-ray crystal data of products **3aa** and **4**.

## Sample Availability

Samples of the compounds are available from the authors.
