# Supplementary material for: Diastereoselective Cascade Cyclization of Diazoimides with Alkylidene Pyrazolones for Preparation of Pyrazole-Fused Oxa-Bridged Oxazocines
Source: Molecules. 2023 Oct 10;28(20):7021. doi: 10.3390/molecules28207021 (PMC10609588; doi:10.3390/molecules28207021)
Supplement: Supplementary file 1 [file molecules-28-07021-s001.zip › molecules-2635666-supplementary.pdf]

Supporting Information

Diastereoselective Cascade Cyclization of Diazoimides with Alkylidene Pyrazolones for Preparation of Pyrazole-Fused Oxa-Bridged Oxazocines

Table of Contents

Supporting Information **1**

Table of Contents **1**

1. NMR Spectra of Products 3-7 .... **2**

2. X-Ray Crystal Data of Compound 3aa and 4 .... **47**

1. NMR Spectra of Products 3-7

5-acetyl-3-methyl-1,4-diphenyl-4,5,9,10-tetrahydro-8H-5,10a-epoxypyrazolo[4,3-g]pyrrolo[2,1-b][1,3]oxazocin-6(1H)-one (3aa):

^{1}H NMR (400MHz,$CDCl_3$) of 3aa

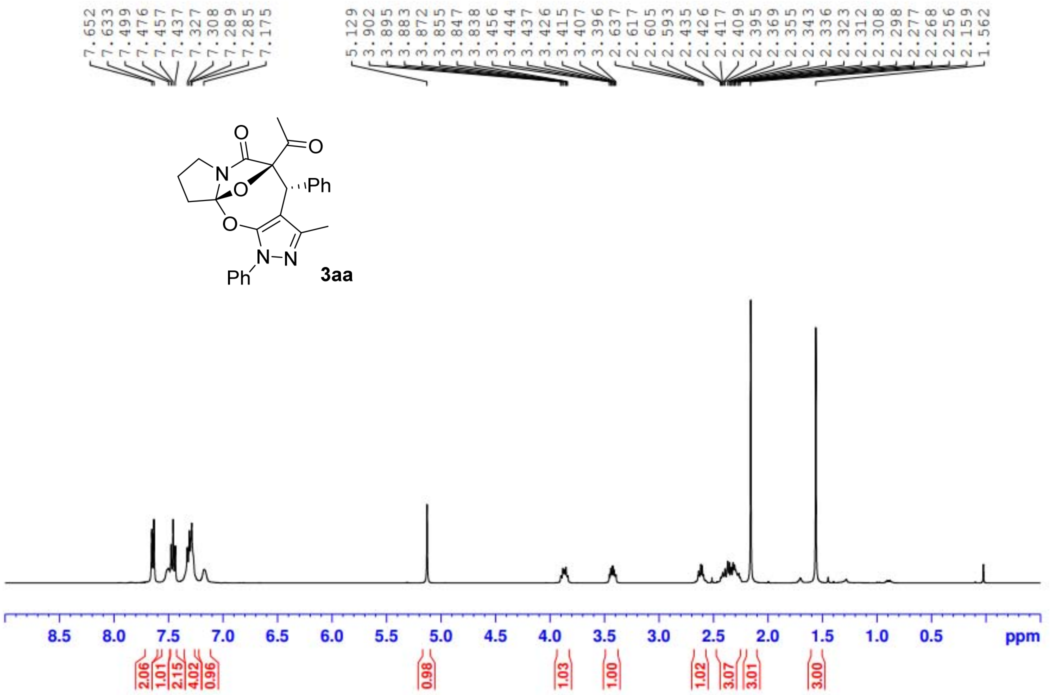

^{13}C NMR (100MHz,$CDCl_3$) of 3aa

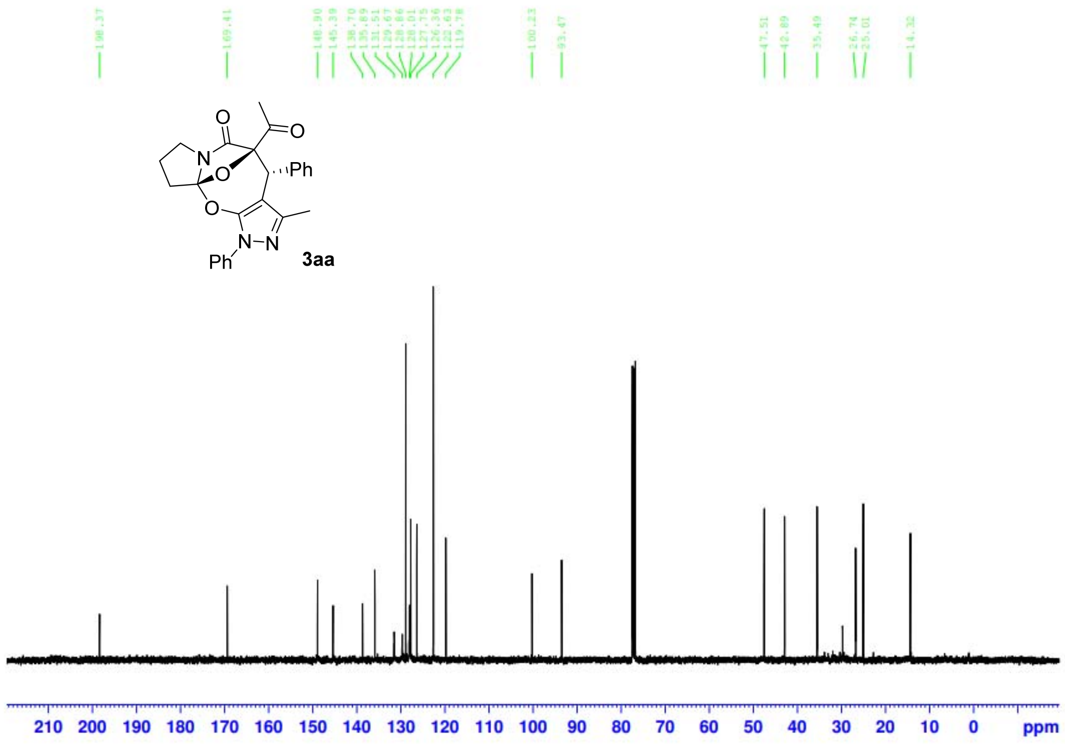

Dept-135 of 3aa

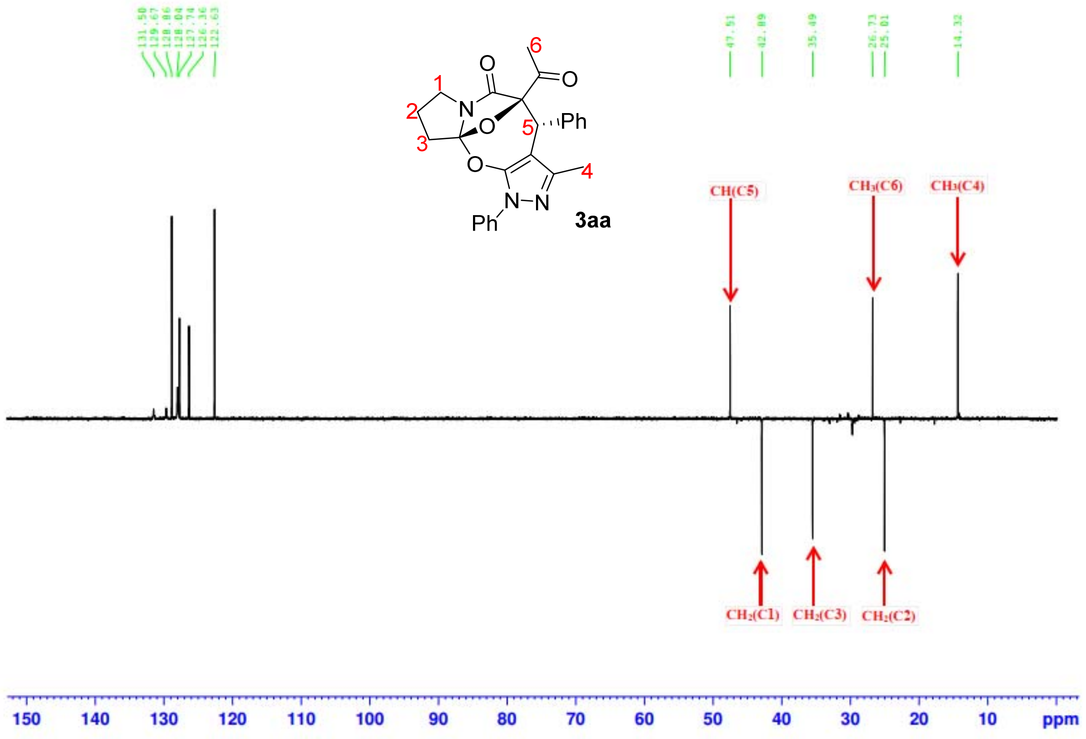

HSQC of 3aa

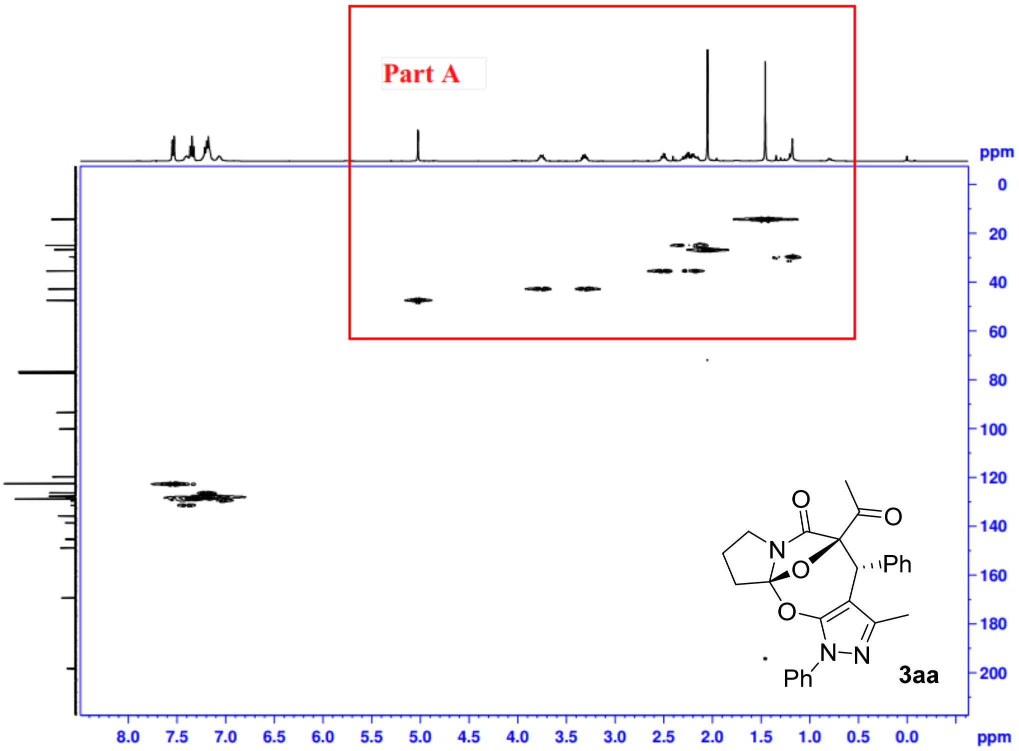

HSQC of Part A

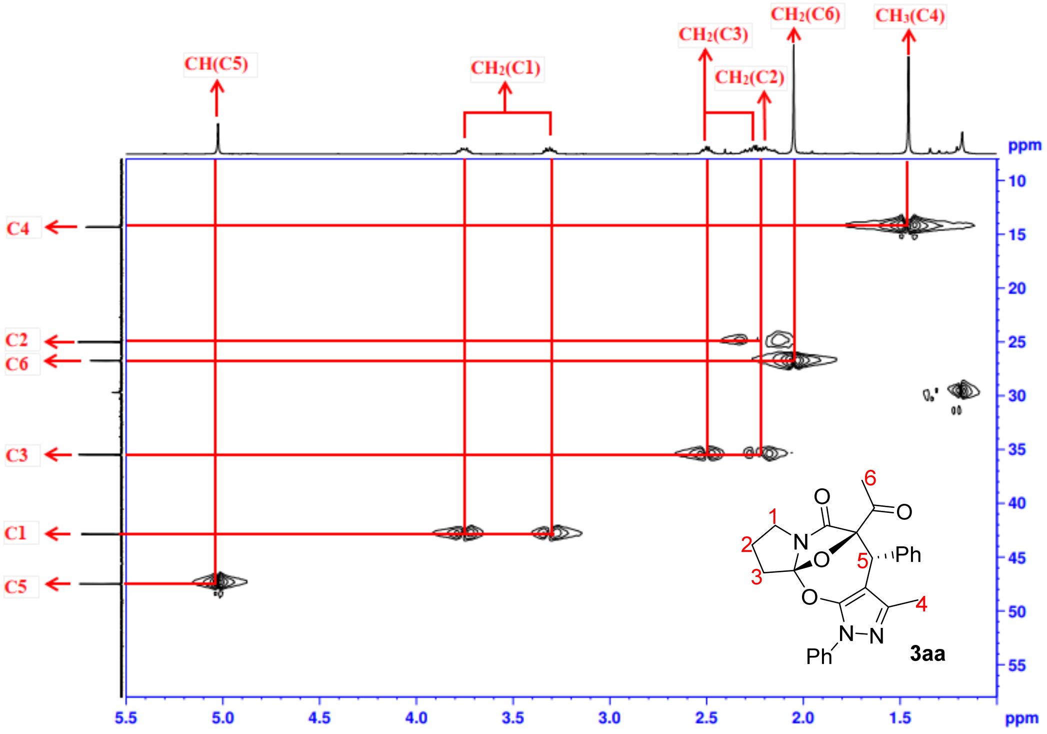

5-acetyl-4-(4-bromophenyl)-3-methyl-1-phenyl-4,5,9,10-tetrahydro-8H-5,10a-epoxypyrazolo[4,3-g]pyrrolo[2,1-b][1,3]oxazocin-6(1H)-one (3ab):

^{1}H NMR (400MHz,$CDCl_3$) of 3ab

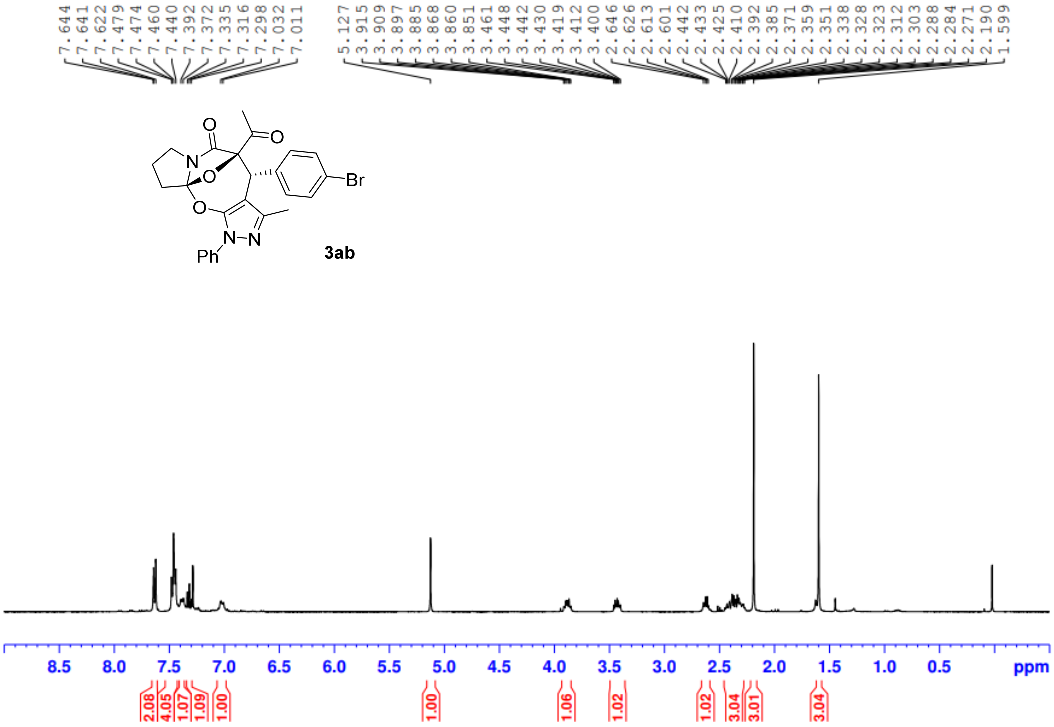

^{13}C NMR (100MHz,$CDCl_3$) of 3ab

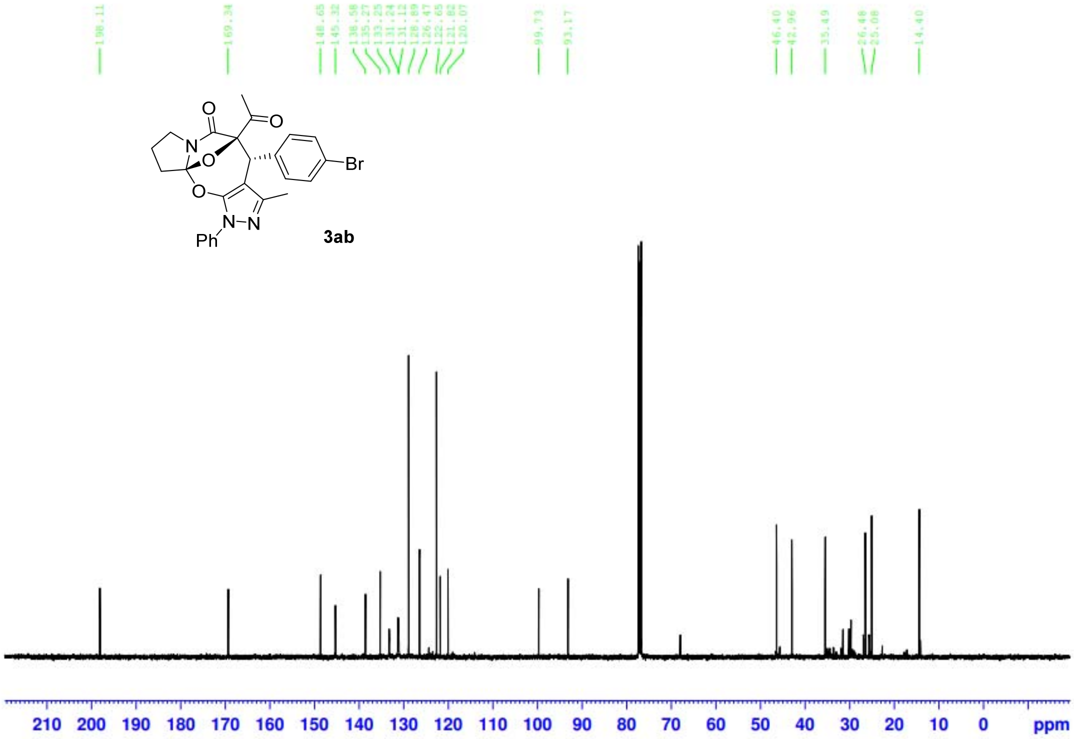

5-acetyl-3-methyl-4-(4-nitrophenyl)-1-phenyl-4,5,9,10-tetrahydro-8H-5,10a-epoxypyrazolo[4,3-g]pyrrolo[2,1-b][1,3]oxazocin-6(1H)-one (3ac):

^{1}H NMR (400MHz,$CDCl_3$) of 3ac

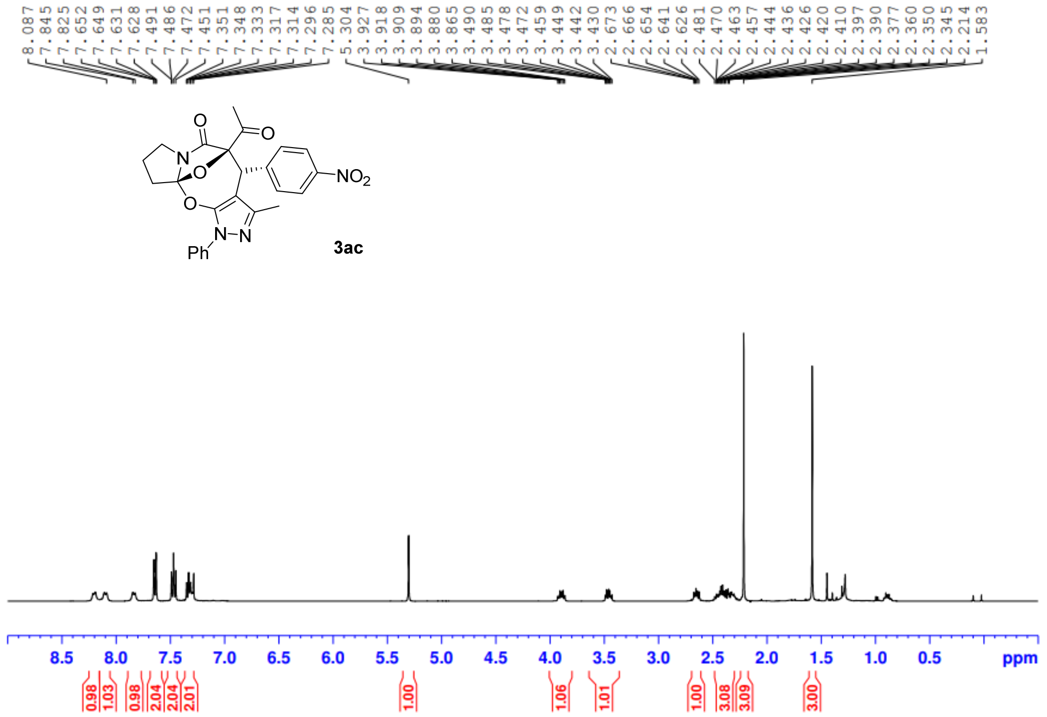

^{13}C NMR (100MHz,$CDCl_3$) of 3ac

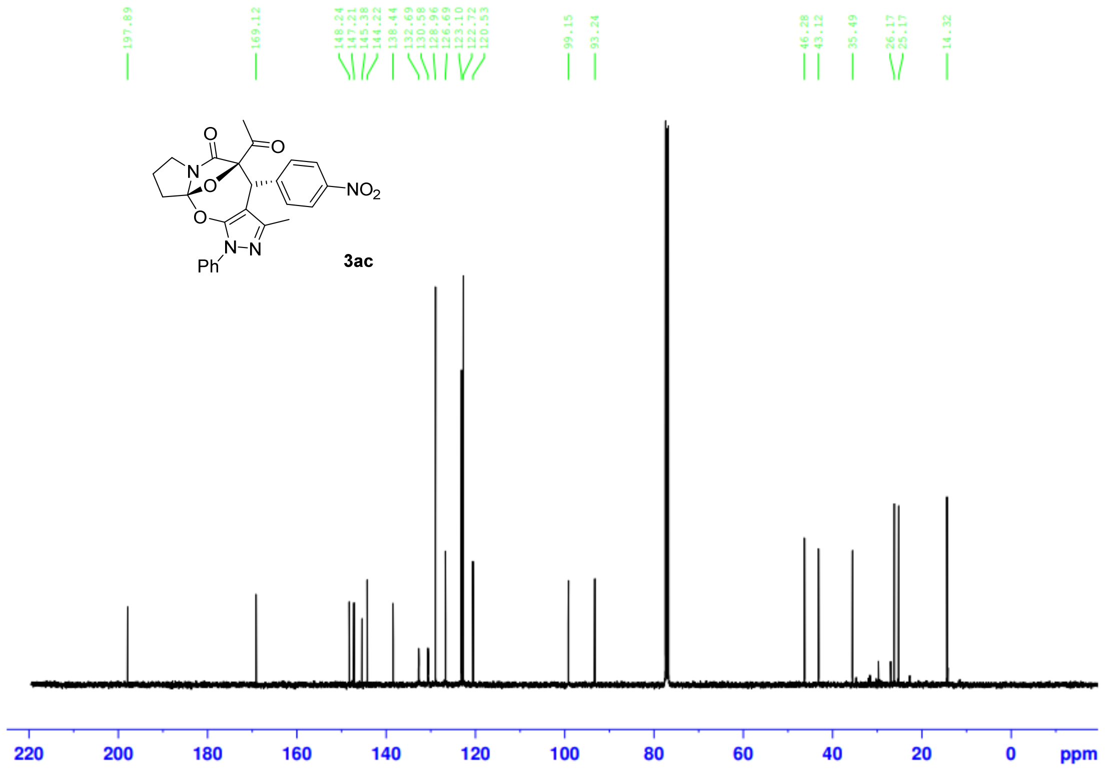

5-acetyl-4-(4-methoxyphenyl)-3-methyl-1-phenyl-4,5,9,10-tetrahydro-8H-5,10a-epoxypyrazolo[4,3-g]pyrrolo[2,1-b][1,3]oxazocin-6(1H)-one (3ad):

^{1}H NMR (400MHz,CDCl$_3$) of 3ad

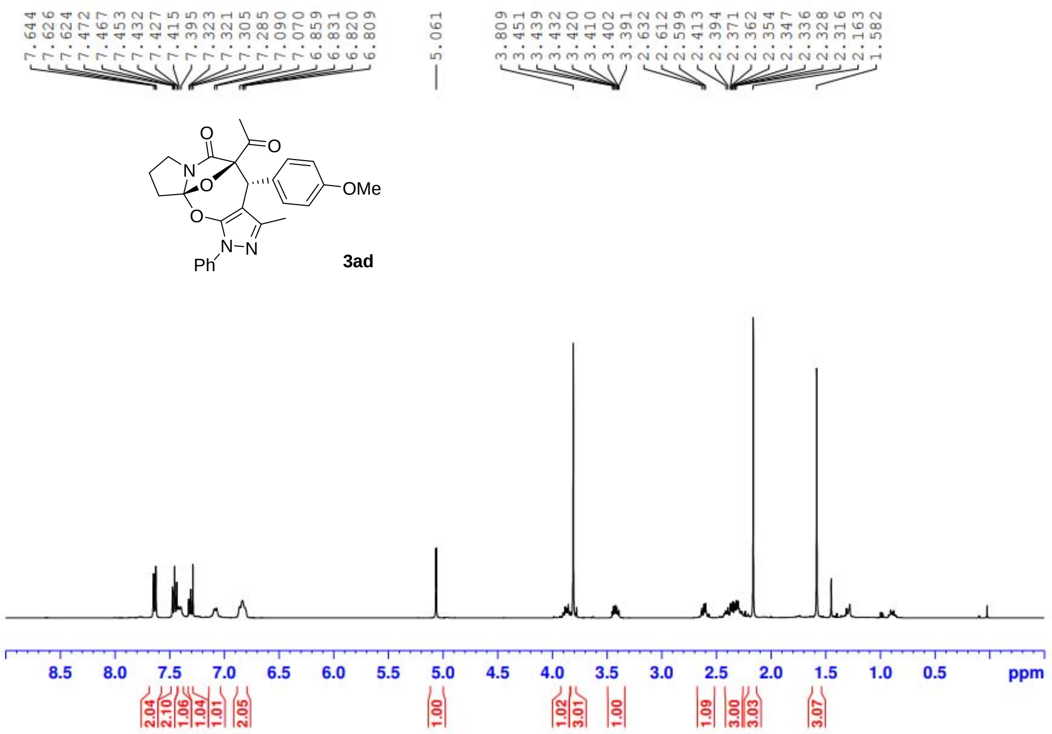

^{13}C NMR (100MHz,CDCl$_3$) of 3ad

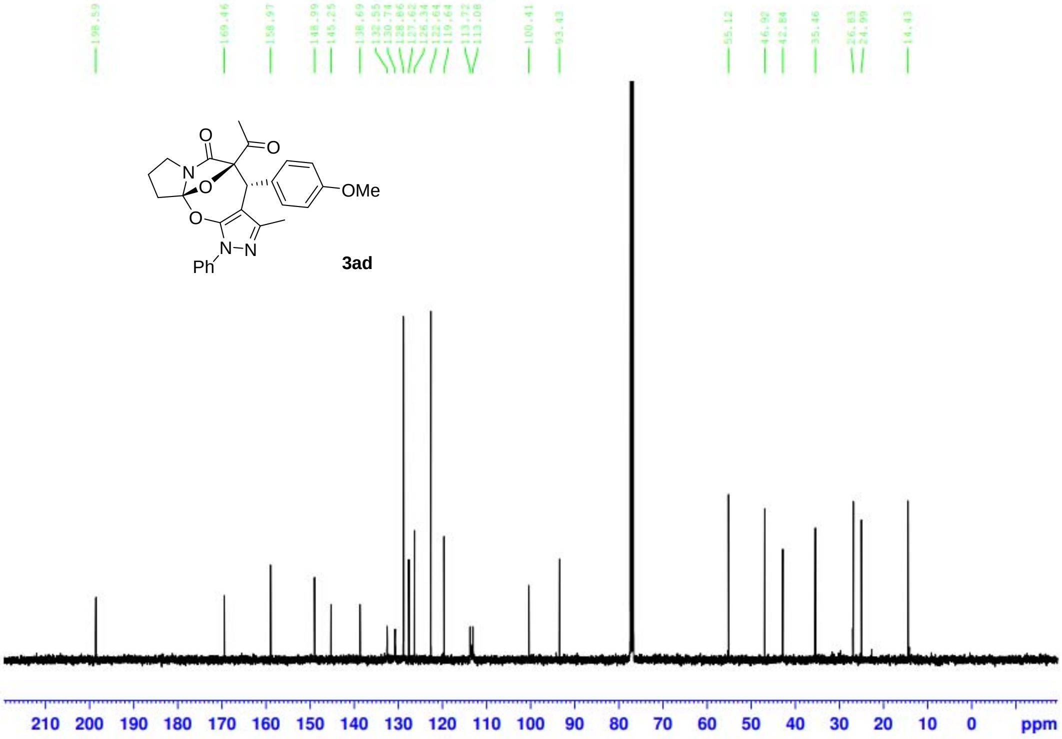

5-acetyl-3-methyl-4-(naphthalen-2-yl)-1-phenyl-4,5,9,10-tetrahydro-8H-5,10a-epoxypyrazolo [4,3-g]pyrrolo[2,1-b][1,3]oxazocin-6(1H)-one（3ae）:

^{1}H NMR (400MHz,CDCl$_3$) of 3ae

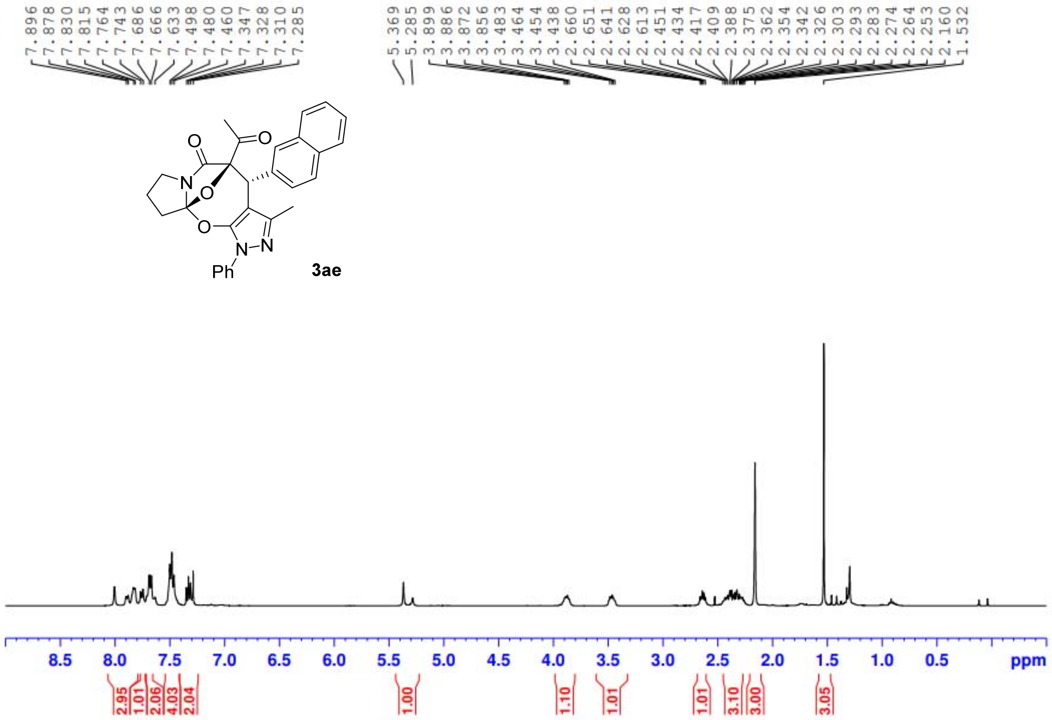

^{13}C NMR (100MHz,CDCl$_3$) of 3ae

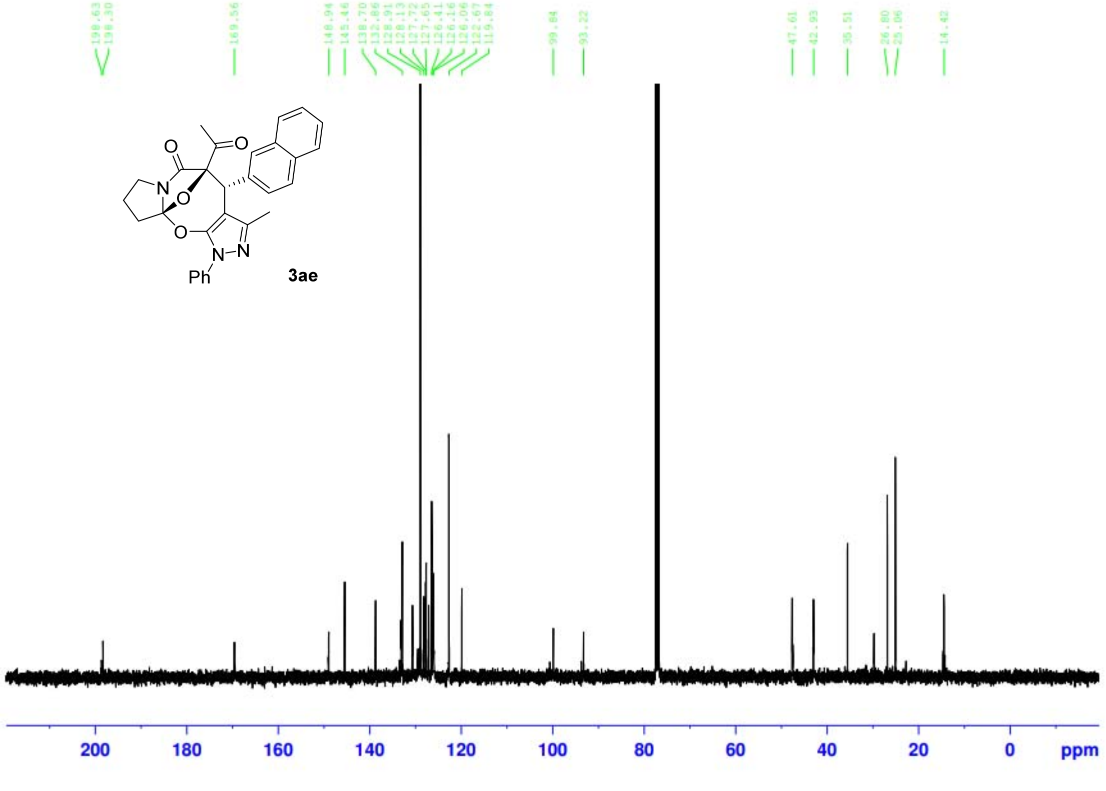

5-acetyl-1-(4-chlorophenyl)-3-methyl-4-phenyl-4,5,9,10-tetrahydro-8H-5,10a-epoxypyrazolo[4,3-g]pyrrolo[2,1-b][1,3]oxazocin-6(1H)-one (3af):

^{1}H NMR (400MHz,CDCl$_3$) of 3af

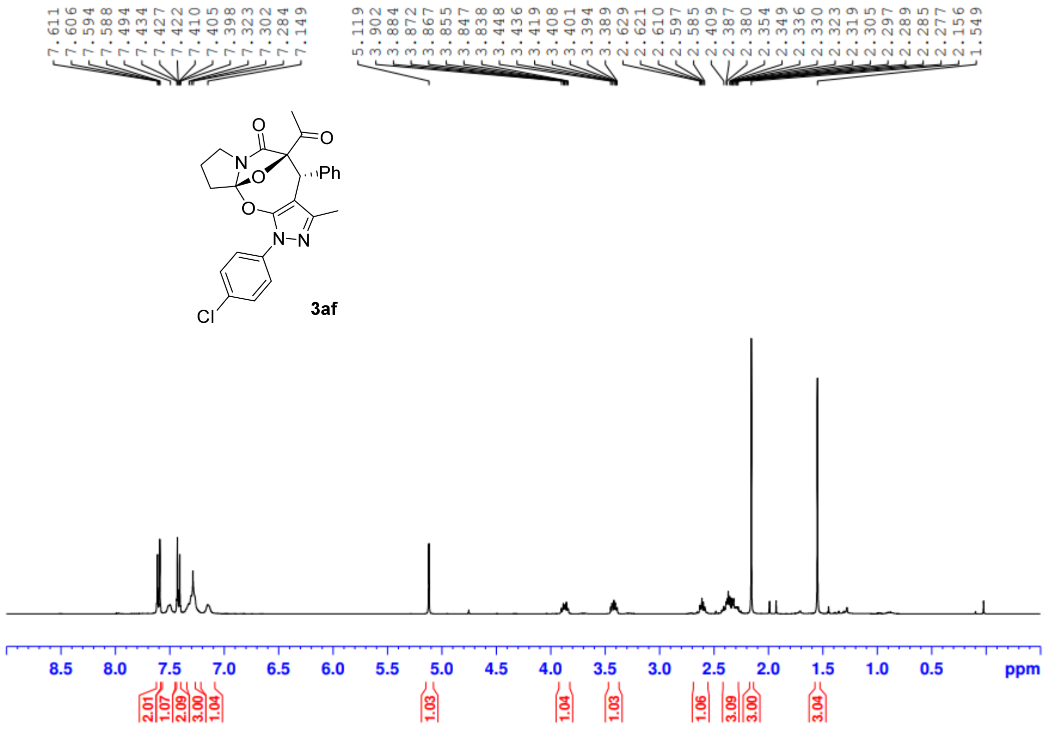

^{13}C NMR (100MHz,CDCl$_3$) of 3af

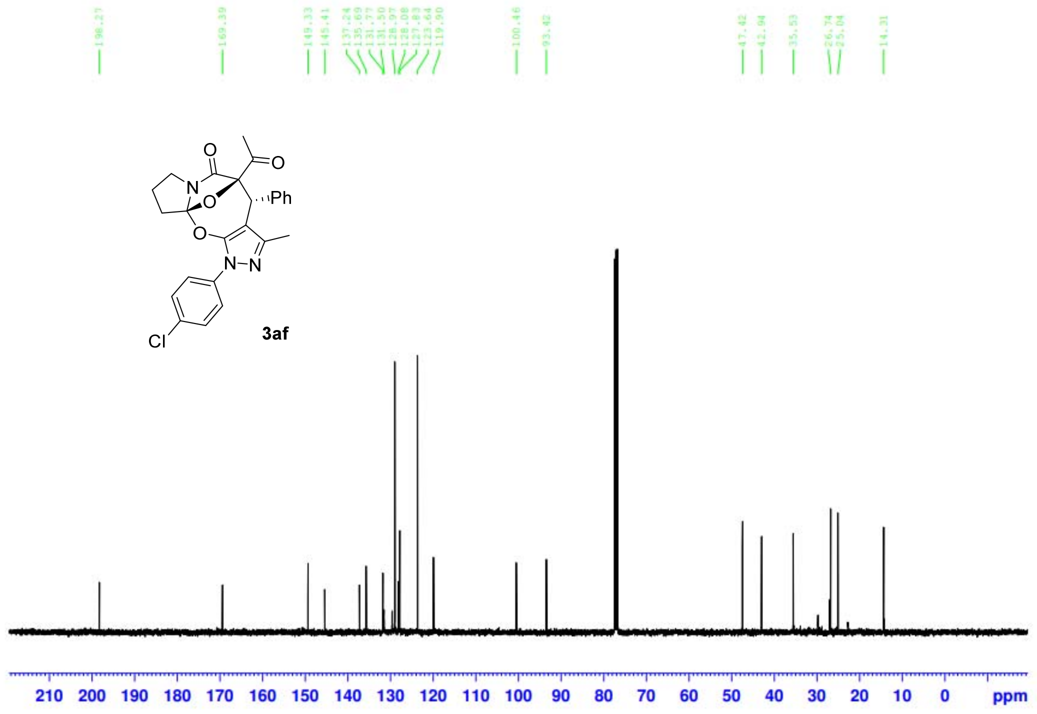

5-acetyl-3-methyl-4-phenyl-1-(p-tolyl)-4,5,9,10-tetrahydro-8H-5,10a-epoxypyrazolo[4,3-g]pyrrolo[2,1-b][1,3]oxazocin-6(1H)-one (3ag):

^{1}H NMR (400MHz,CDCl$_3$) of 3ag

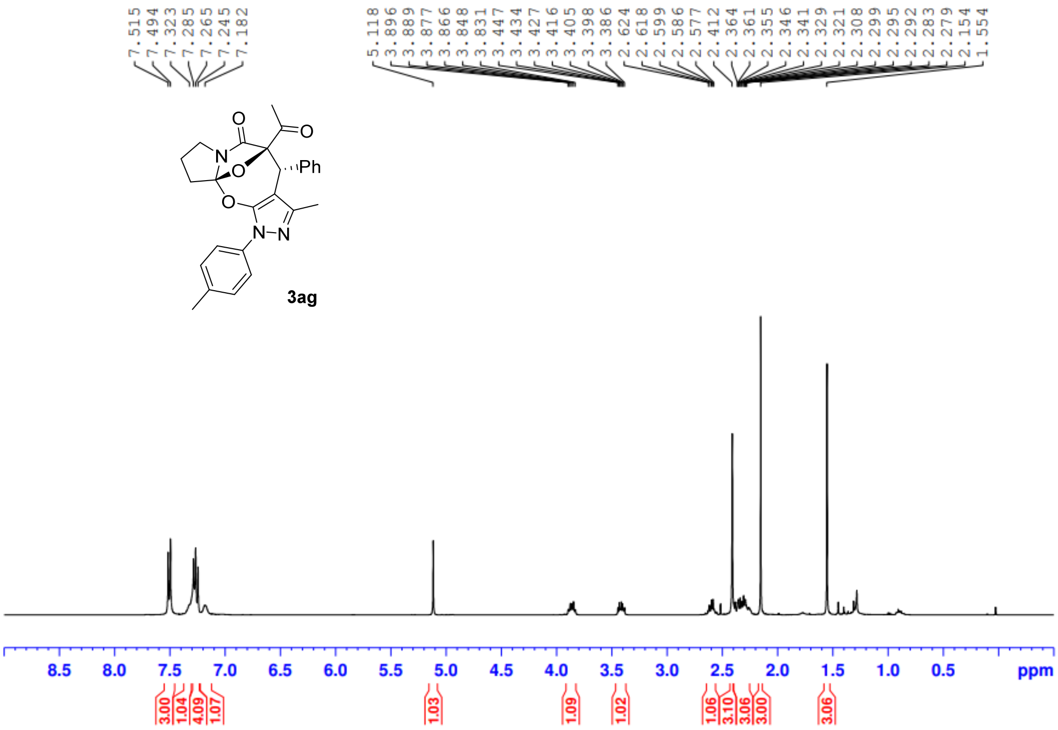

^{13}C NMR (100MHz,CDCl$_3$) of 3ag

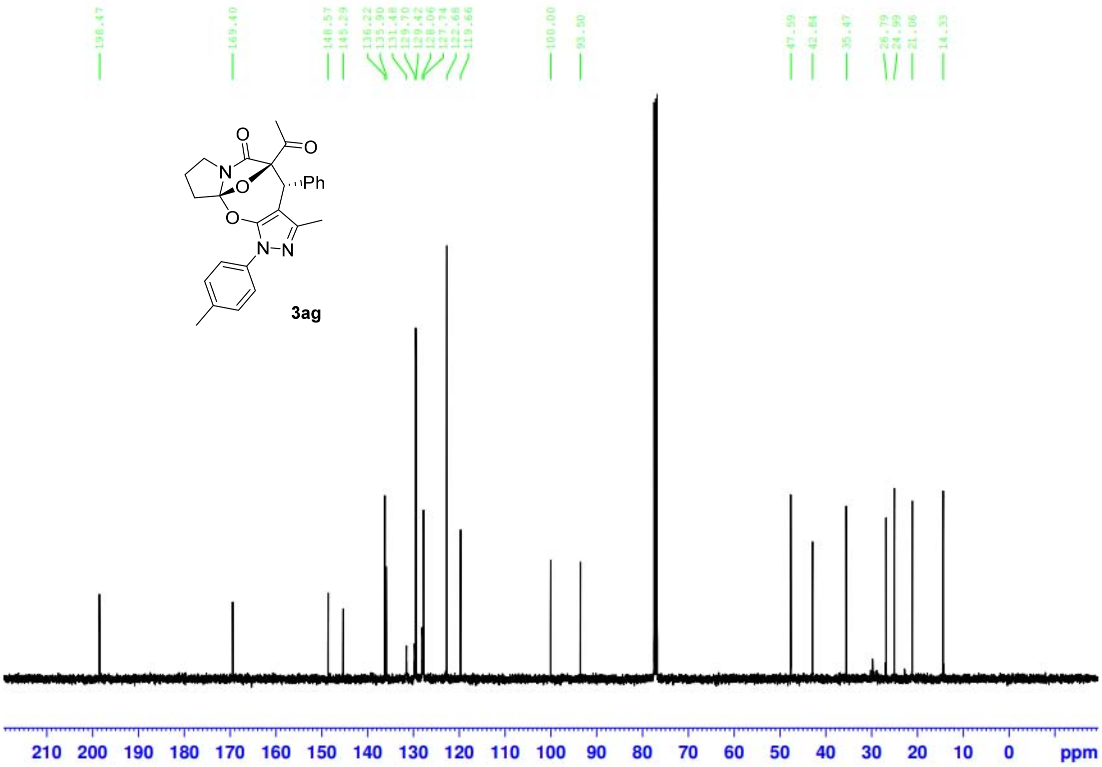

5-acetyl-1,3,4-triphenyl-4,5,9,10-tetrahydro-8H-5,10a-epoxypyrazolo[4,3-g]pyrrolo[2,1-b][1,3]oxazocin-6(1H)-one (3ah):

^{1}H NMR (400MHz,$CDCl_3$) of 3ah

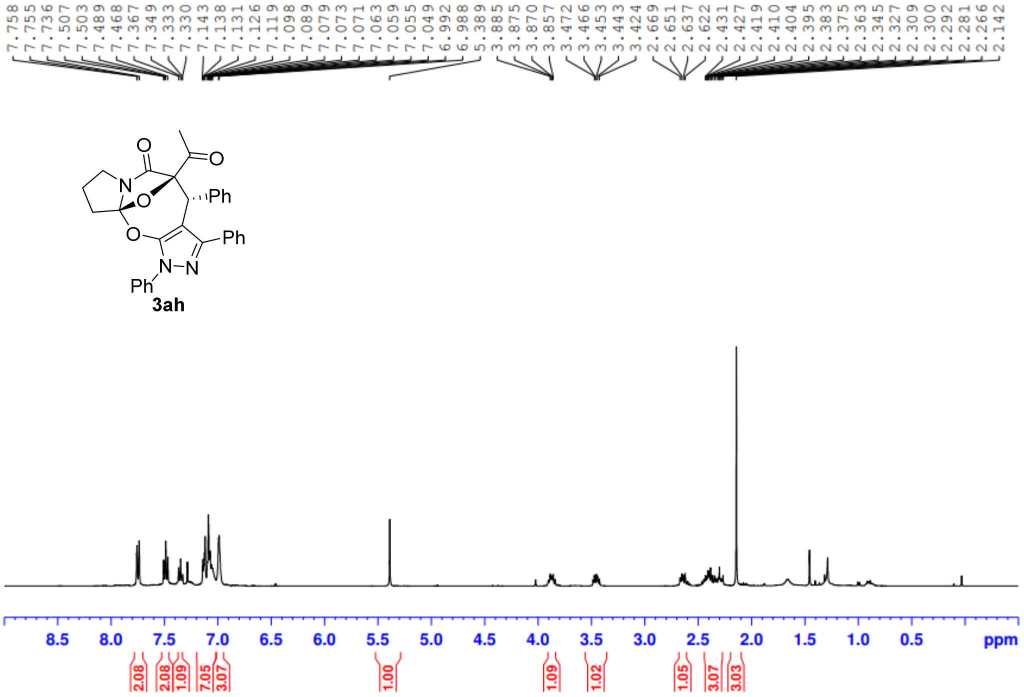

^{13}C NMR (100MHz,$CDCl_3$) of 3ah

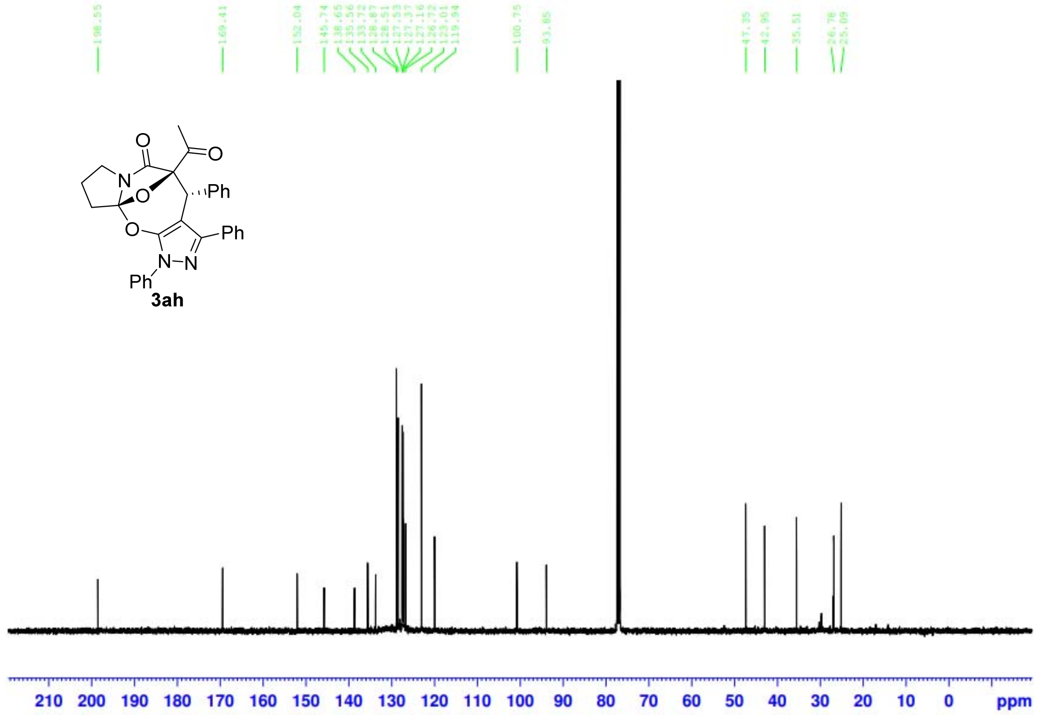

3-methyl-1,4-diphenyl-4,5,9,10-tetrahydro-8H-5,10a-epoxypyrazolo[4,3-g]pyrrolo[2,1-b][1,3] oxazocin-6(1H)-one (3ba):

^{1}H NMR (400MHz,$CDCl_3$) of 3ba

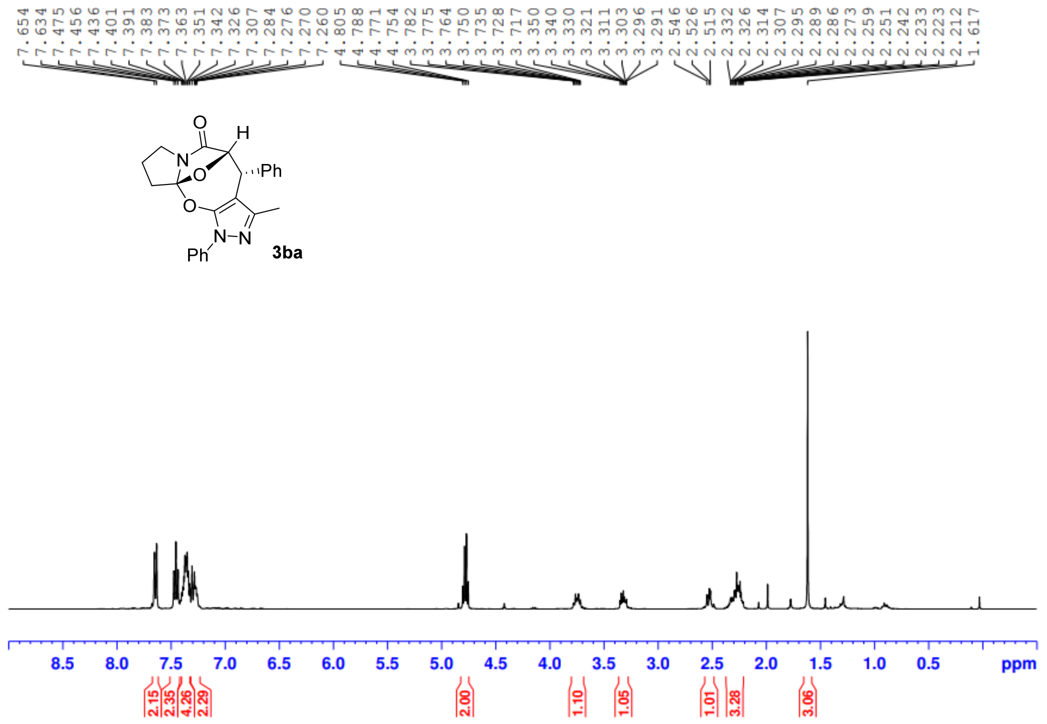

^{13}C NMR (100MHz,$CDCl_3$) of 3ba

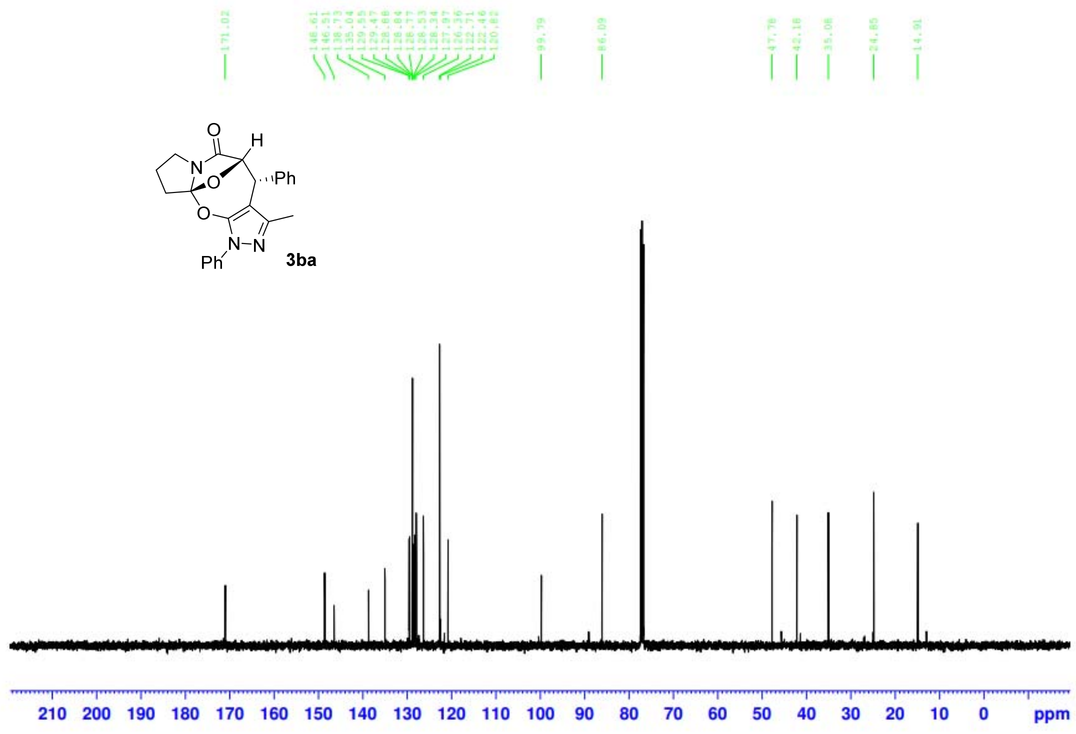

5-acetyl-3-methyl-1,4-diphenyl-4,5,8,9,10,11-hexahydro-5,11a-epoxypyrazolo[4,3-g]pyrido[2,1-b][1,3]oxazocin-6(1H)-one (3ca):

^{1}H NMR (400MHz,CDCl$_3$) of 3ca

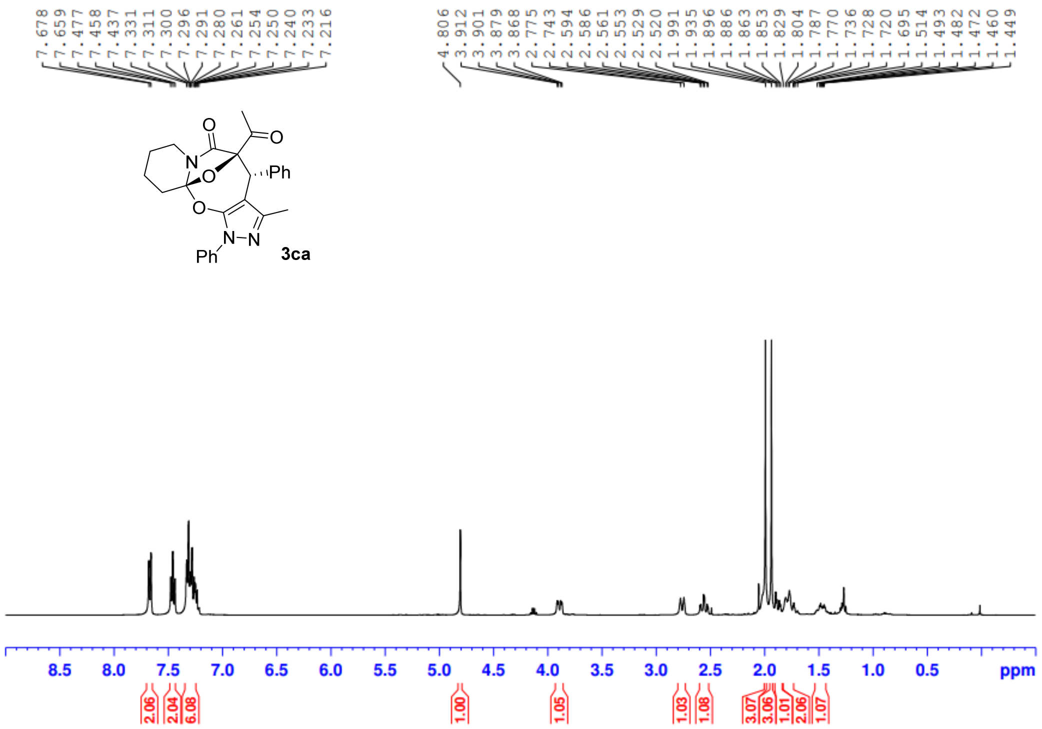

^{13}C NMR (100MHz,CDCl$_3$) of 3ca

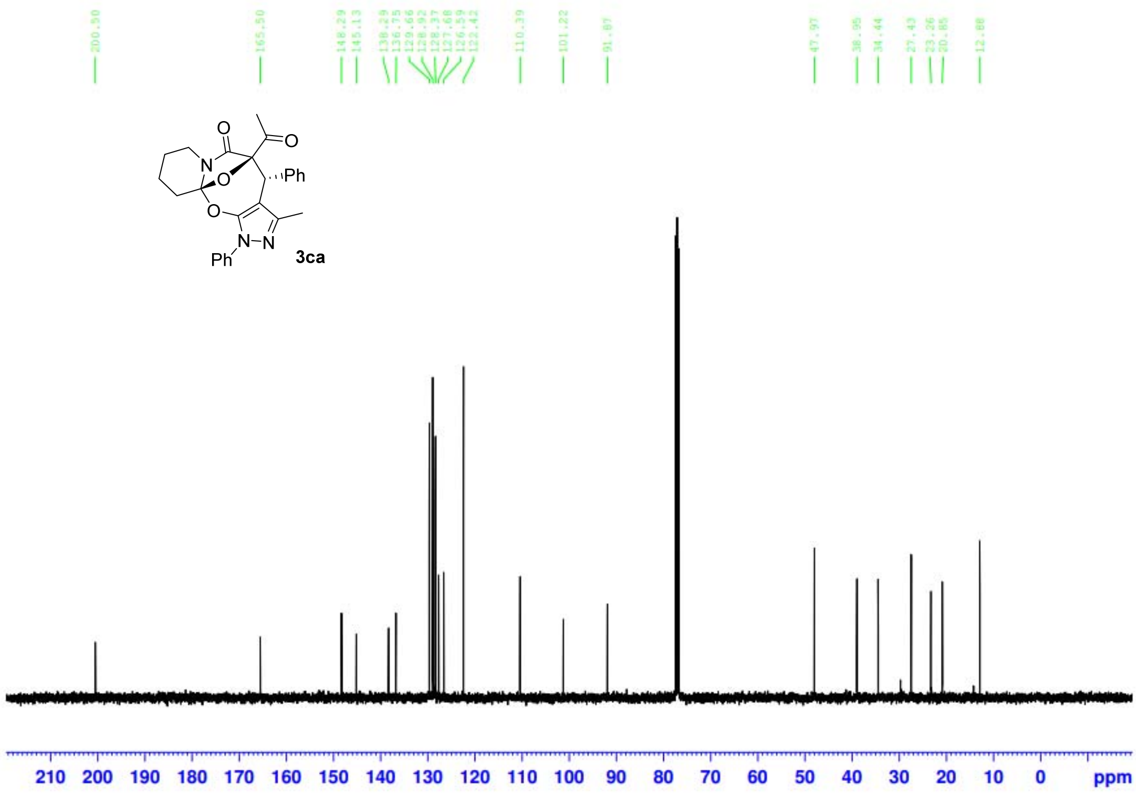

5-acetyl-3-methyl-1,4-diphenyl-4,5,9,10,11,12-hexahydro-8H-5,12a-epoxyazepino[2,1-b]pyrazolo[4,3-g][1,3]oxazocin-6(1H)-one (3da):

^{1}H NMR (400MHz,$CDCl_3$) of 3da

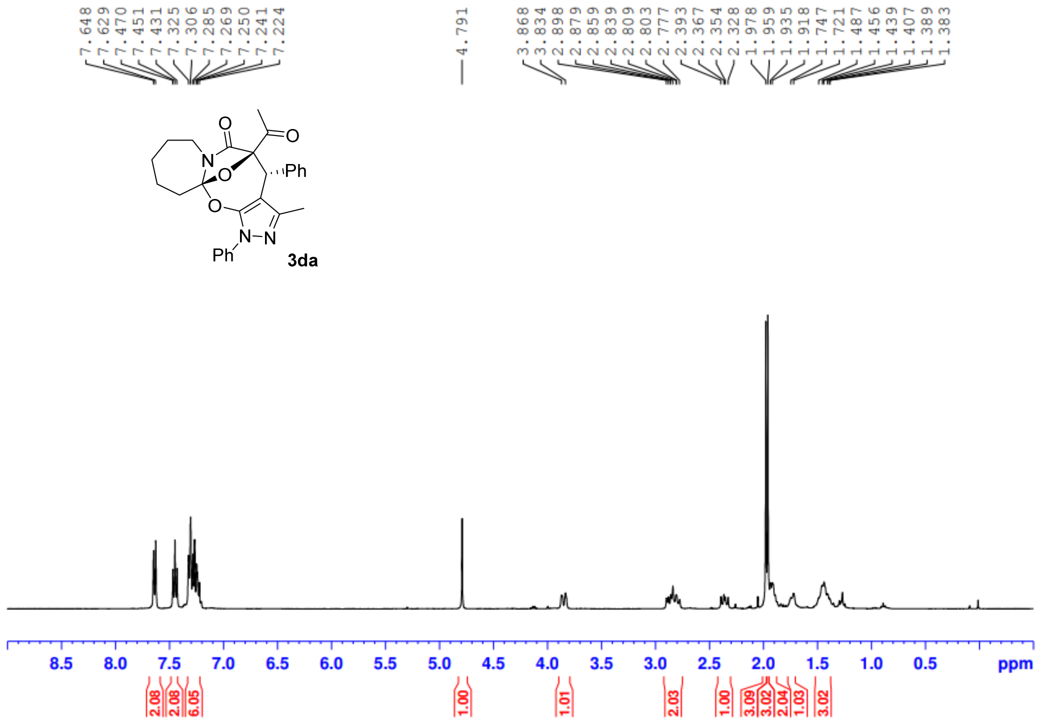

^{13}C NMR (100MHz,$CDCl_3$) of 3da

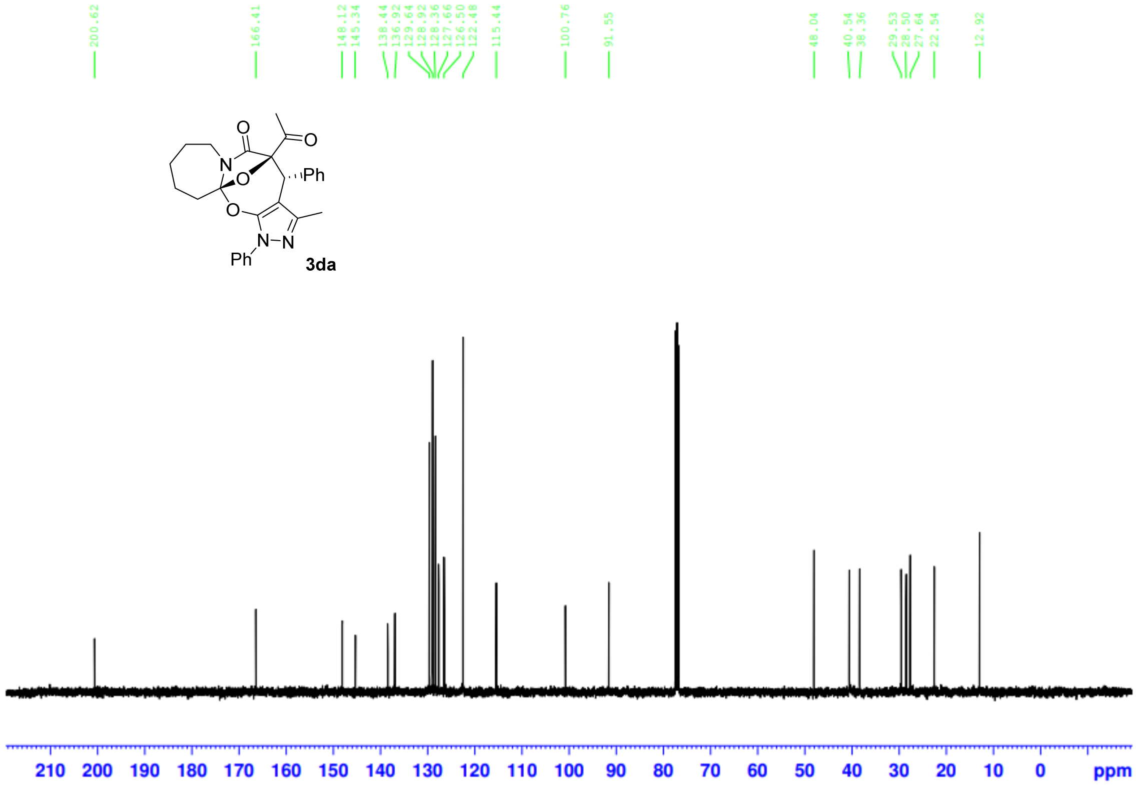

5-acetyl-3-methyl-1,4-diphenyl-4,5,8,9,10,11,12,13-octahydro-5,13a-epoxyazocino[2,1-b]pyrazolo[4,3-g][1,3]oxazocin-6(1H)-one (3ea):

^{1}H NMR (400MHz,$CDCl_3$) of 3ea

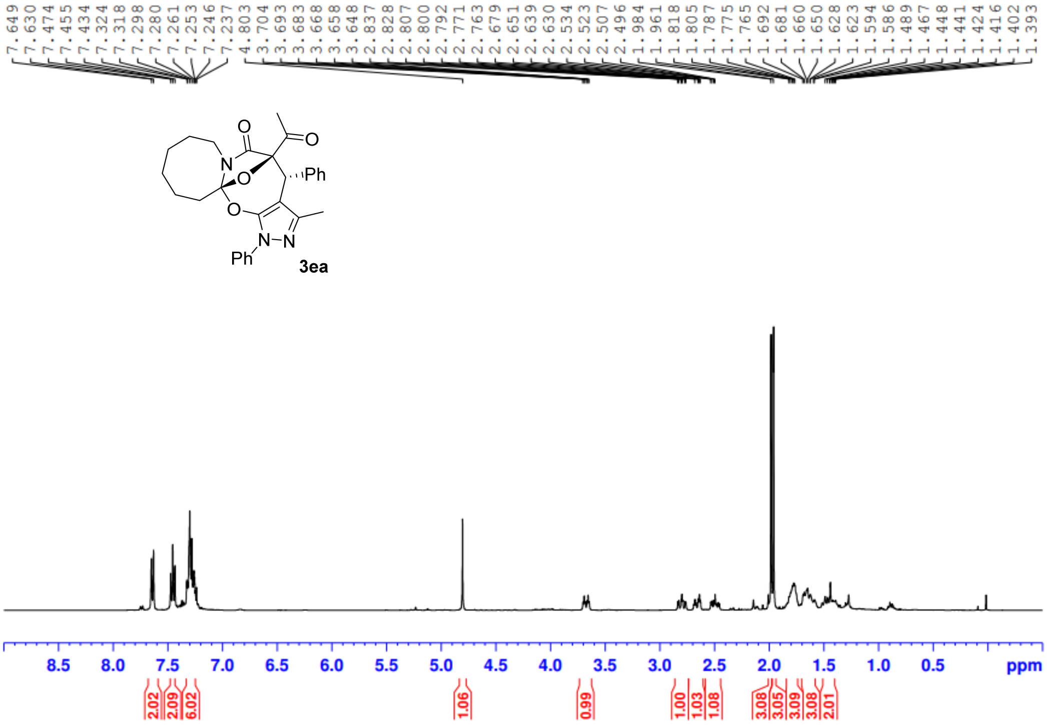

^{13}C NMR (100MHz,$CDCl_3$) of 3ea

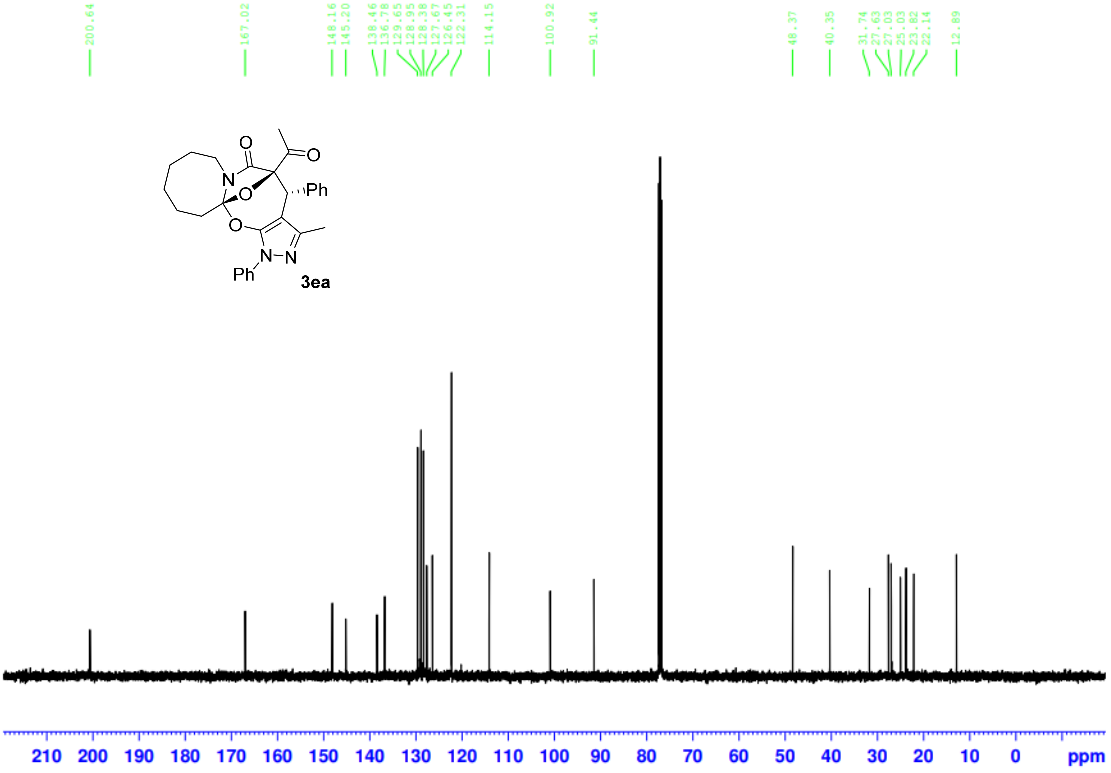

5-acetyl-3,7,8-trimethyl-1,4-diphenyl-4,5,7,8-tetrahydro-5,8-epoxypyrazolo[4,3-g][1,3]oxazocin-6(1H)-one (3fa):

^{1}H NMR (400MHz,CDCl$_3$) of 3fa

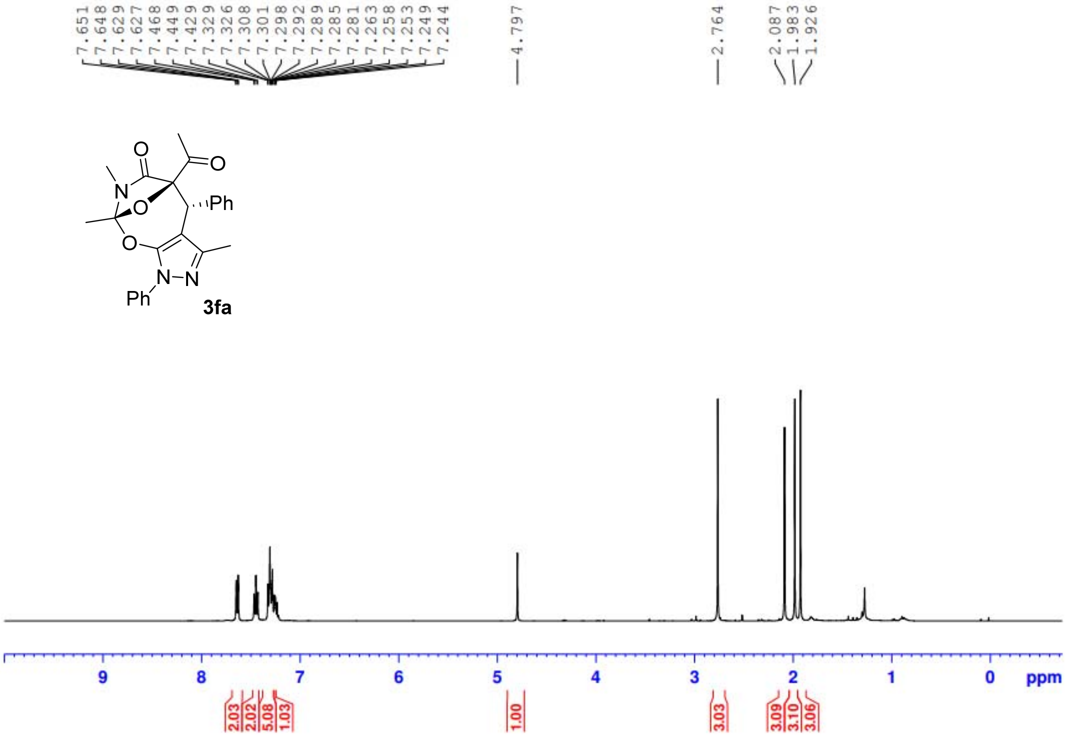

^{13}C NMR (100MHz,CDCl$_3$) of 3fa

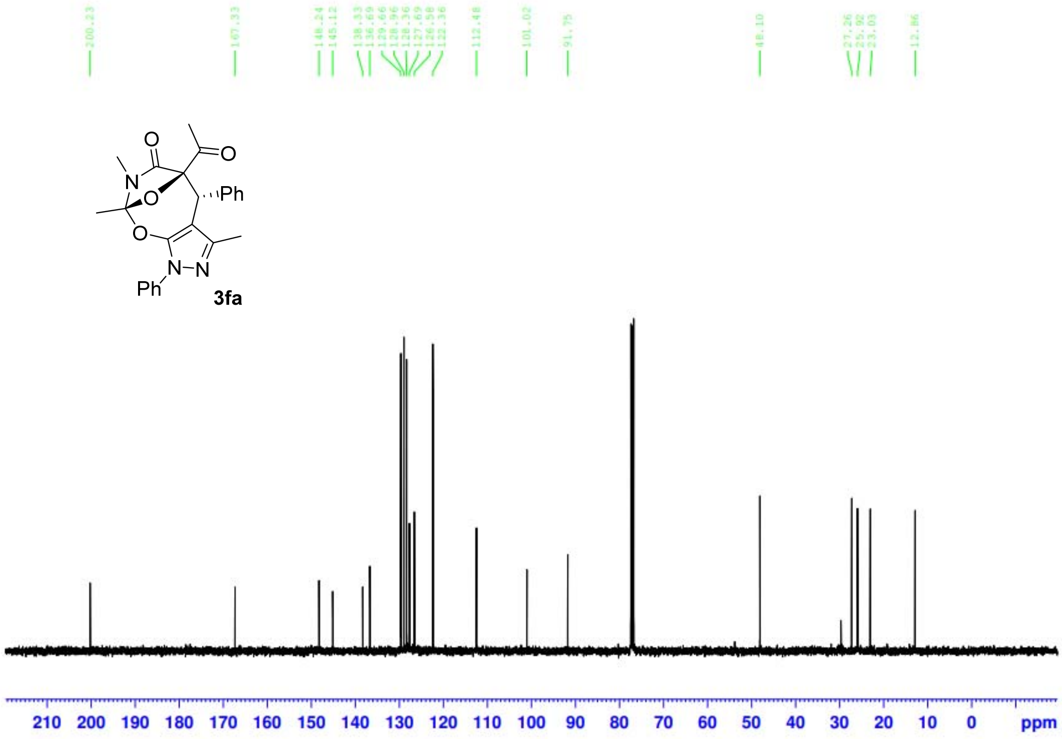

4-(4-bromophenyl)-3-methyl-1-phenyl-4,5,9,10-tetrahydro-8H-5,10a-epoxypyrazolo[4,3-g]pyrrolo[2,1-b][1,3]oxazocin-6(1H)-one (3bb):

^{1}H NMR (400MHz,$CDCl_3$) of 3bb

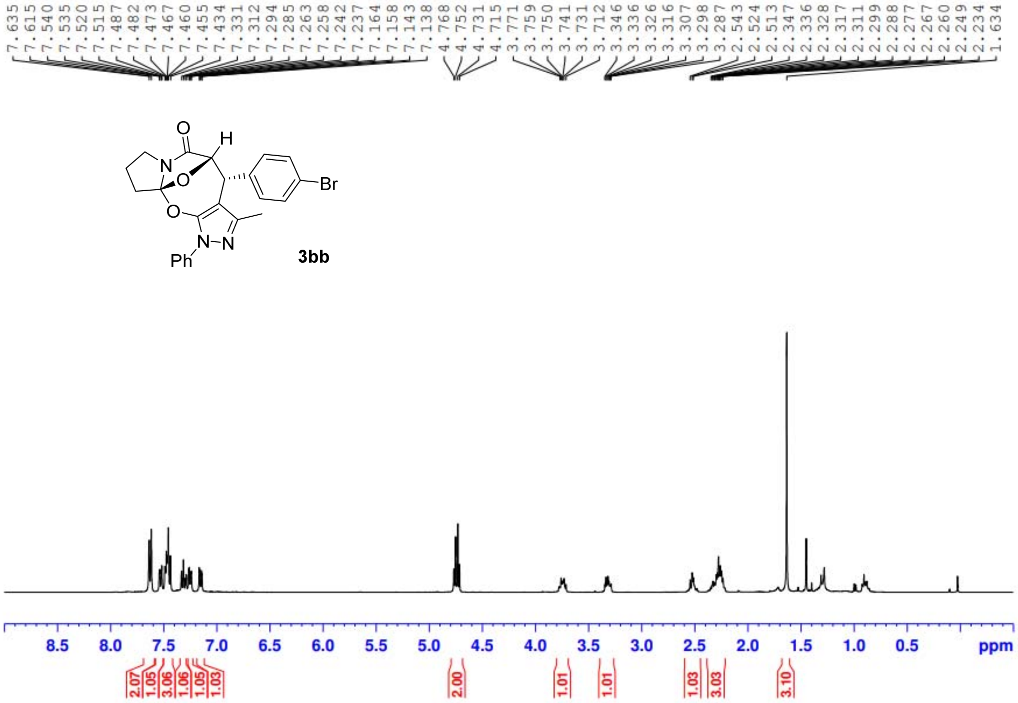

^{13}C NMR (100MHz,$CDCl_3$) of 3bb

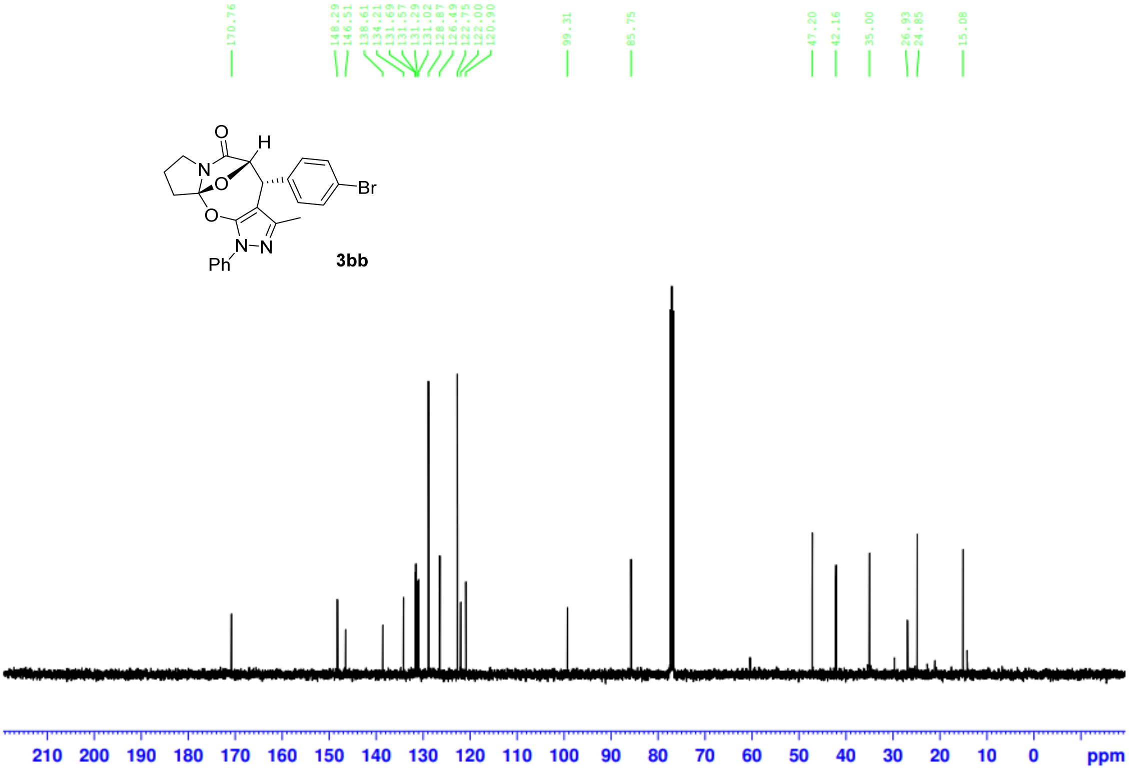

3-methyl-4-(4-nitrophenyl)-1-phenyl-4,5,9,10-tetrahydro-8H-5,10a-epoxypyrazolo[4,3-g]pyrrolo[2,1-b][1,3]oxazocin-6(1H)-one (3bc):

^{1}H NMR (400MHz,$CDCl_3$) of 3bc

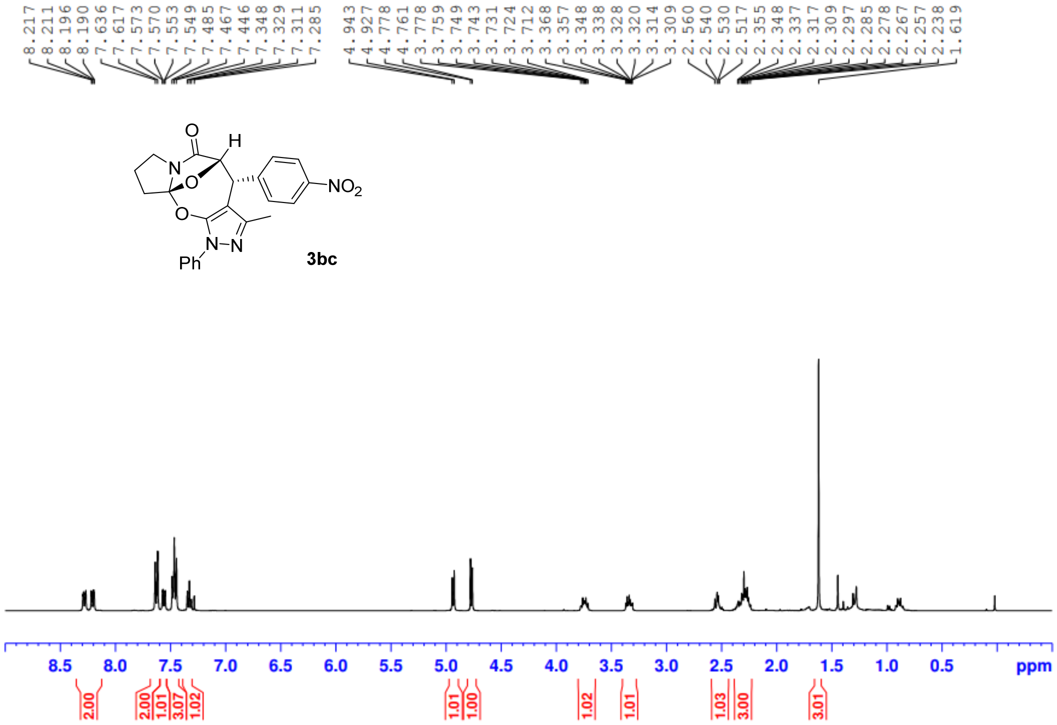

^{13}C NMR (100MHz,$CDCl_3$) of 3bc

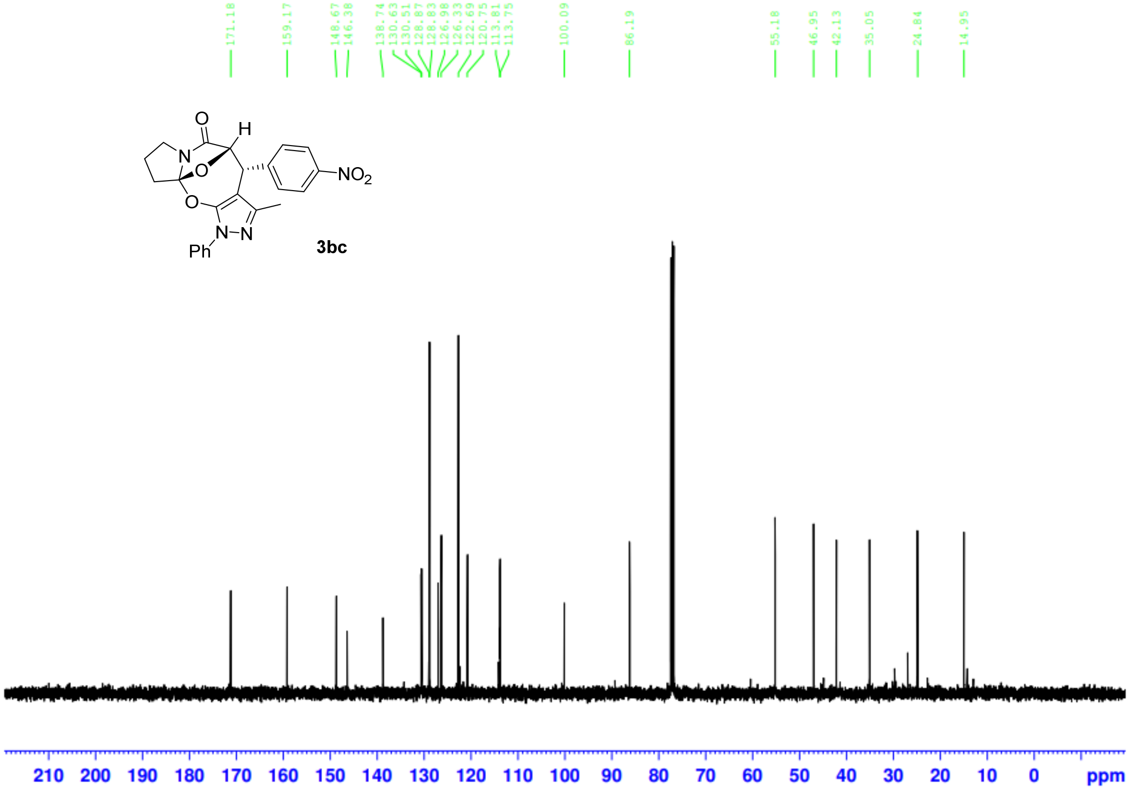

3-methyl-1-phenyl-4-(3,4,5-trimethoxyphenyl)-4,5,9,10-tetrahydro-8H-5,10a-epoxypyrazolo[4,3-g]pyrrolo[2,1-b][1,3]oxazocin-6(1H)-one (3bl):

^{1}H NMR (400MHz,CDCl$_3$) of 3bl

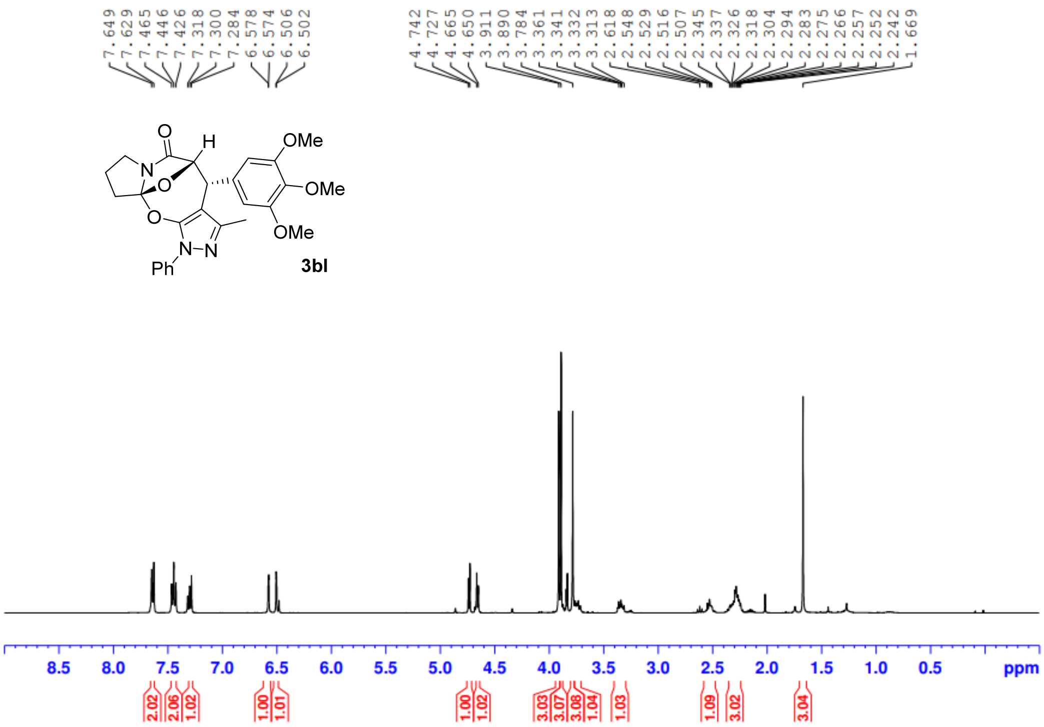

^{13}C NMR (100MHz,CDCl$_3$) of 3bl

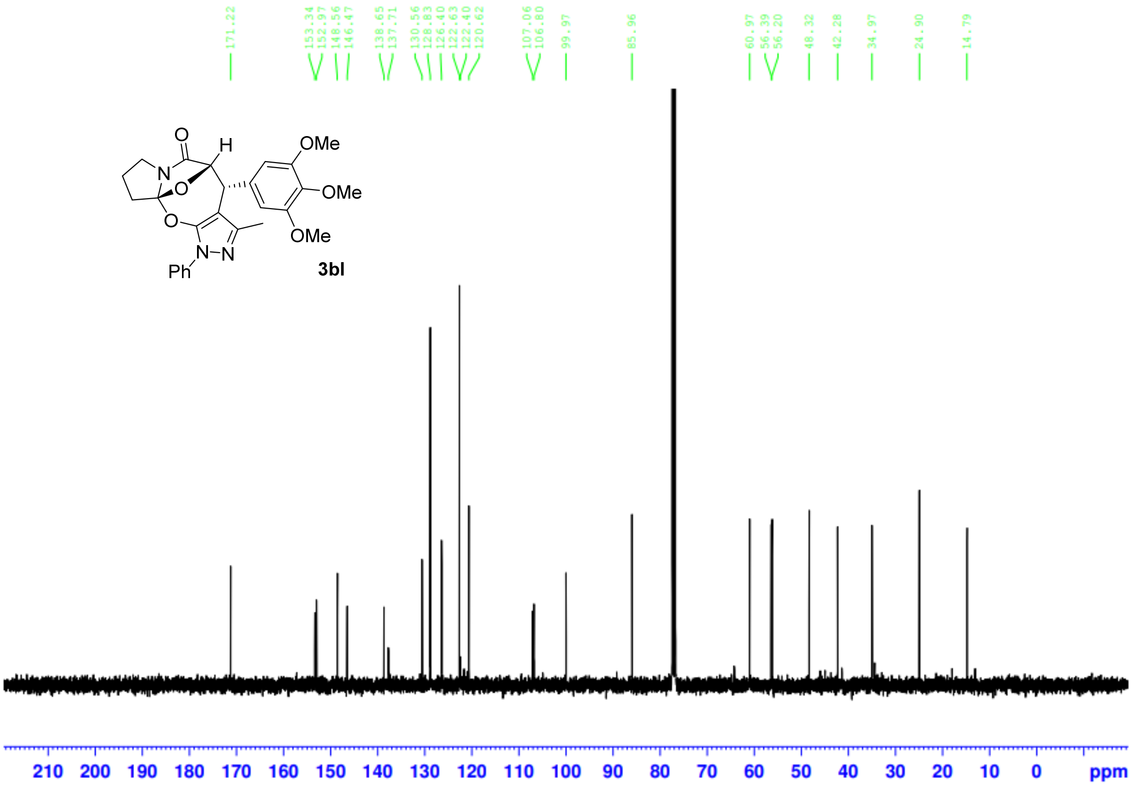

3-methyl-1-phenyl-4-(4-(trifluoromethyl)phenyl)-4,5,9,10-tetrahydro-8H-5,10a-epoxypyrazolo[4,3-g]pyrrolo[2,1-b][1,3]oxazocin-6(1H)-one (3bm):

^{1}H NMR (400MHz,$CDCl_3$) of 3bm

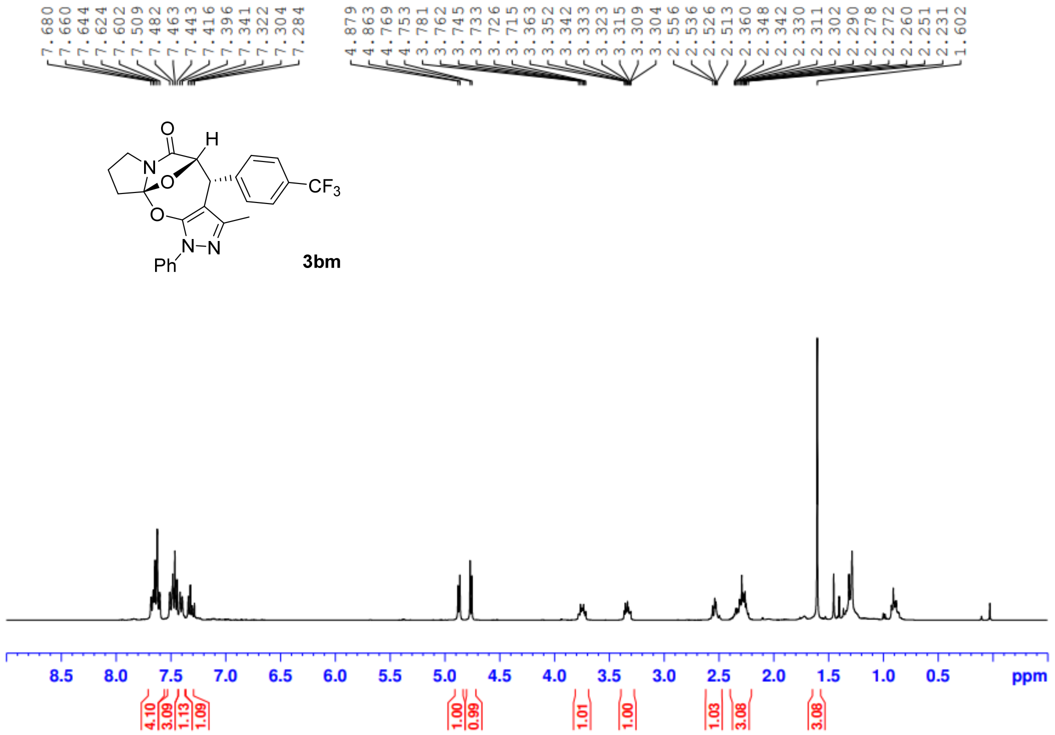

^{13}C NMR (100MHz,$CDCl_3$) of 3bm

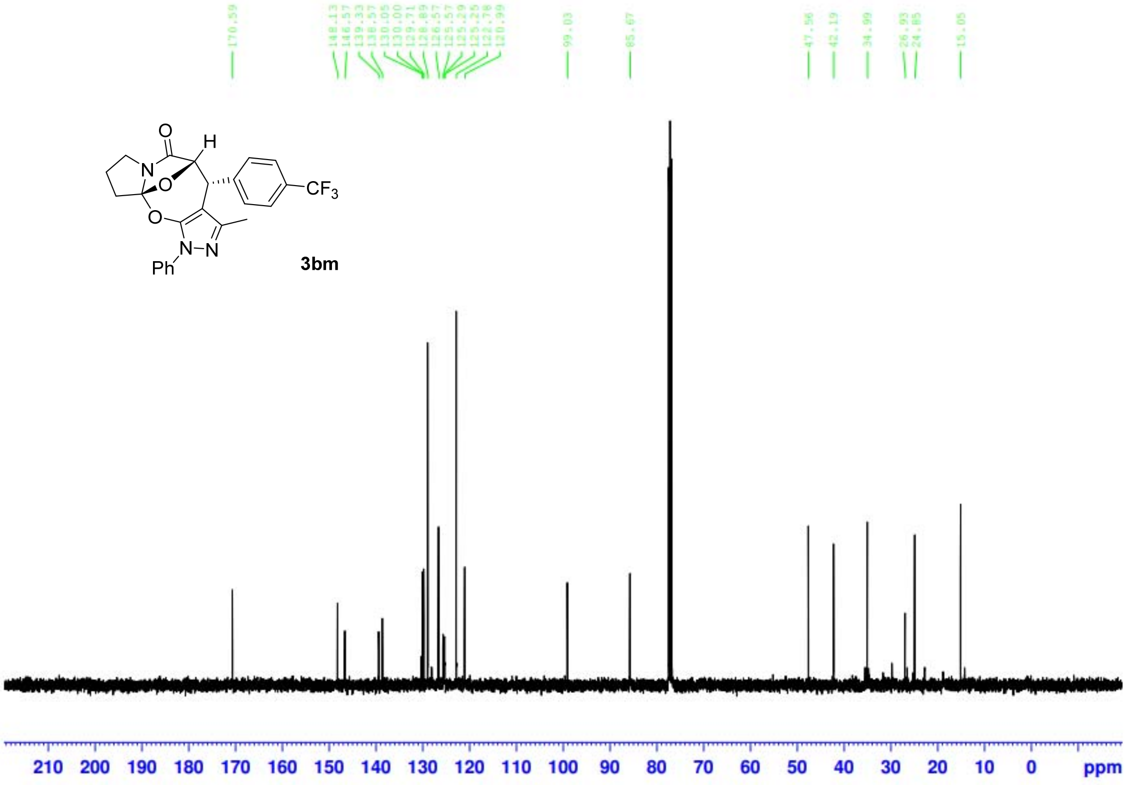

^{19}F NMR (400MHz,$CDCl_3$) of 3bm

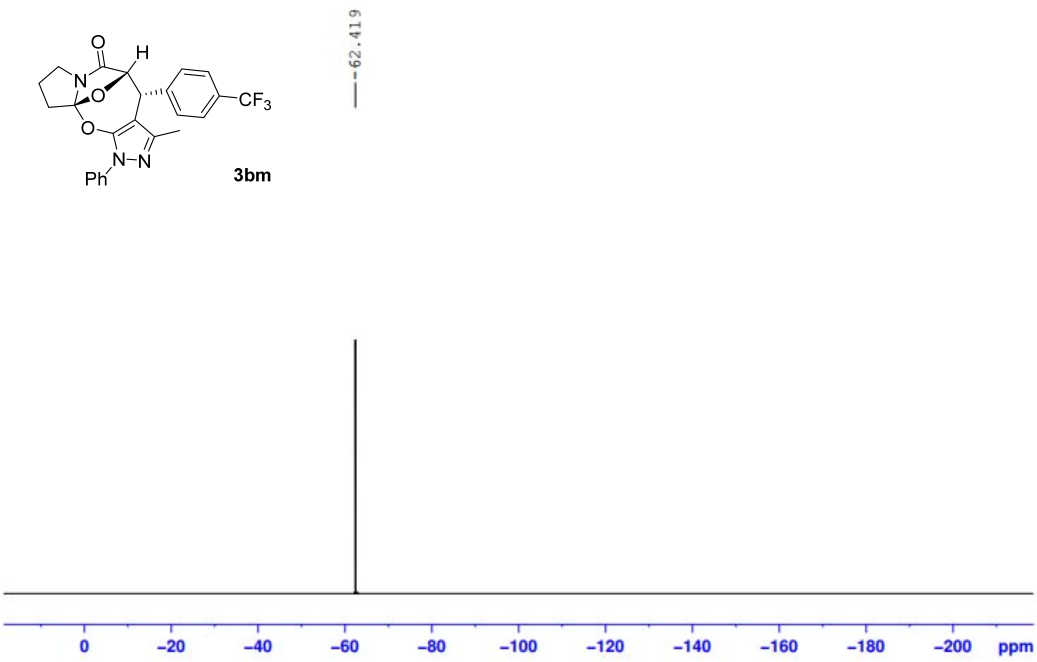

4-(2-methoxyphenyl)-3-methyl-1-phenyl-4,5,9,10-tetrahydro-8H-5,10a-epoxypyrazolo[4,3-g] pyrrolo[2,1-b][1,3]oxazocin-6(1H)-one (3bn):

^{1}H NMR (400MHz,$CDCl_3$) of 3bn

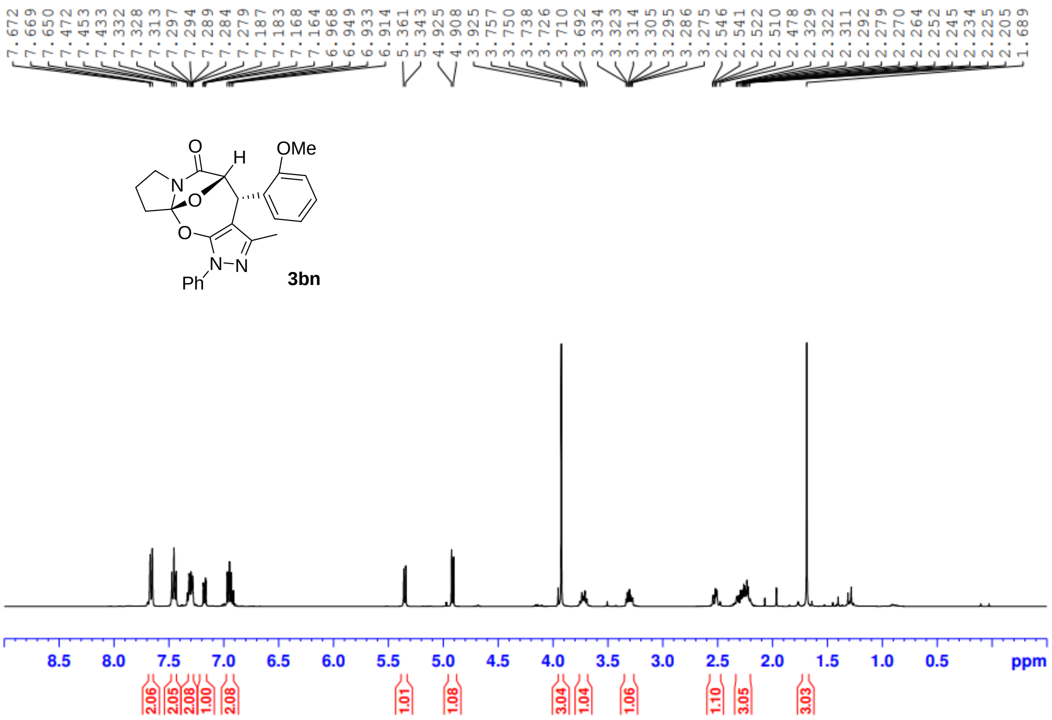

^{13}C NMR (100MHz,$CDCl_3$) of 3bn

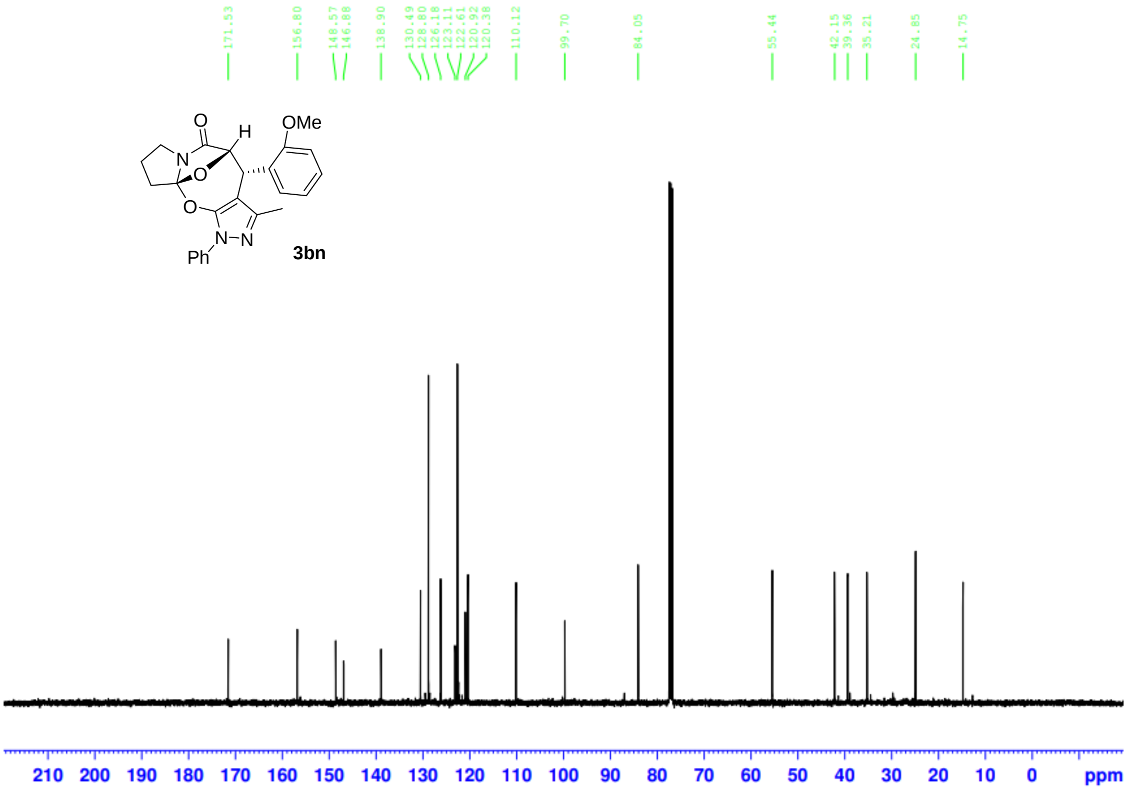

3,4,4-trimethyl-1-phenyl-4,5,9,10-tetrahydro-8H-5,10a-epoxypyrazolo[4,3-g]pyrrolo[2,1-b][1,3]oxazocin-6(1H)-one (3bo):

^{1}H NMR (400MHz,CDCl$_3$) of 3bo

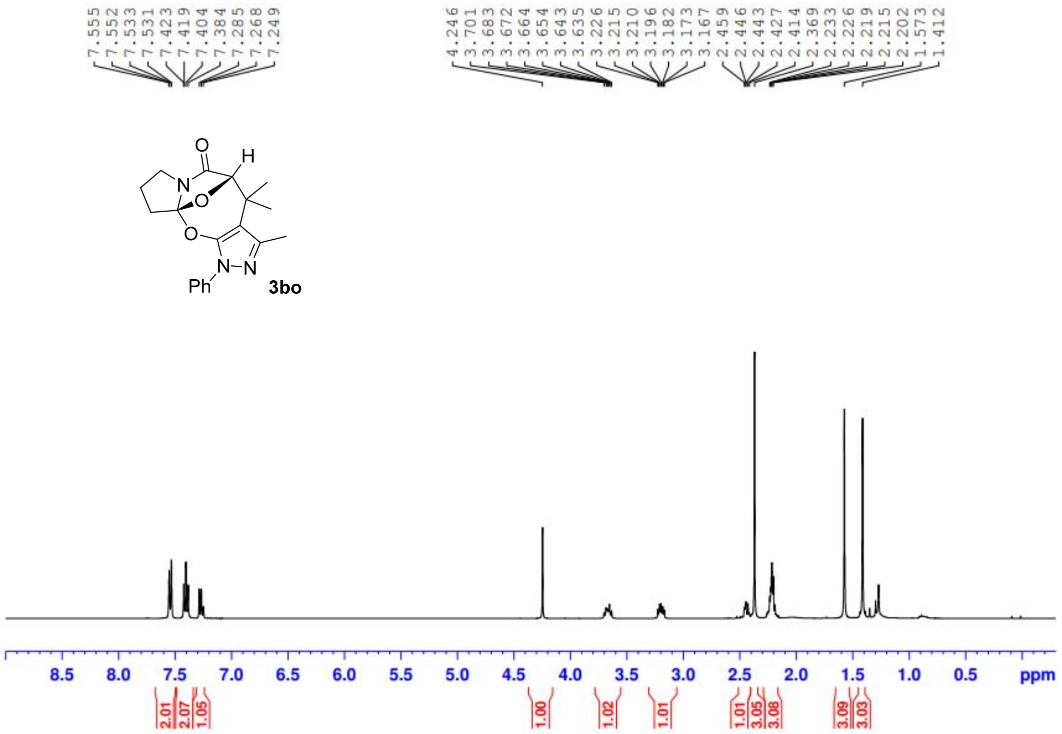

^{13}C NMR (100MHz,CDCl$_3$) of 3bo

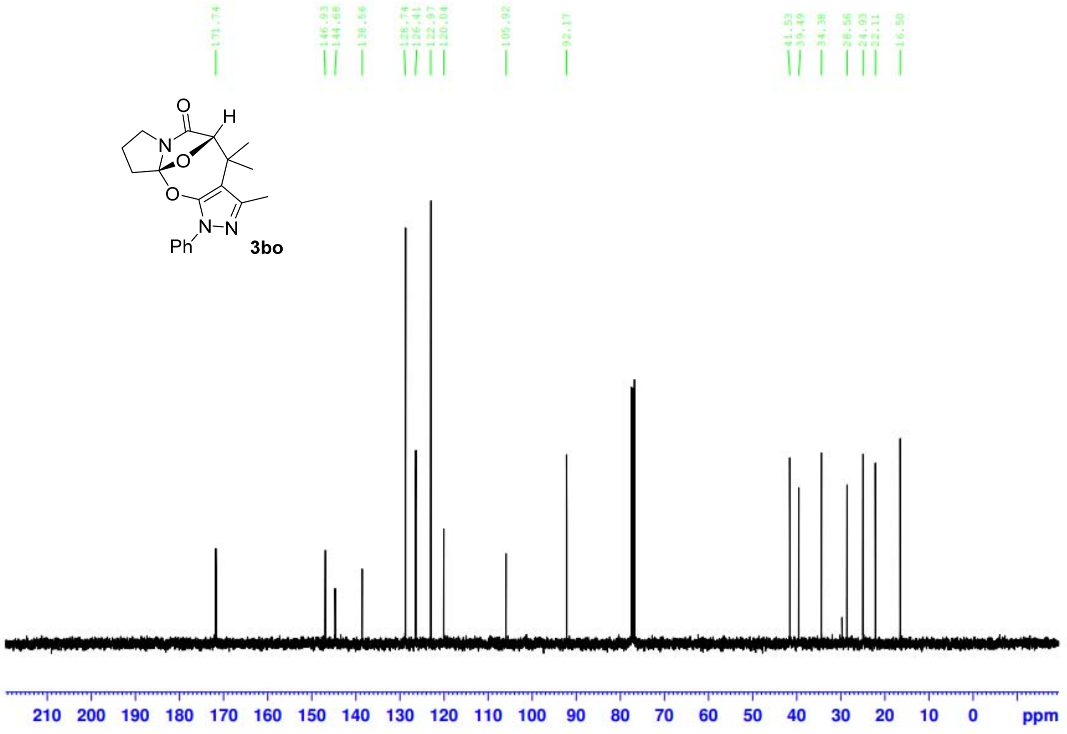

5-acetyl-3-methyl-4-(4-nitrophenyl)-1-phenyl-4,5,8,9,10,11-hexahydro-5,11a-epoxypyrazolo[4,3-g]pyrido[2,1-b][1,3]oxazocin-6(1H)-one (3cc):

^{1}H NMR (400MHz,$CDCl_3$) of 3cc

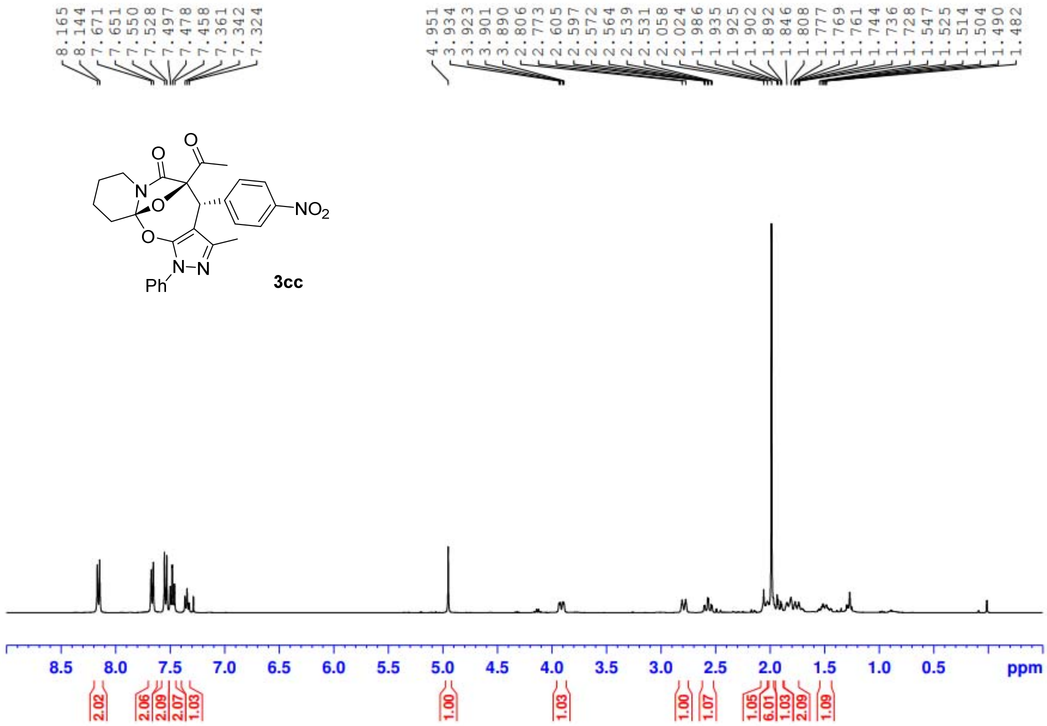

^{13}C NMR (100MHz,$CDCl_3$) of 3cc

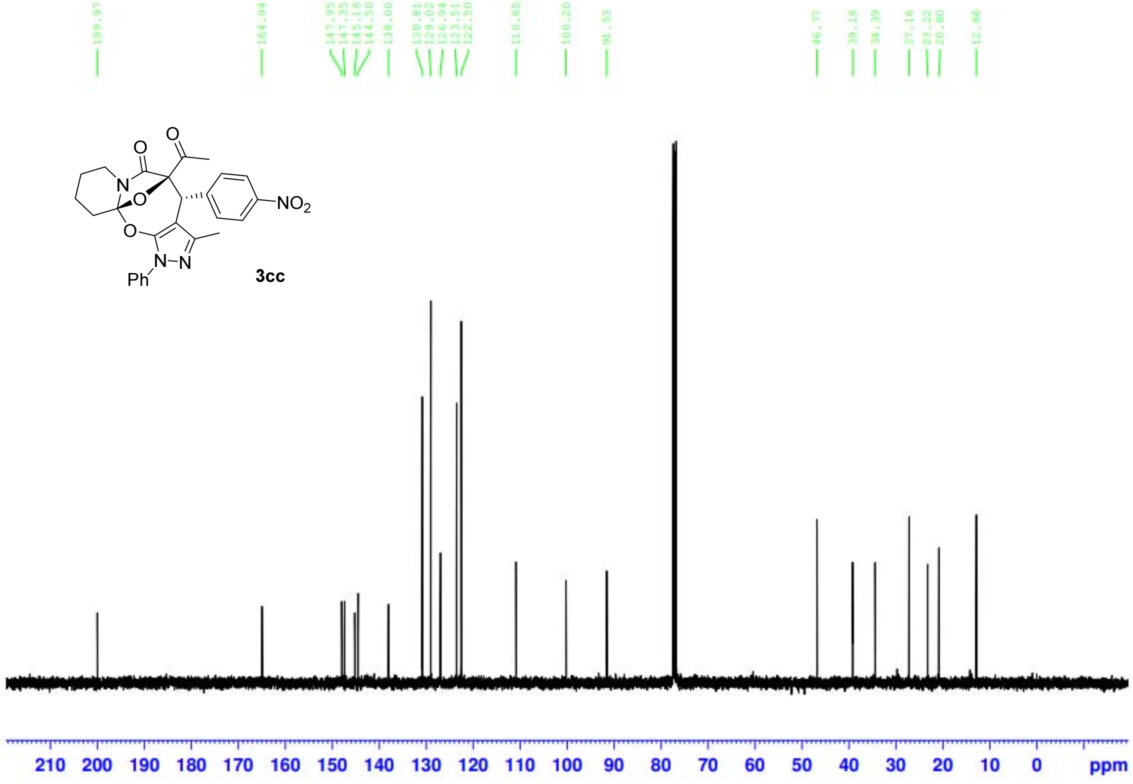

5-acetyl-4-(4-methoxyphenyl)-3-methyl-1-phenyl-4,5,8,9,10,11-hexahydro-5,11a-epoxypyrazolo[4,3-g]pyrido[2,1-b][1,3]oxazocin-6(1H)-one (3cd):

^{1}H NMR (400MHz,CDCl$_3$) of 3cd

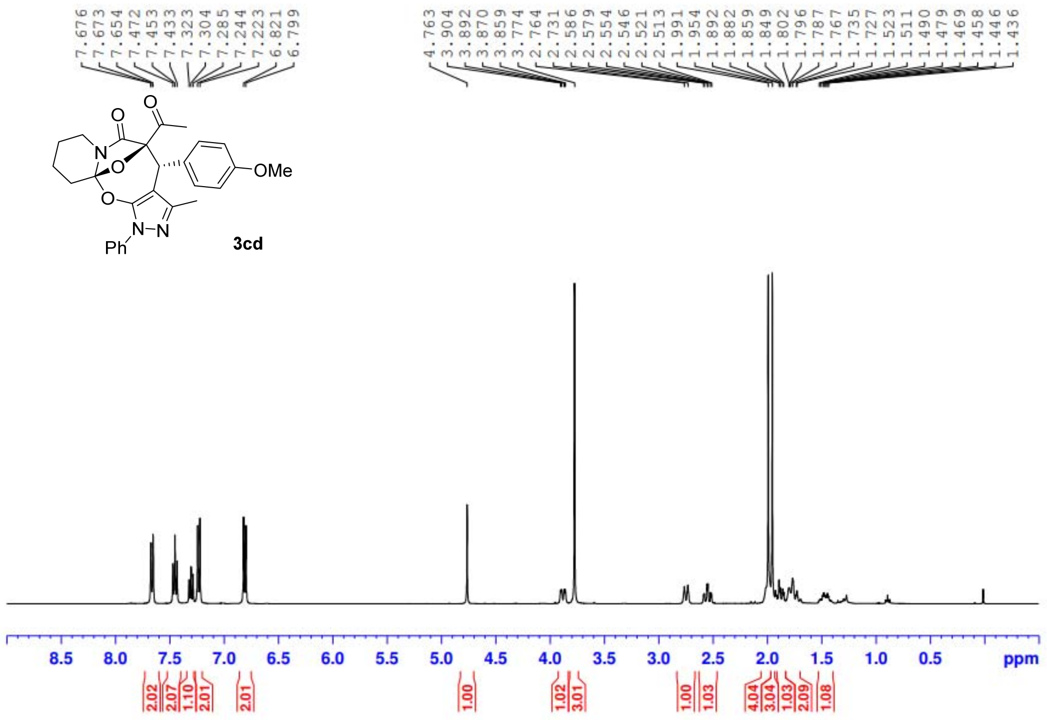

^{13}C NMR (100MHz,CDCl$_3$) of 3cd

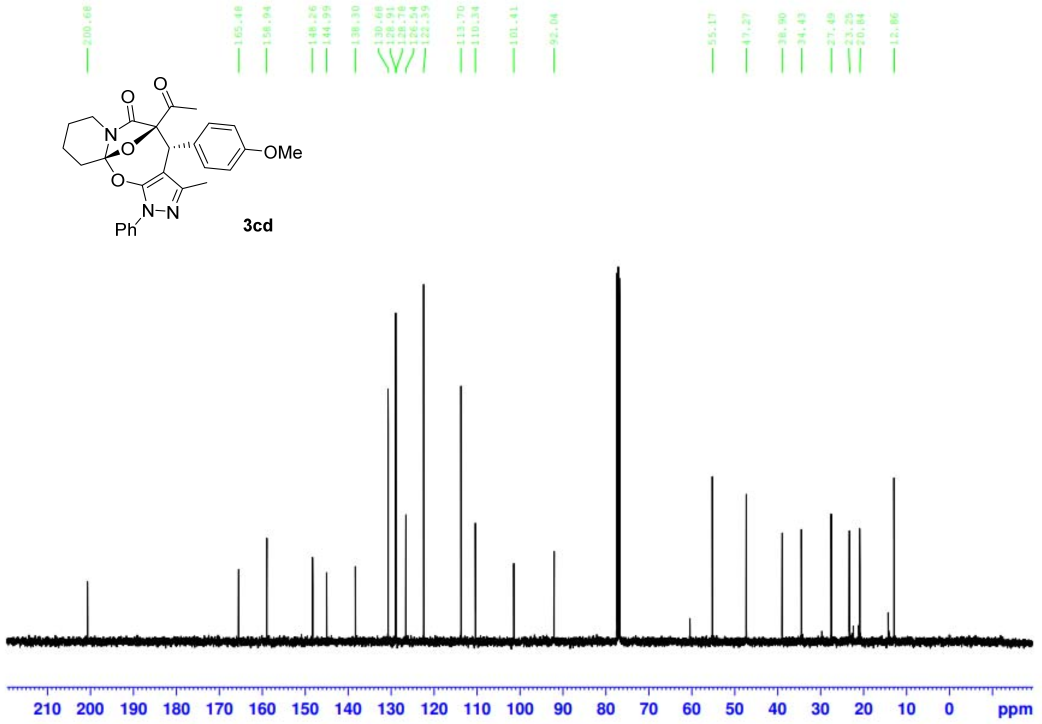

5-acetyl-3-methyl-4-(naphthalen-2-yl)-1-phenyl-4,5,8,9,10,11-hexahydro-5,11a-epoxypyrazolo[4,3-g]pyrido[2,1-b][1,3]oxazocin-6(1H)-one (3ce):

^{1}H NMR (400MHz,$CDCl_3$) of 3ce

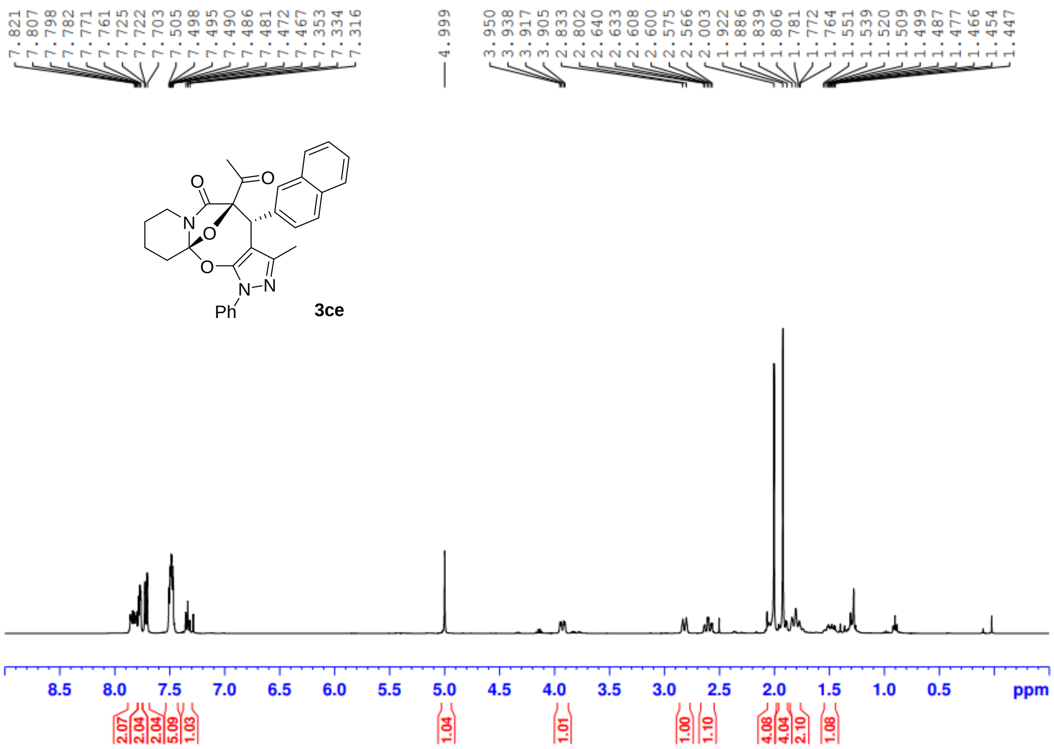

^{13}C NMR (100MHz,$CDCl_3$) of 3ce

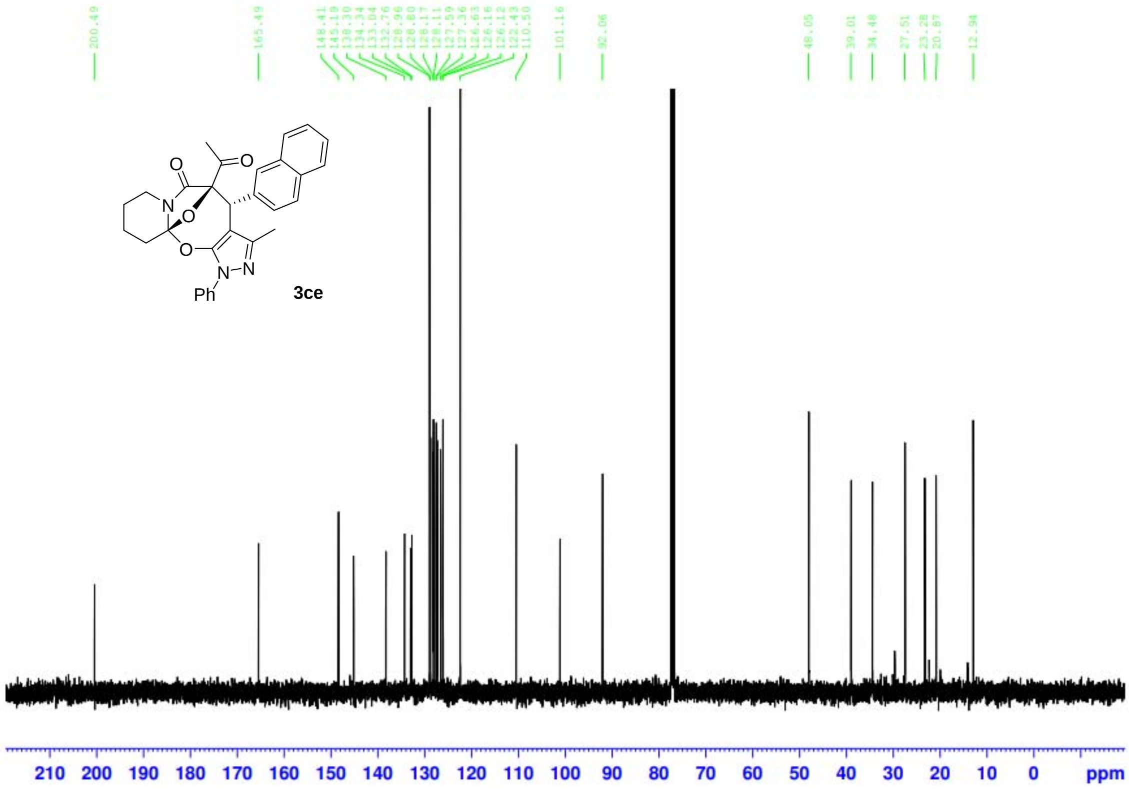

5-acetyl-1-(4-chlorophenyl)-3-methyl-4-phenyl-4,5,8,9,10,11-hexahydro-5,11a-epoxypyrazolo [4,3-g]pyrido[2,1-b][1,3]oxazocin-6(1H)-one (3cf):

^{1}H NMR (400MHz,$CDCl_3$) of 3cf

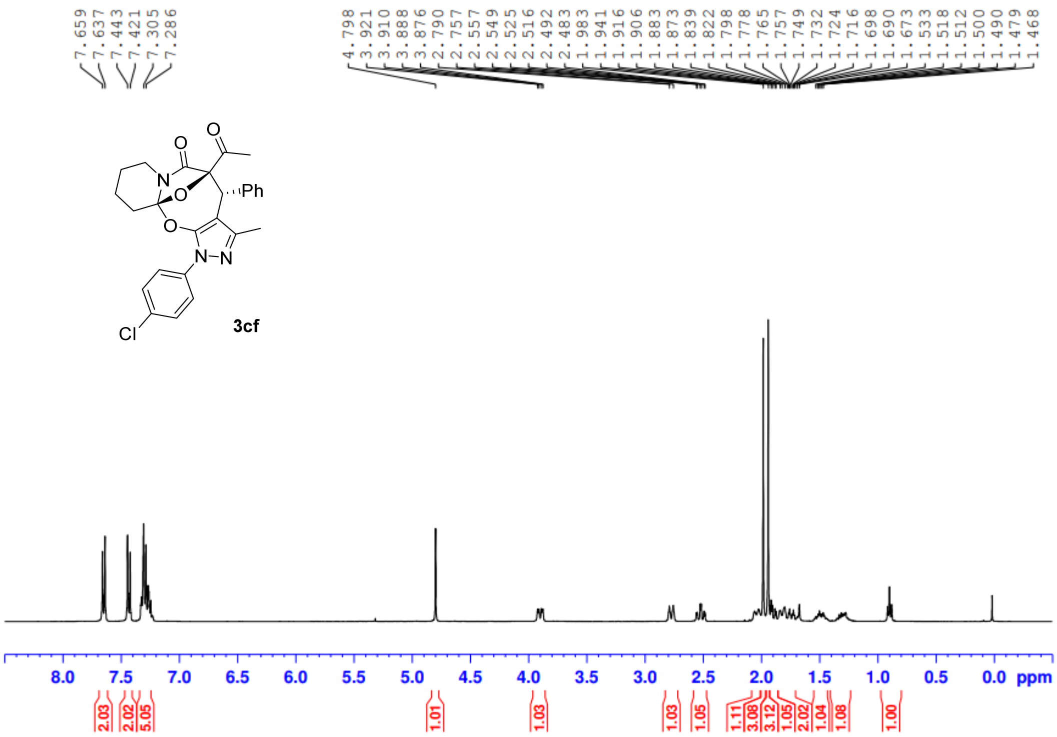

^{13}C NMR (100MHz,$CDCl_3$) of 3cf

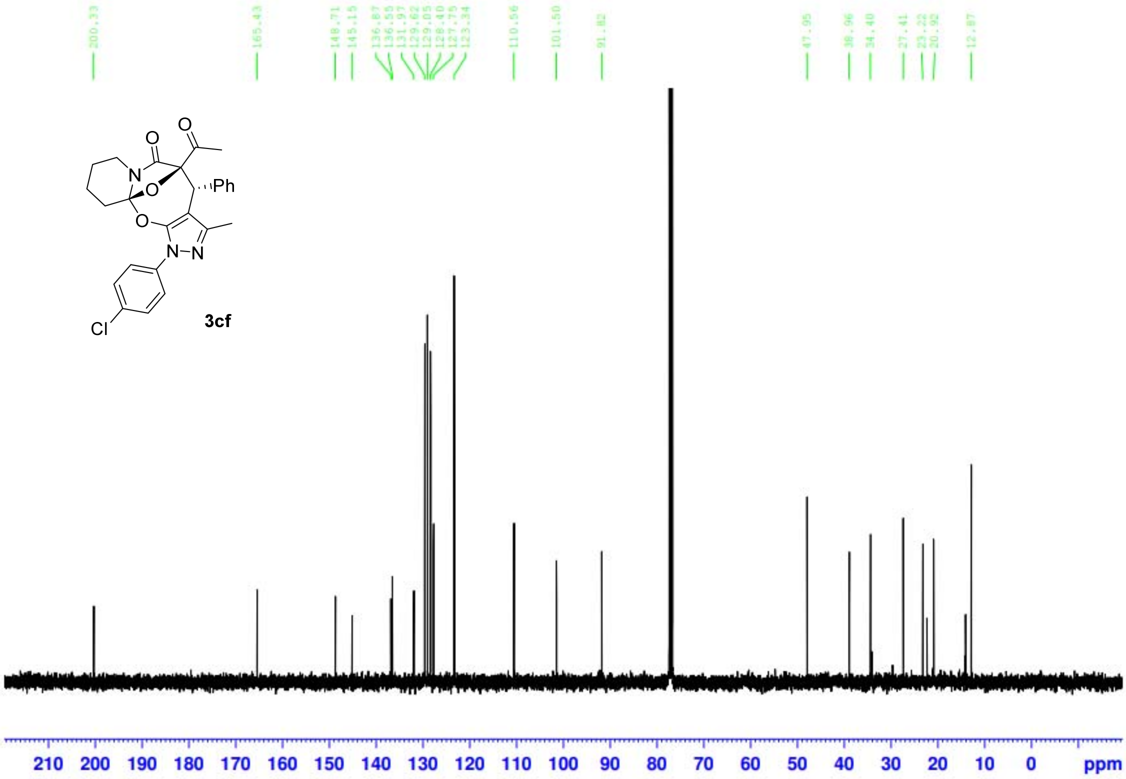

5-acetyl-3-methyl-4-phenyl-1-(p-tolyl)-4,5,8,9,10,11-hexahydro-5,11a-epoxypyrazolo[4,3-g]pyrido[2,1-b][1,3]oxazocin-6(1H)-one (3cg):

^{1}H NMR (400MHz,$CDCl_3$) of 3cg

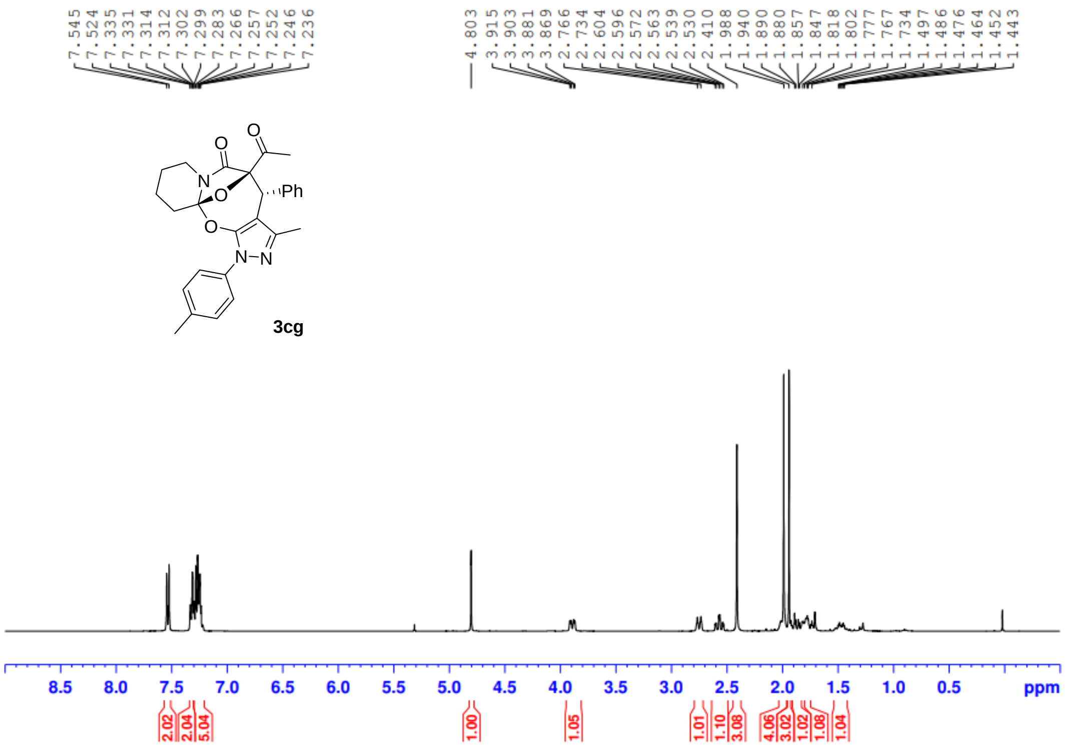

^{13}C NMR (100MHz,$CDCl_3$) of 3cg

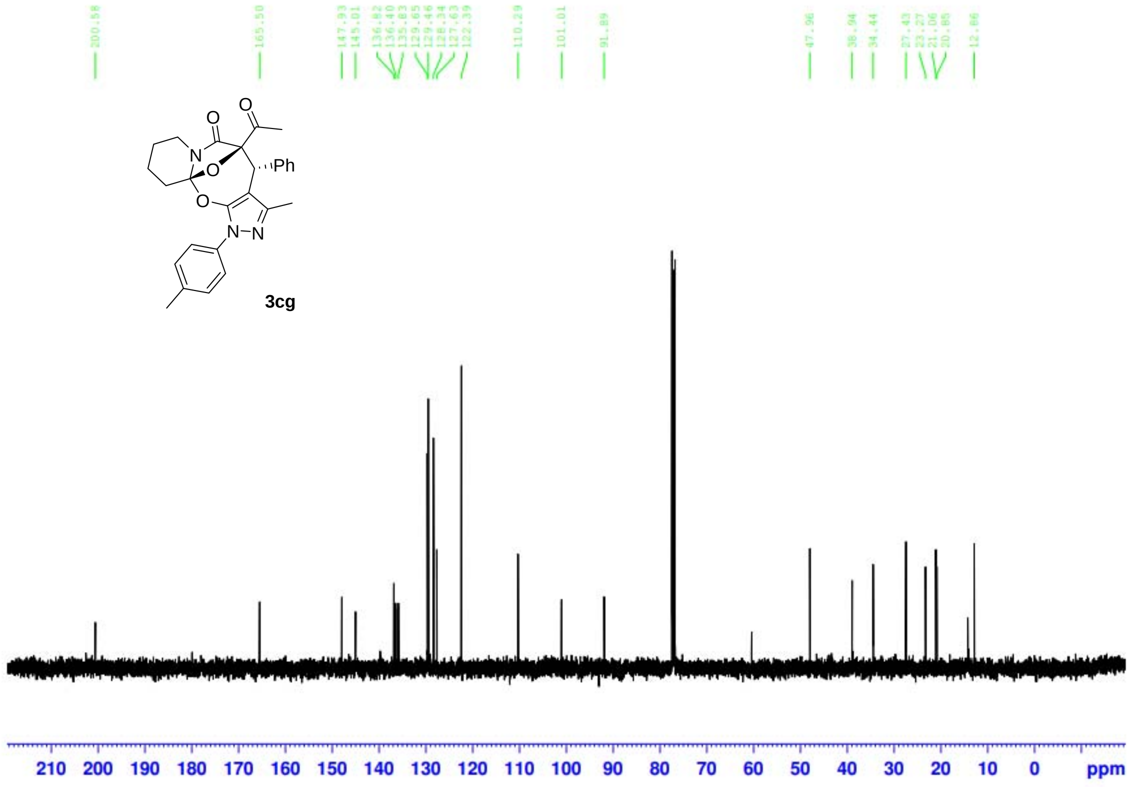

5-acetyl-3-methyl-1-phenyl-4-(3,4,5-trimethoxyphenyl)-4,5,8,9,10,11-hexahydro-5,11a-epoxy pyrazolo[4,3-g]pyrido[2,1-b][1,3]oxazocin-6(1H)-one (3cl):

^{1}H NMR (400MHz,CDCl$_3$) of 3cl

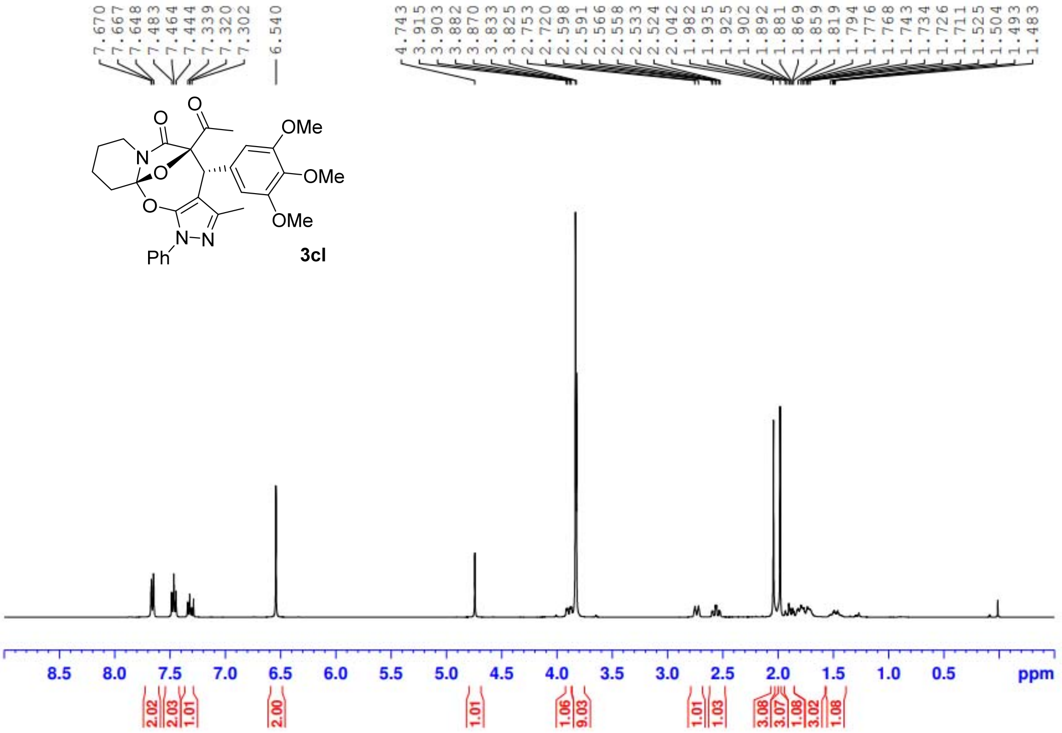

^{13}C NMR (100MHz,CDCl$_3$) of 3cl

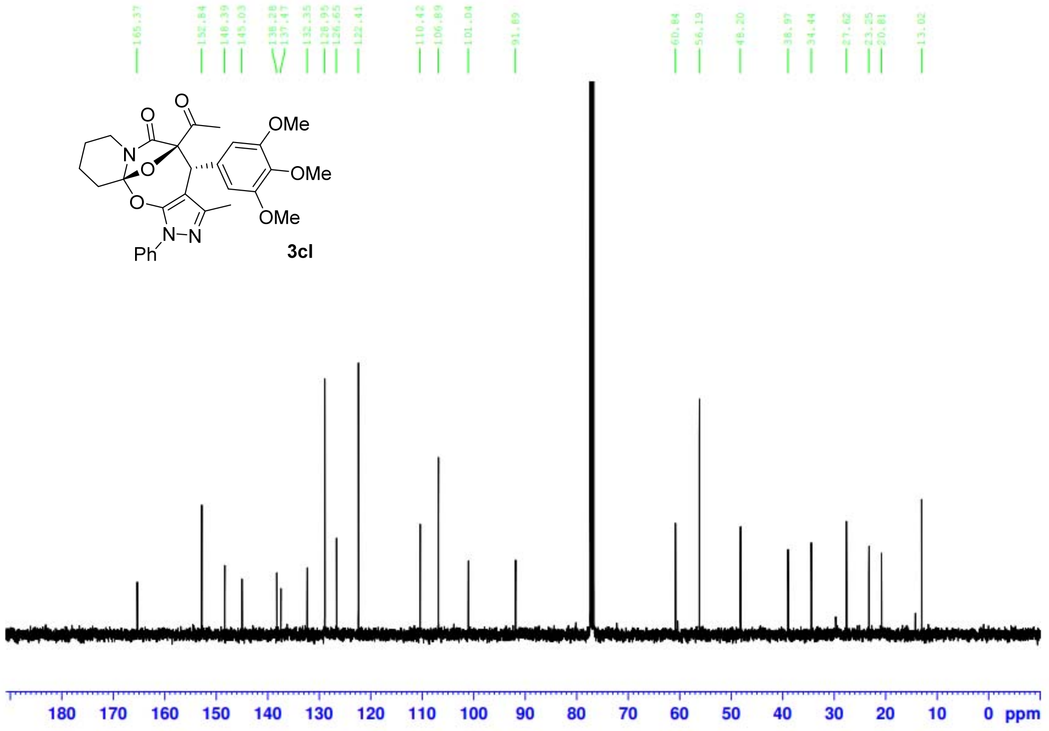

5-acetyl-4-(2-methoxyphenyl)-3-methyl-1-phenyl-4,5,8,9,10,11-hexahydro-5,11a-epoxypyrazolo[4,3-g]pyrido[2,1-b][1,3]oxazocin-6(1H)-one (3cn):

^{1}H NMR (400MHz,$CDCl_3$) of 3cn

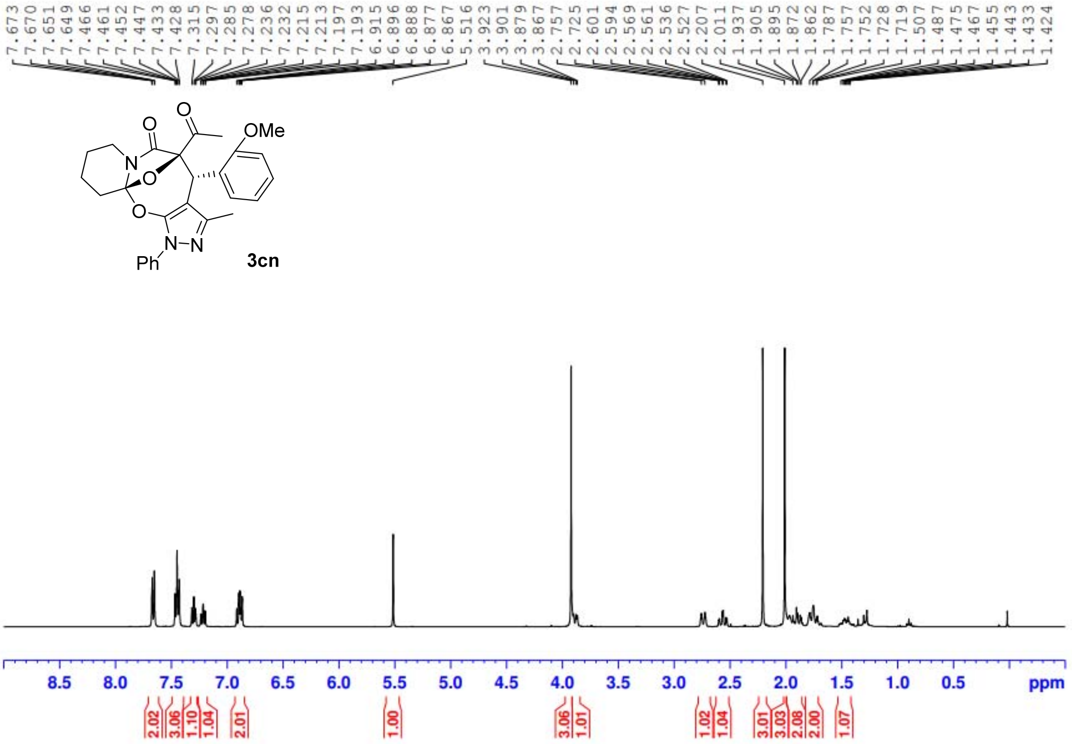

^{13}C NMR (100MHz,$CDCl_3$) of 3cn

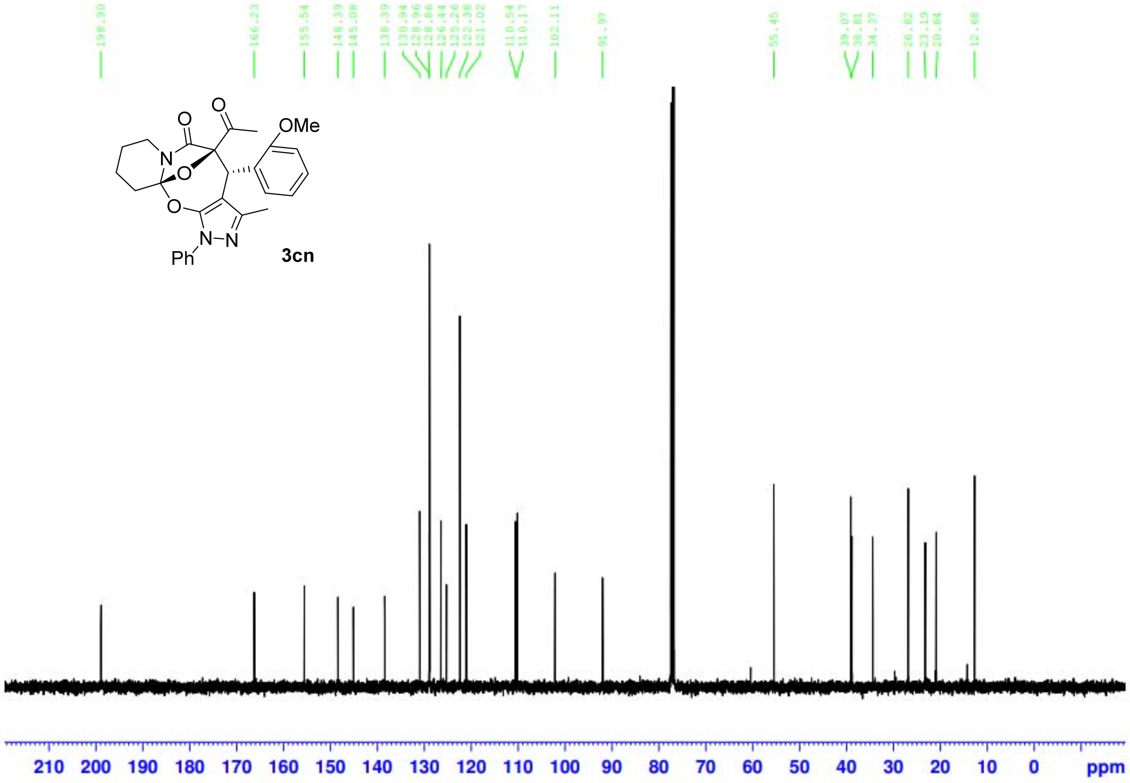

5-acetyl-3-methyl-1-phenyl-4-(thiophen-2-yl)-4,5,8,9,10,11-hexahydro-5,11a-epoxypyrazolo[4,3-g]pyrido[2,1-b][1,3]oxazocin-6(1H)-one (3cp):

^{1}H NMR (400MHz,$CDCl_3$) of 3cp

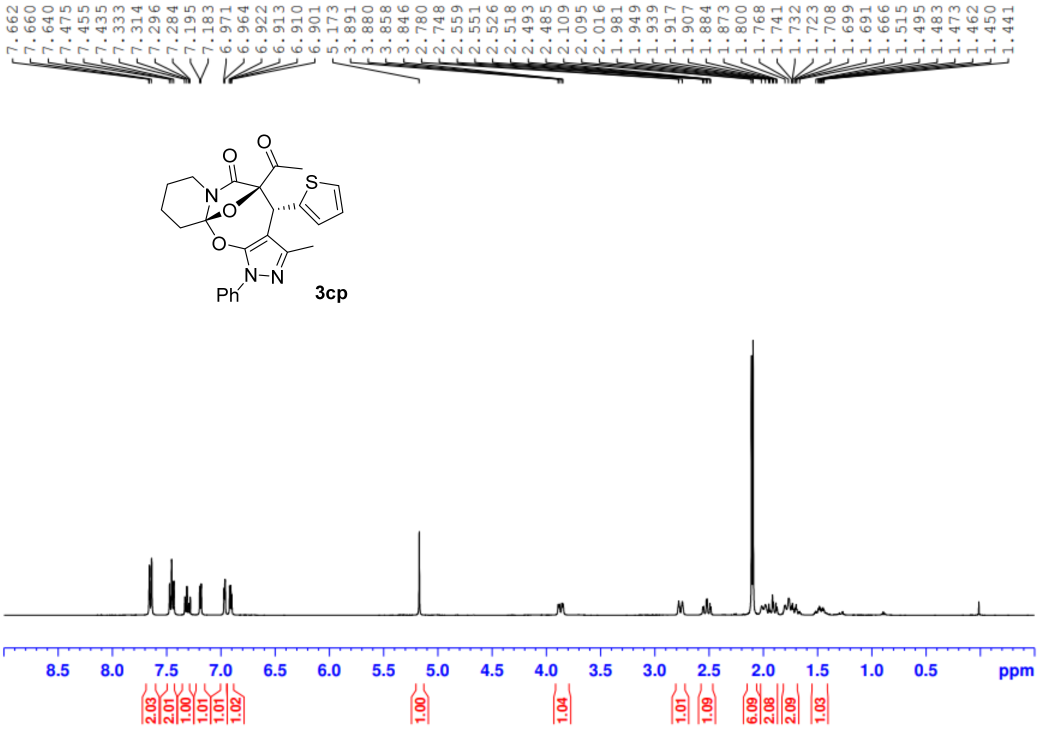

^{13}C NMR (100MHz,$CDCl_3$) of 3cp

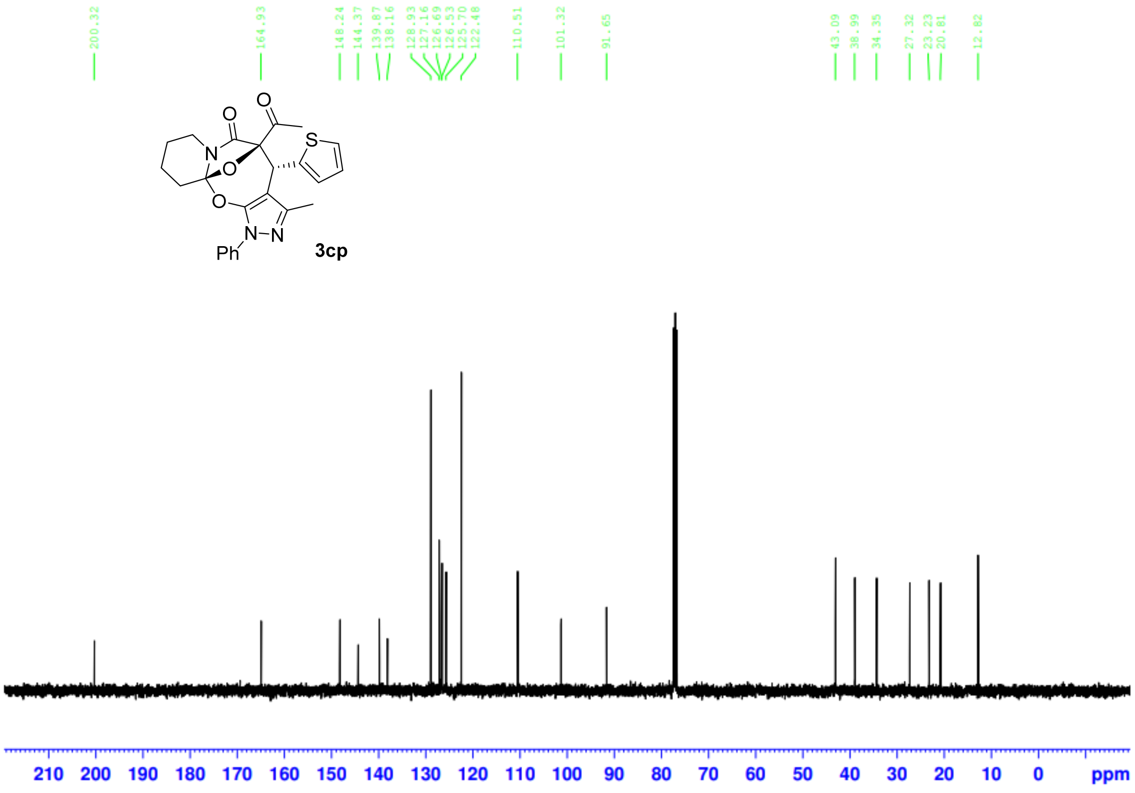

5-acetyl-4-(4-bromophenyl)-3-methyl-1-phenyl-4,5,9,10,11,12-hexahydro-8H-5,12a-epoxyazepino[2,1-b]pyrazolo[4,3-g][1,3]oxazocin-6(1H)-one (3db):

^{1}H NMR (400MHz,$CDCl_3$) of 3db

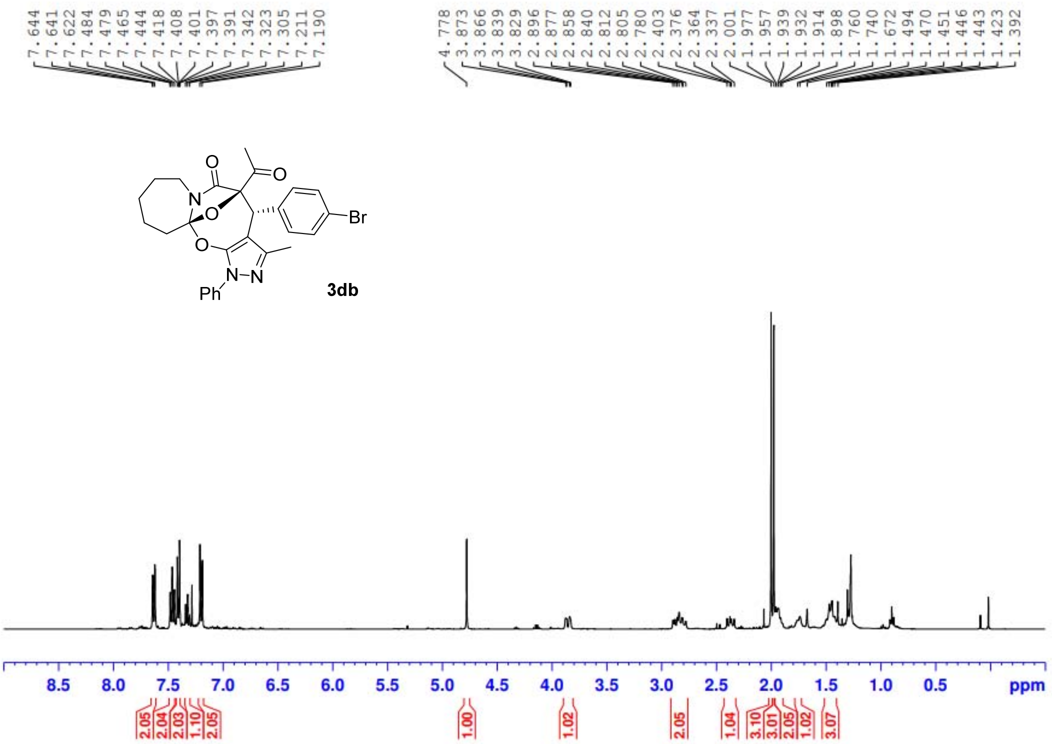

^{13}C NMR (100MHz,$CDCl_3$) of 3db

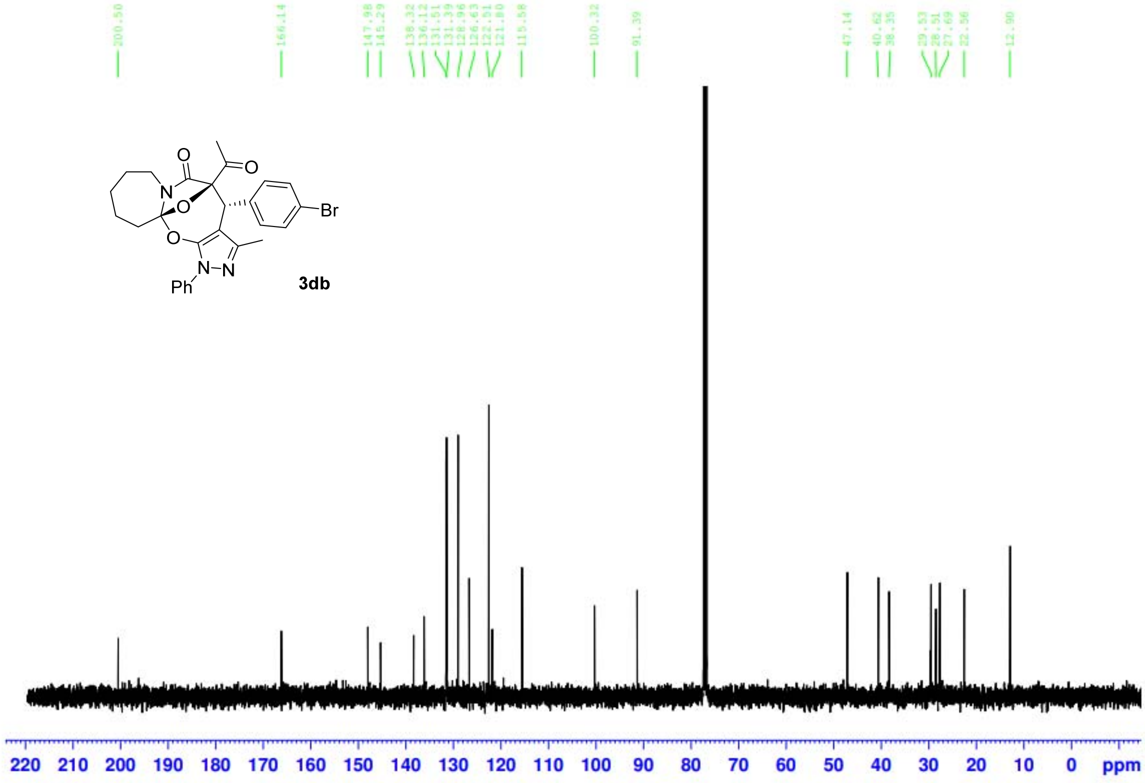

5-acetyl-4-(4-methoxyphenyl)-3-methyl-1-phenyl-4,5,9,10,11,12-hexahydro-8H-5,12a-epoxyazepino[2,1-b]pyrazolo[4,3-g][1,3]oxazocin-6(1H)-one (3dd):

^{1}H NMR (400MHz,$CDCl_3$) of 3dd

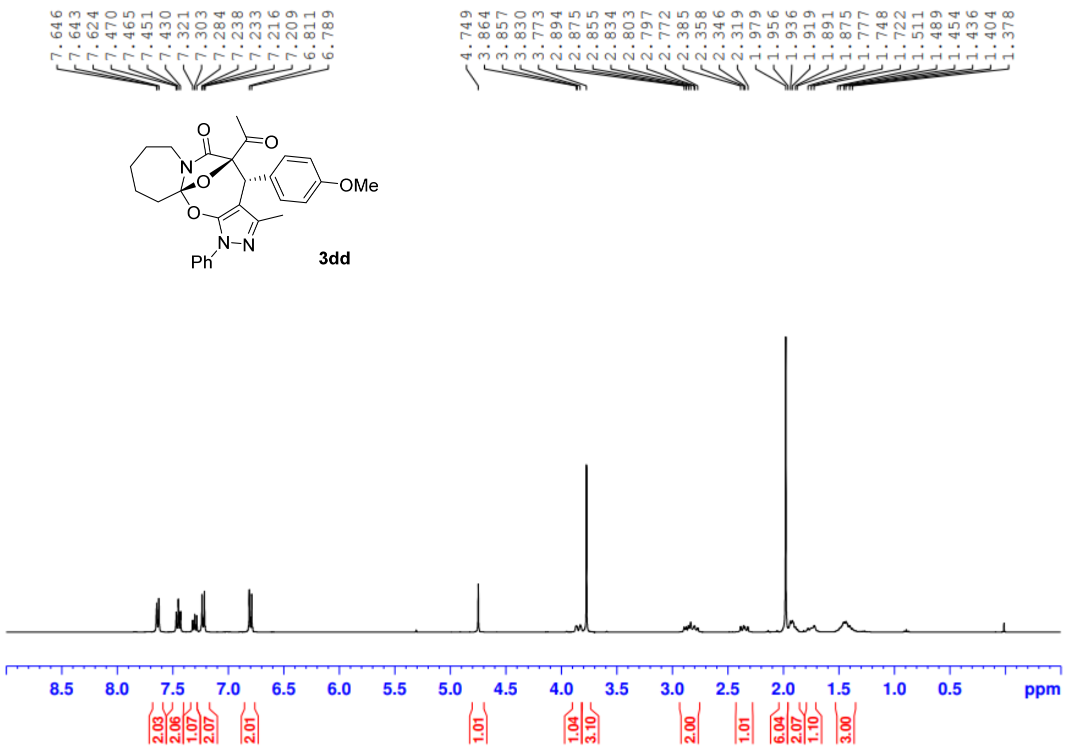

^{13}C NMR (100MHz,$CDCl_3$) of 3dd

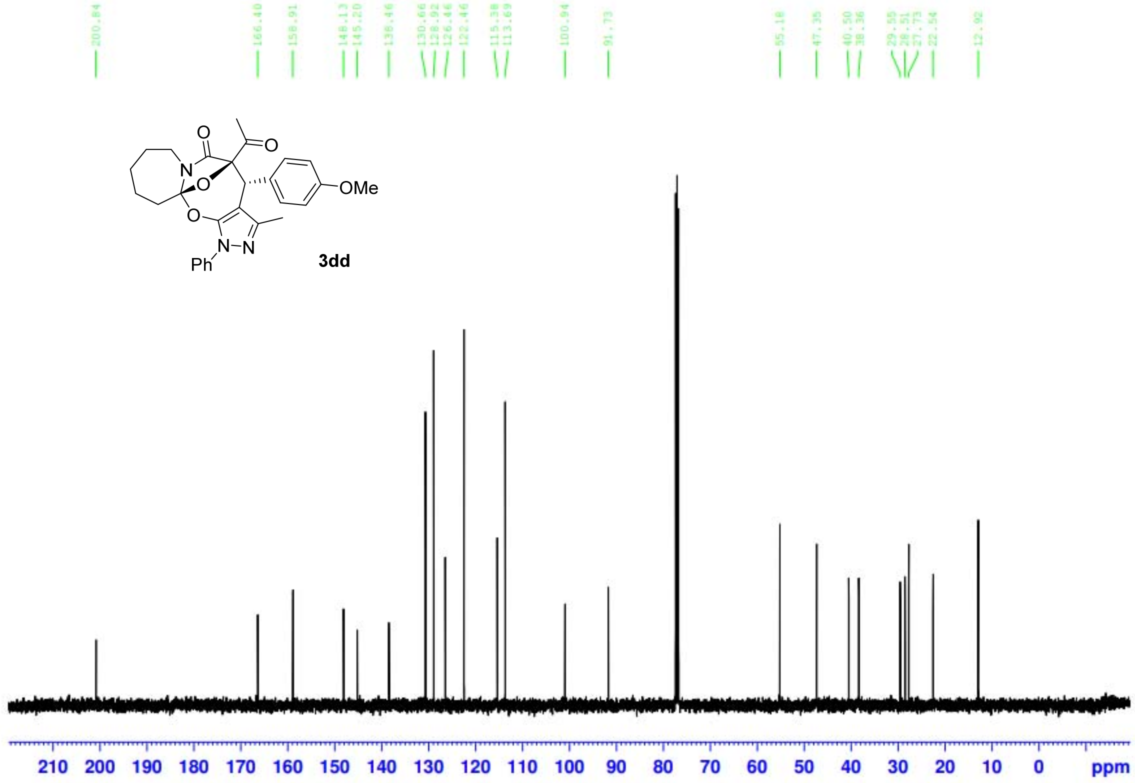

5-acetyl-3-methyl-4-(naphthalen-1-yl)-1-phenyl-4,5,9,10,11,12-hexahydro-8H-5,12a-epoxyazepino[2,1-b]pyrazolo[4,3-g][1,3]oxazocin-6(1H)-one (3de):

^{1}H NMR (400MHz,CDCl$_3$) of 3de

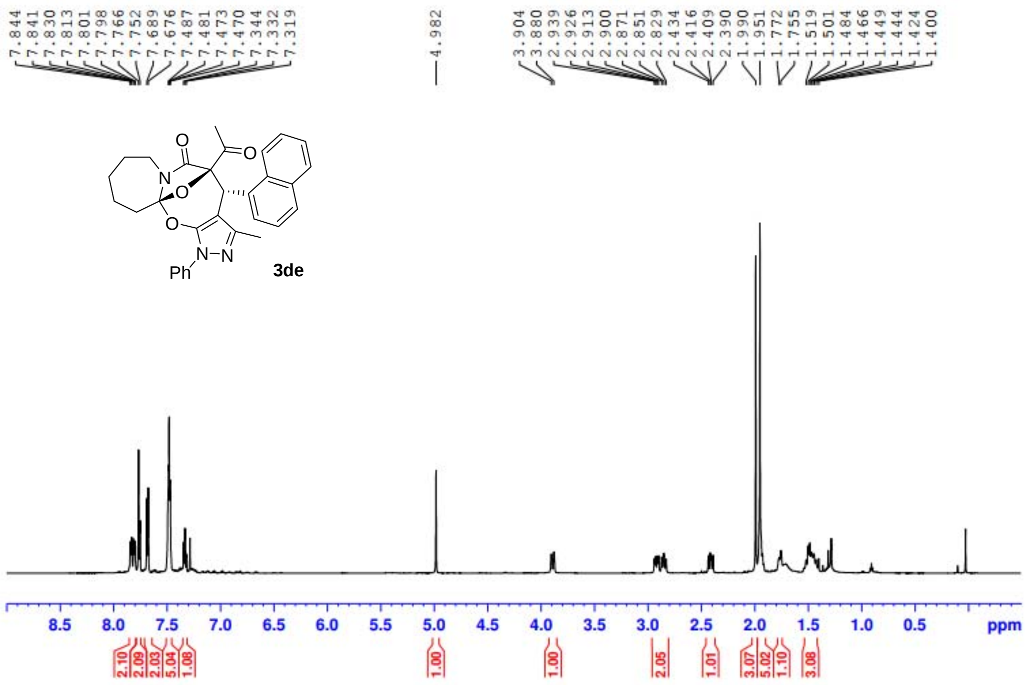

^{13}C NMR (100MHz,CDCl$_3$) of 3de

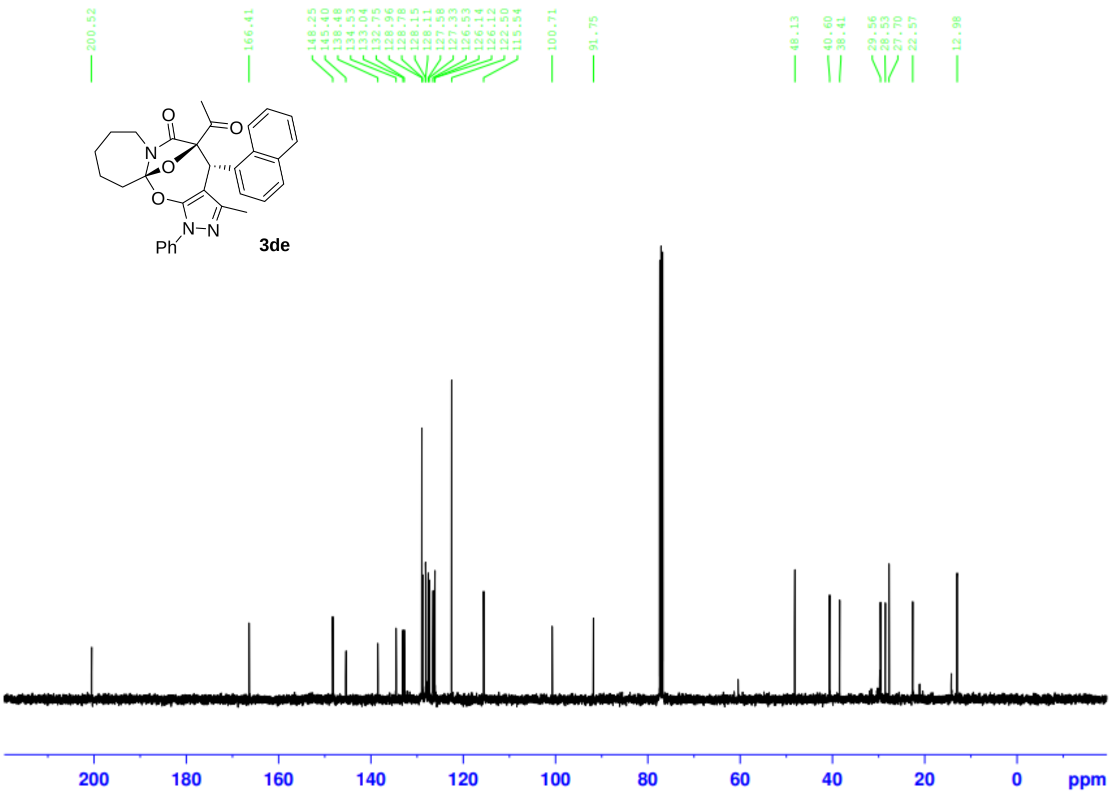

5-acetyl-3-methyl-4-phenyl-1-(p-tolyl)-4,5,9,10,11,12-hexahydro-8H-5,12a-epoxyazepino[2,1-b]pyrazolo[4,3-g][1,3]oxazocin-6(1H)-one (3dg):

^{1}H NMR (400MHz,CDCl$_3$) of 3dg

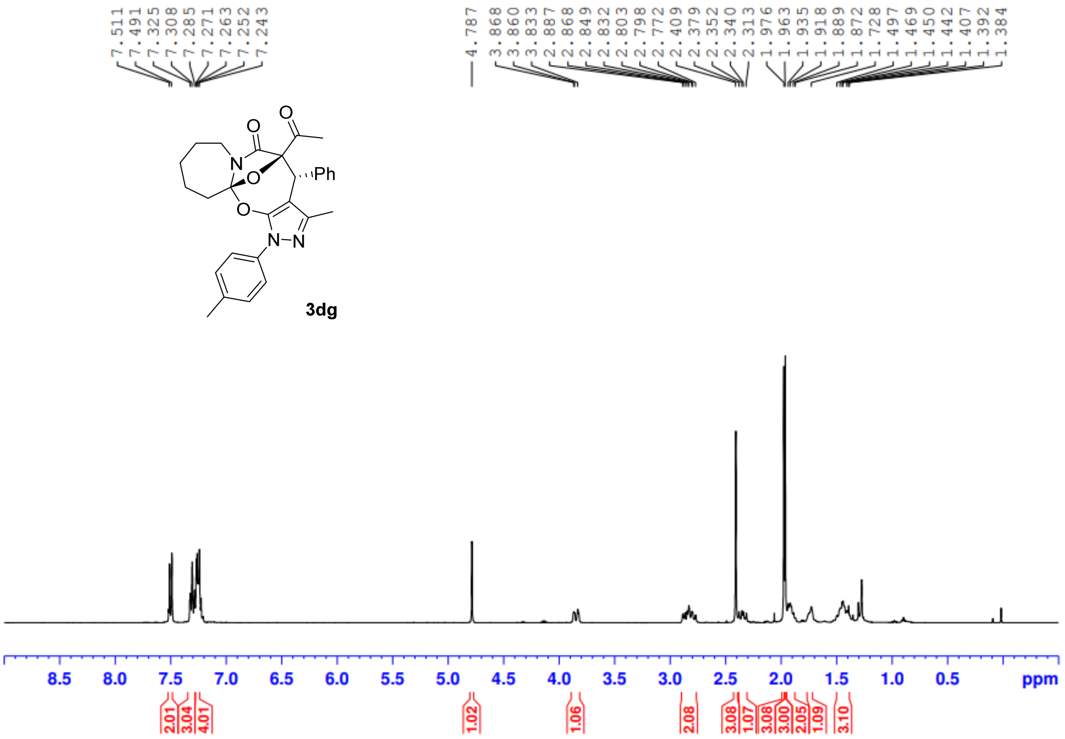

^{13}C NMR (100MHz,CDCl$_3$) of 3dg

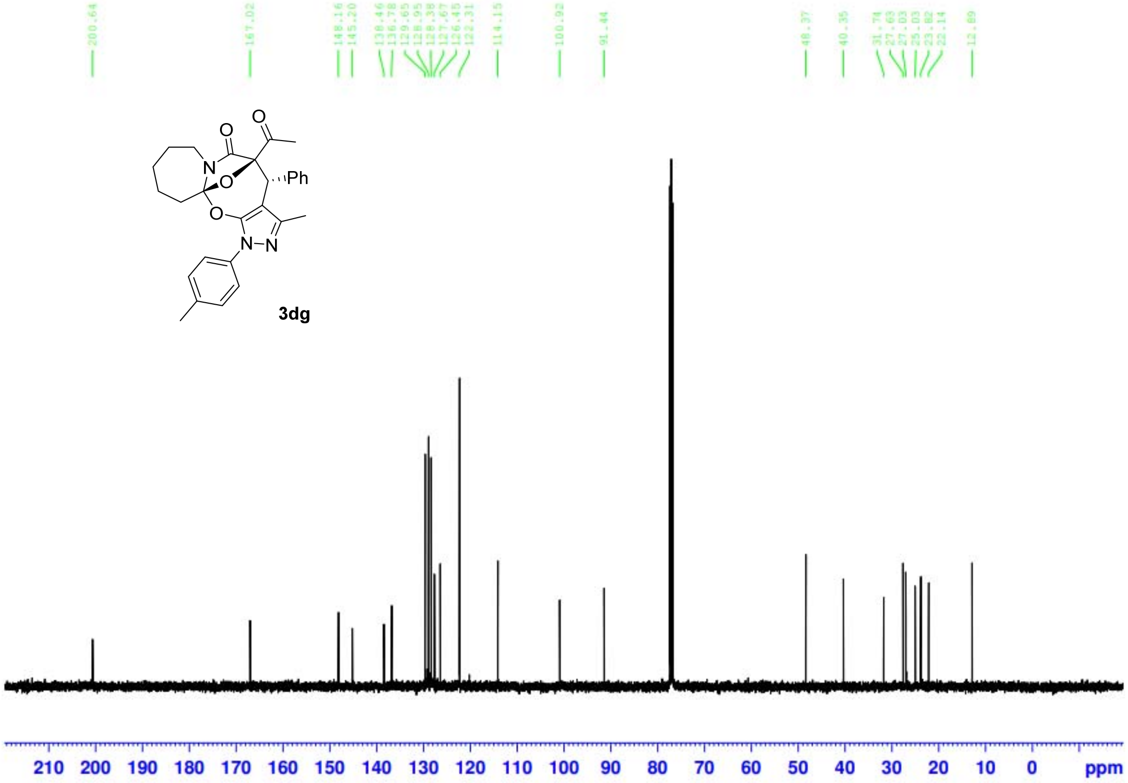

5-acetyl-3-methyl-1-phenyl-4-(3,4,5-trimethoxyphenyl)-4,5,9,10,11,12-hexahydro-8H-5,12a-epoxyazepino[2,1-b]pyrazolo[4,3-g][1,3]oxazocin-6(1H)-one (3dl):

^{1}H NMR (400MHz,$CDCl_3$) of 3dl

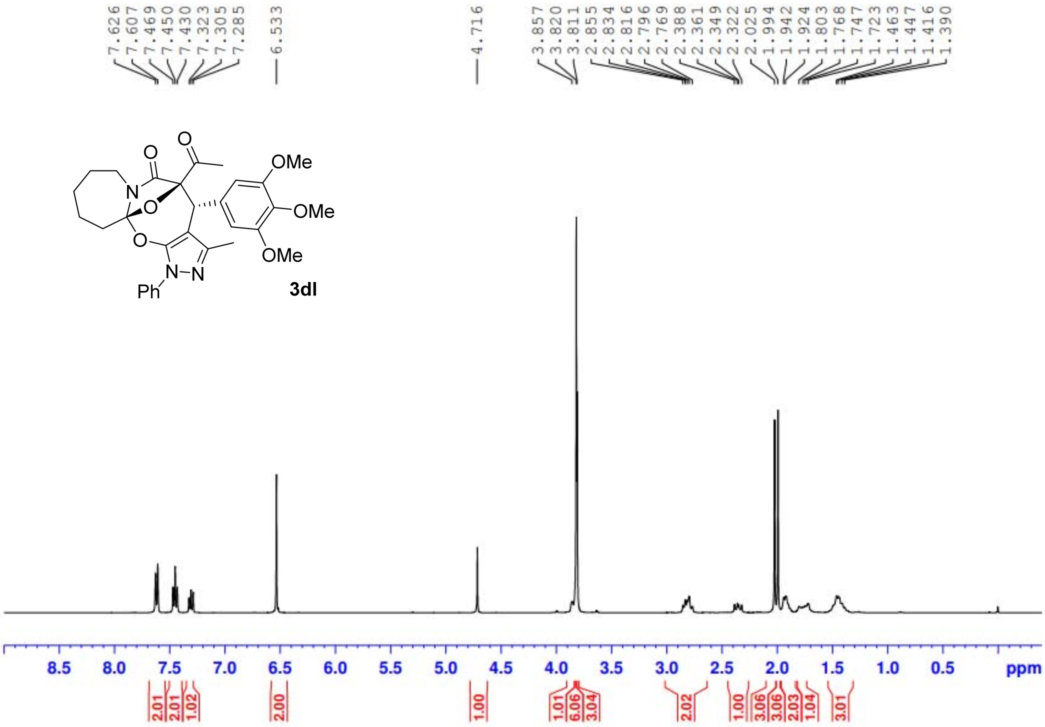

^{13}C NMR (100MHz,$CDCl_3$) of 3dl

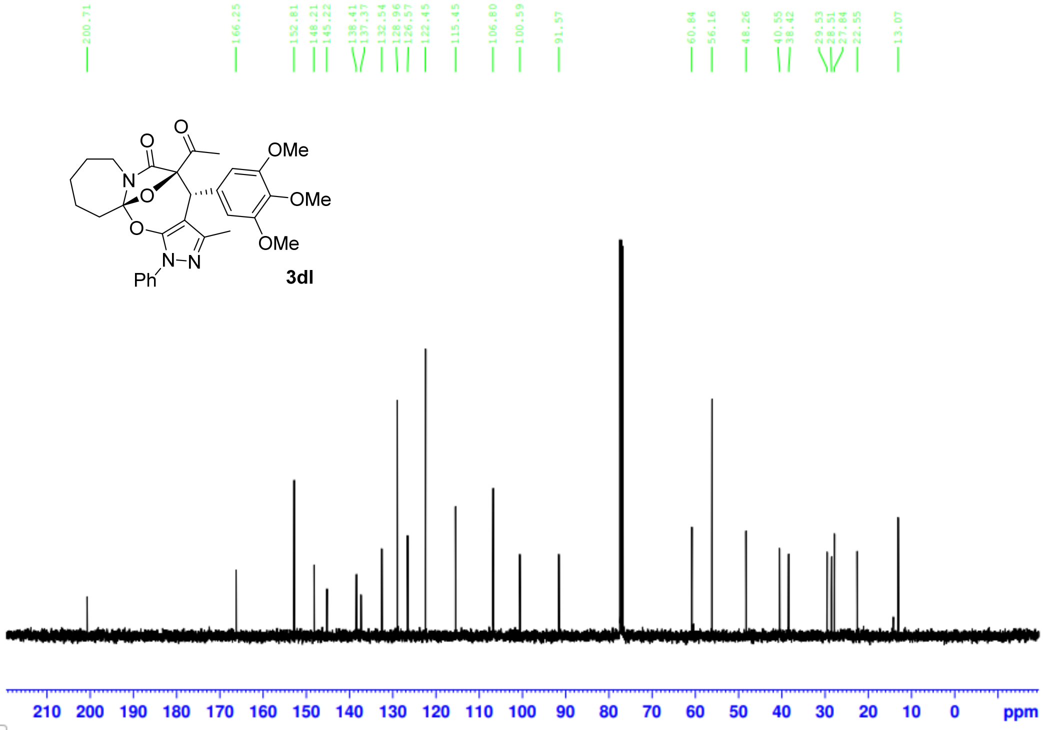

5-acetyl-4-(2-methoxyphenyl)-3-methyl-1-phenyl-4,5,9,10,11,12-hexahydro-8H-5,12a-epoxyazepino[2,1-b]pyrazolo[4,3-g][1,3]oxazocin-6(1H)-one (3dn):

^{1}H NMR (400MHz,$CDCl_3$) of 3dn

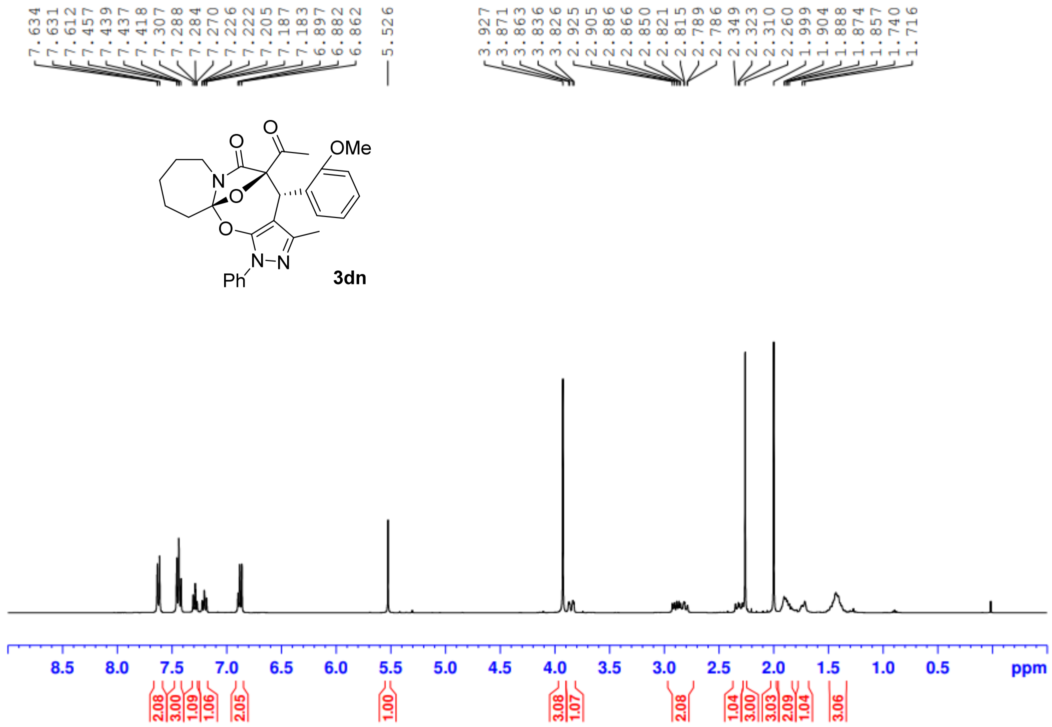

^{13}C NMR (100MHz,$CDCl_3$) of 3dn

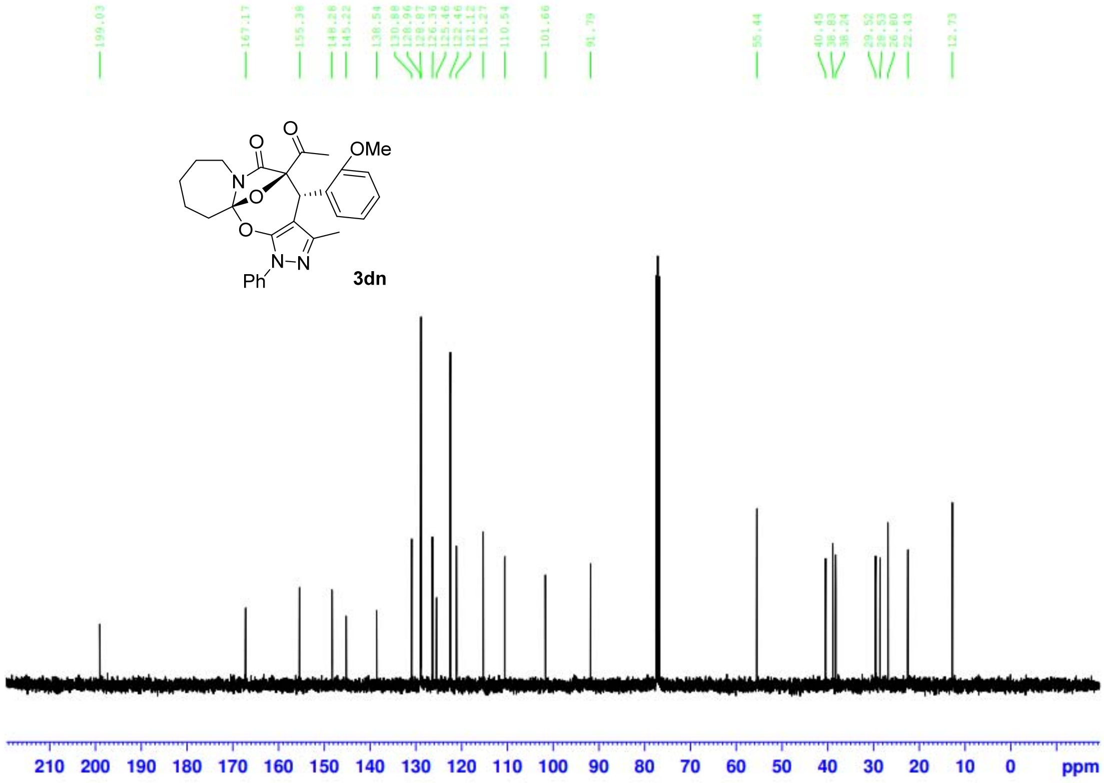

5-acetyl-4-(4-methoxyphenyl)-3-methyl-1-phenyl-4,5,8,9,10,11,12,13-octahydro-5,13a-epoxyazocino[2,1-b]pyrazolo[4,3-g][1,3]oxazocin-6(1H)-one (3ed):

^{1}H NMR (400MHz,CDCl$_3$) of 3ed

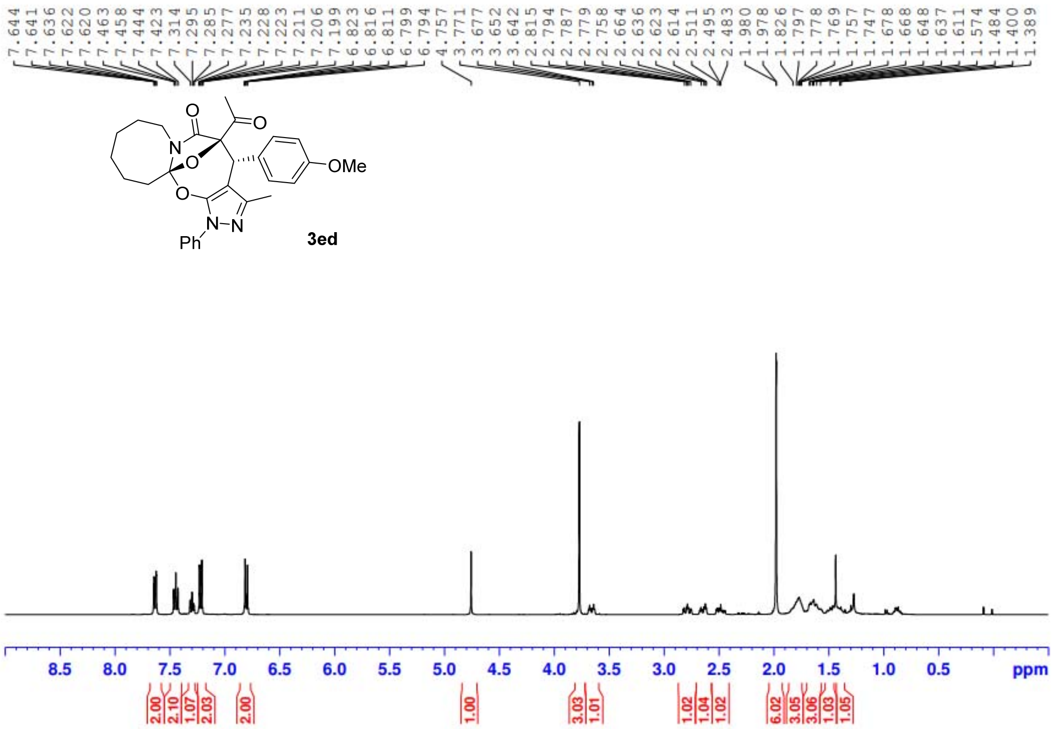

^{13}C NMR (100MHz,CDCl$_3$) of 3ed

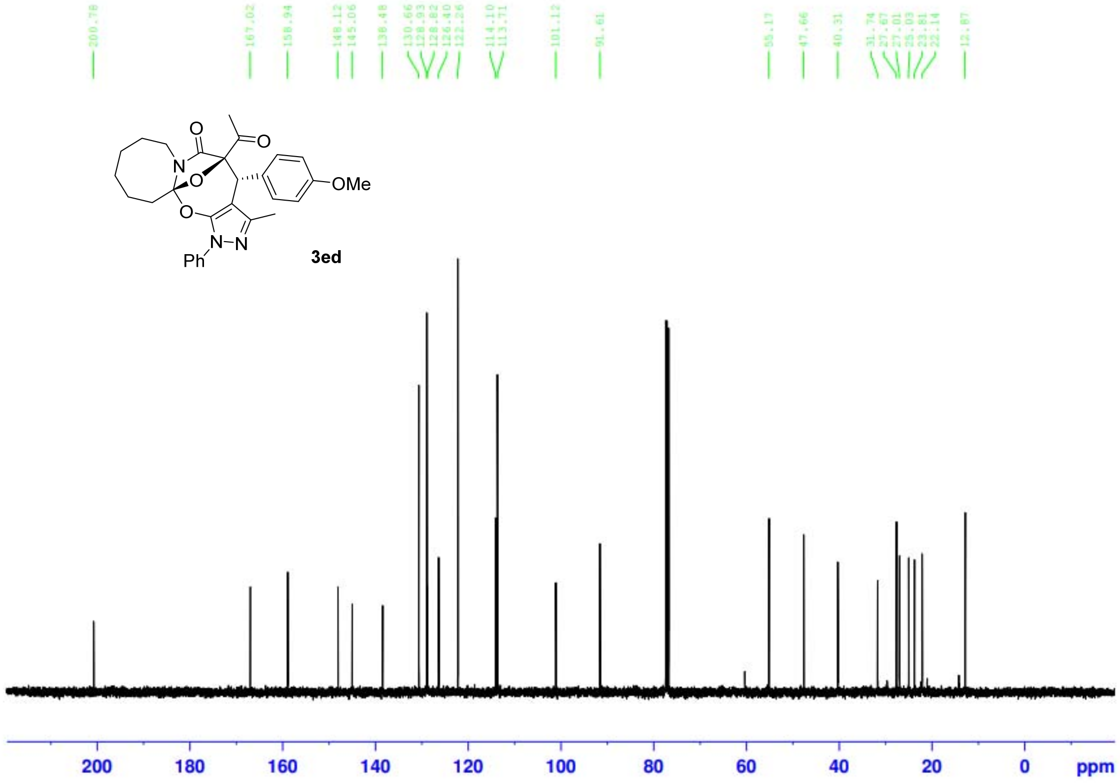

5-acetyl-3-methyl-1-phenyl-4-(3,4,5-trimethoxyphenyl)-4,5,8,9,10,11,12,13-octahydro-5,13a-epoxyazocino[2,1-b]pyrazolo[4,3-g][1,3]oxazocin-6(1H)-one (3el):

^{1}H NMR (400MHz,CDCl$_3$) of 3el

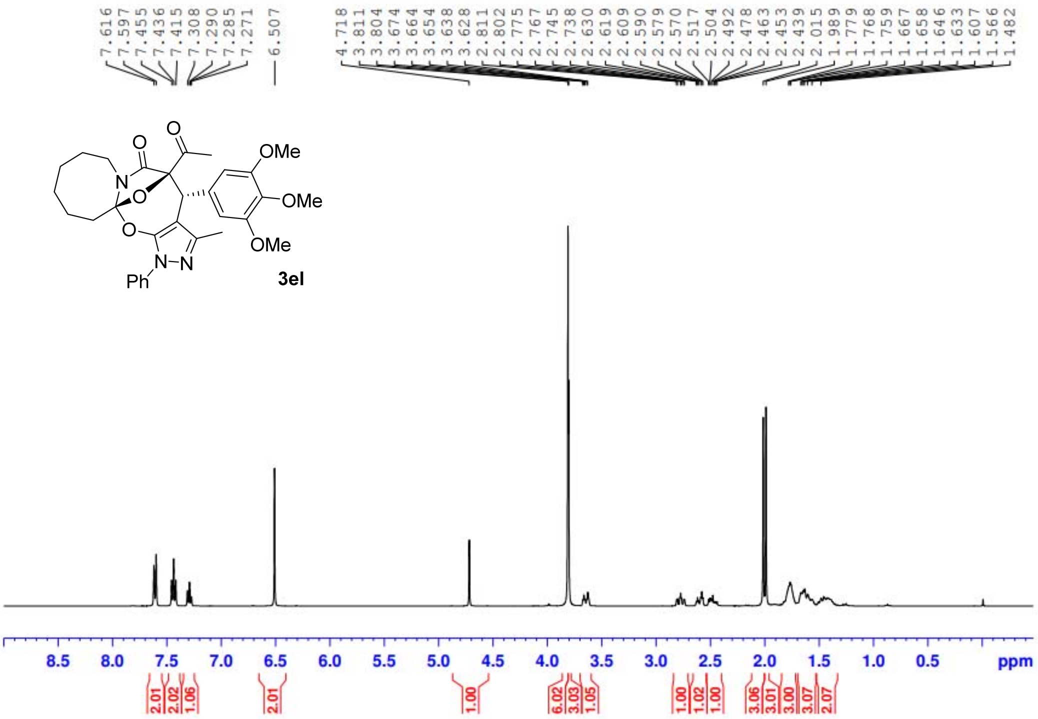

^{13}C NMR (100MHz,CDCl$_3$) of 3el

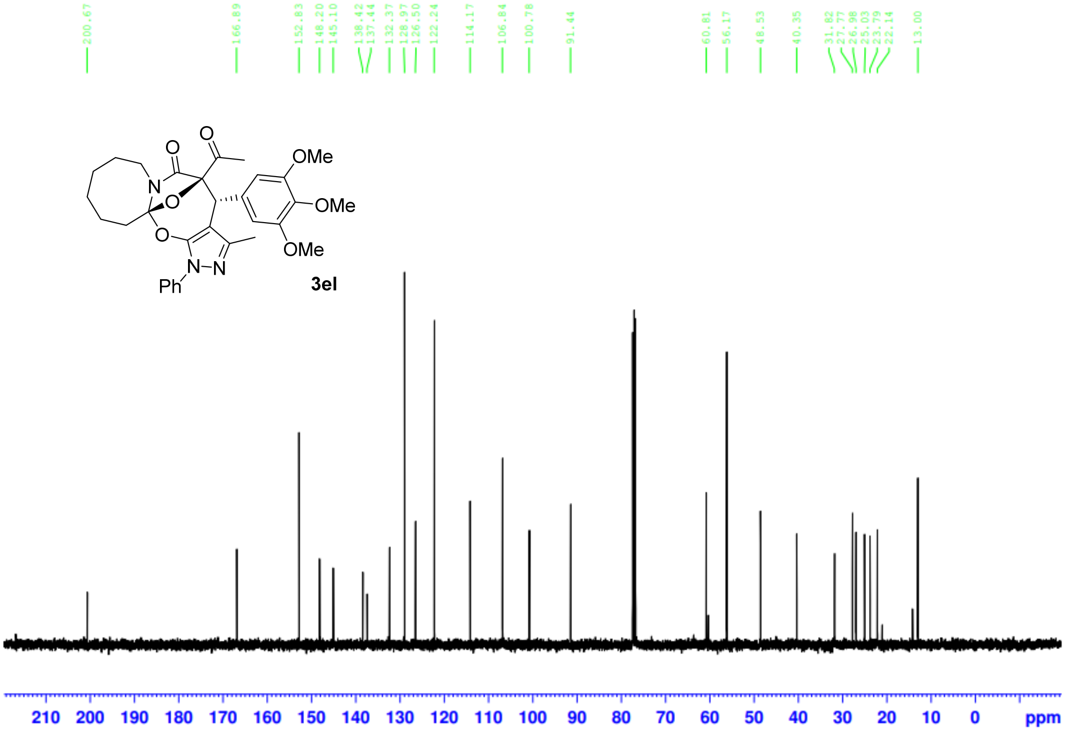

5-acetyl-4-(2-methoxyphenyl)-3-methyl-1-phenyl-4,5,8,9,10,11,12,13-octahydro-5,13a-epoxyazocino[2,1-b]pyrazolo[4,3-g][1,3]oxazocin-6(1H)-one (3en):

^{1}H NMR (400MHz,CDCl$_3$) of 3en

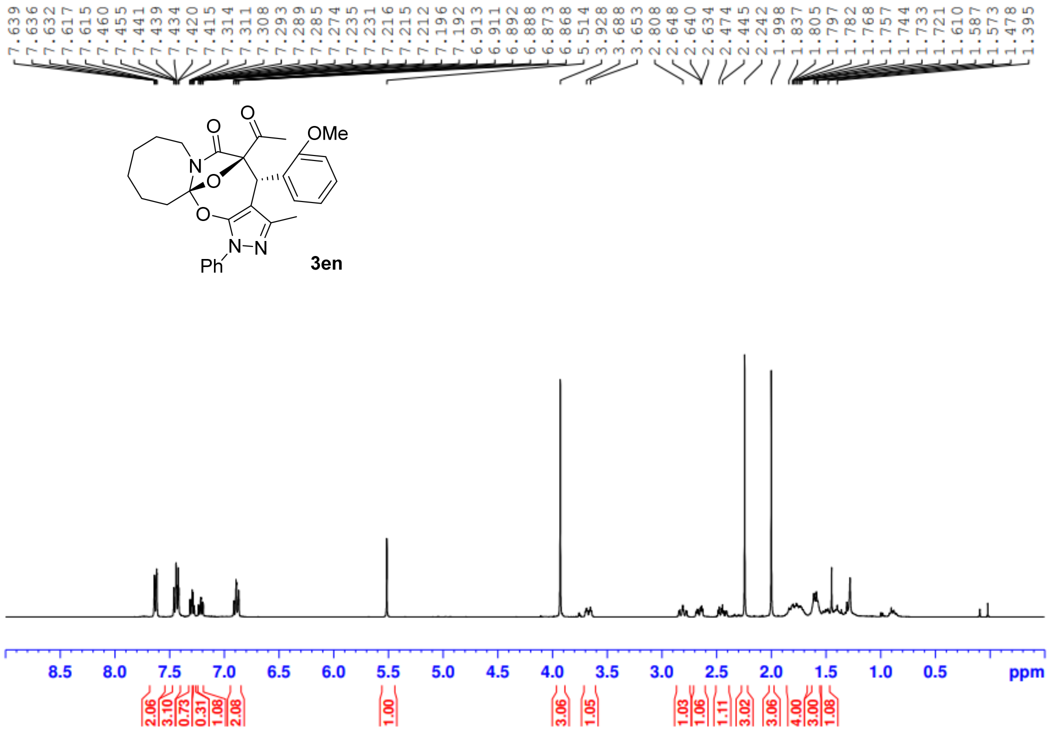

^{13}C NMR (100MHz,CDCl$_3$) of 3en

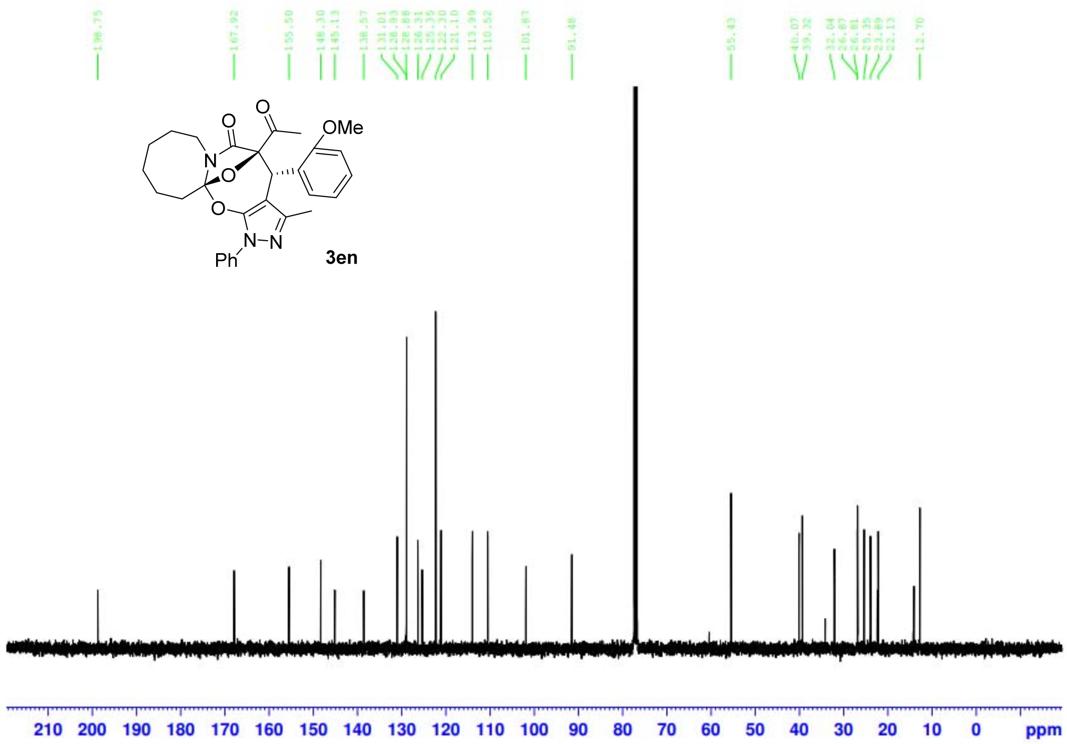

7-benzoyl-12-benzyl-2,3,7,12-tetrahydro-1H-6,13a-epoxypyrrolo[2',1':2,3][1,3]oxazocino[8,7-b]indol-5(6H)-one (3bq):

^{1}H NMR (400MHz,CDCl$_3$) of 3bq

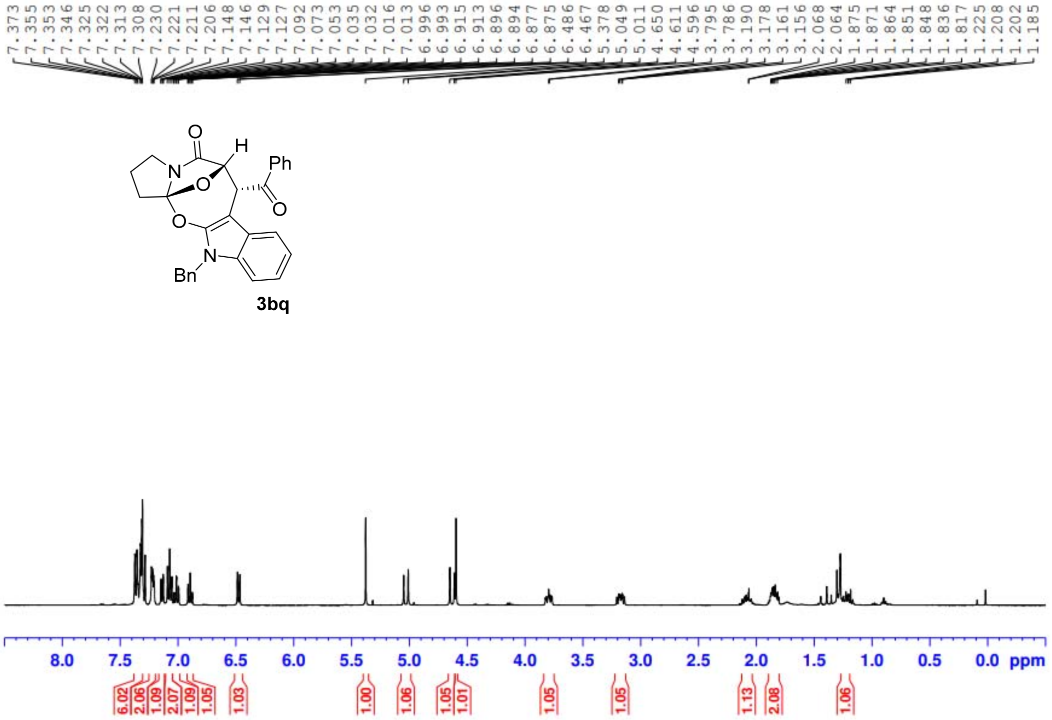

^{13}C NMR (100MHz,CDCl$_3$) of 3bq

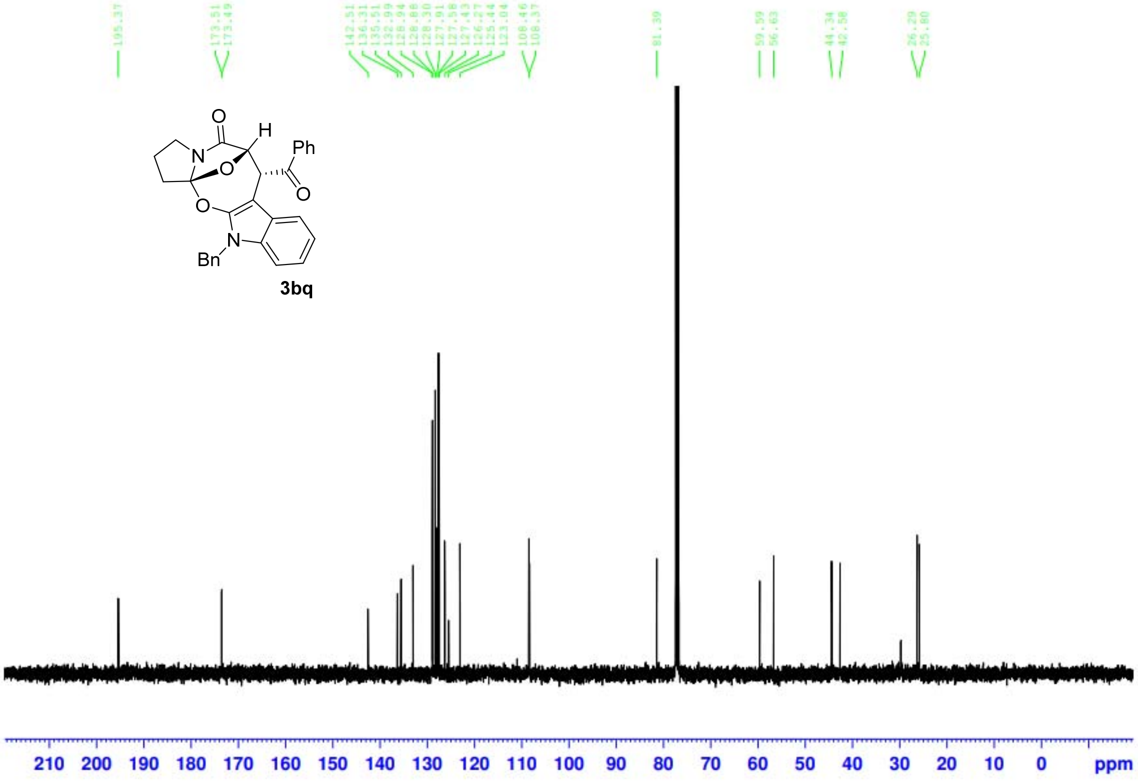

Dept-135 of 3bq

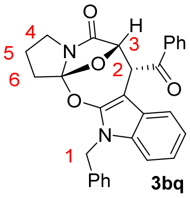

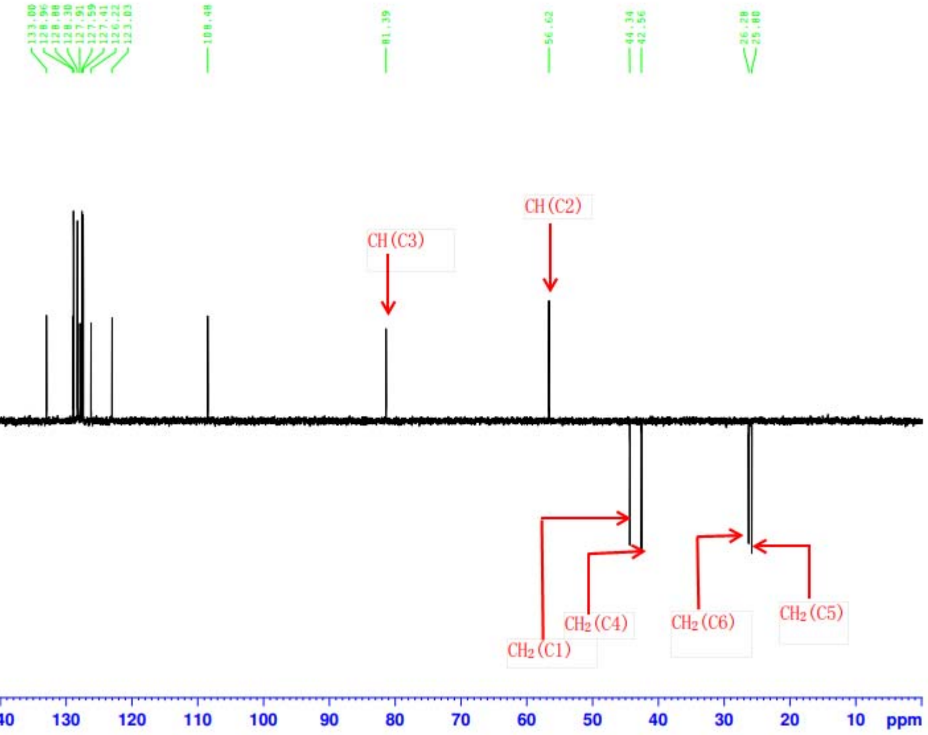

3-methyl-1,4-diphenyl-4,5,9,10-tetrahydro-8H-5,10a-epoxypyrazolo[4,3-g]pyrrolo[2,1-b][1,3] oxazocin-6(1H)-one (4):

^{1}H NMR (400MHz,CDCl$_3$) of 4

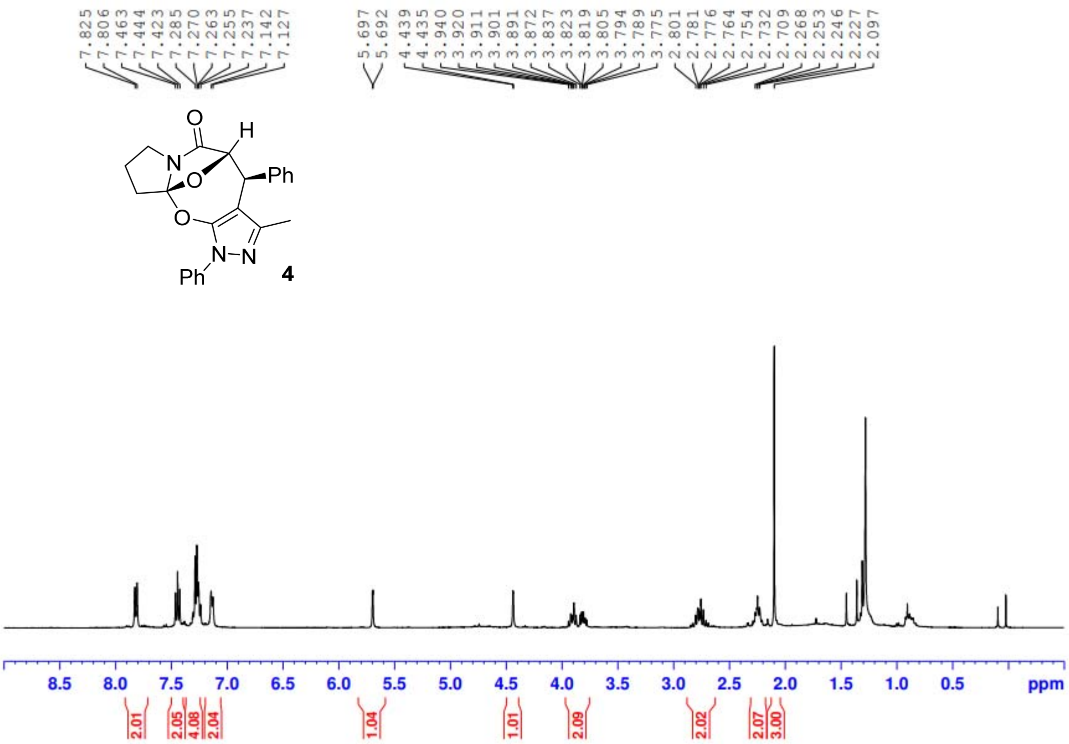

^{13}C NMR (100MHz,CDCl$_3$) of 4

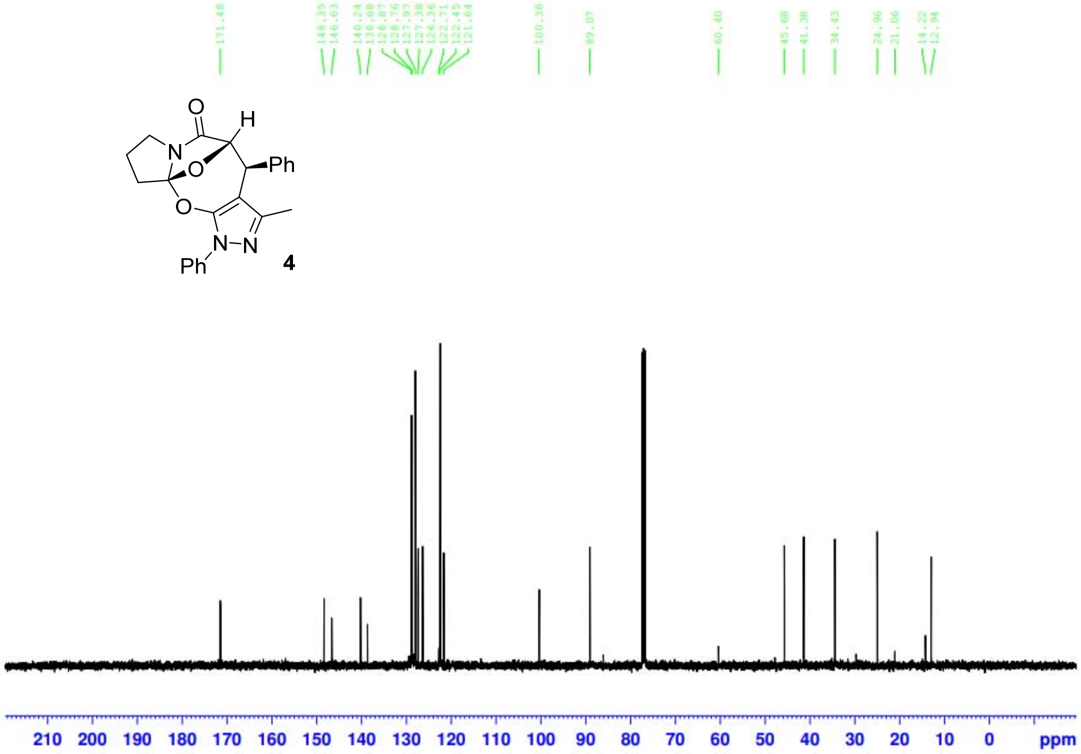

5-(1-hydroxyethyl)-3-methyl-1,4-diphenyl-4,5,9,10-tetrahydro-8H-5,10a-epoxypyrazolo[4,3-g]pyrrolo[2,1-b][1,3]oxazocin-6(1H)-one (5):

^{1}H NMR (400MHz,$CDCl_3$) of 5

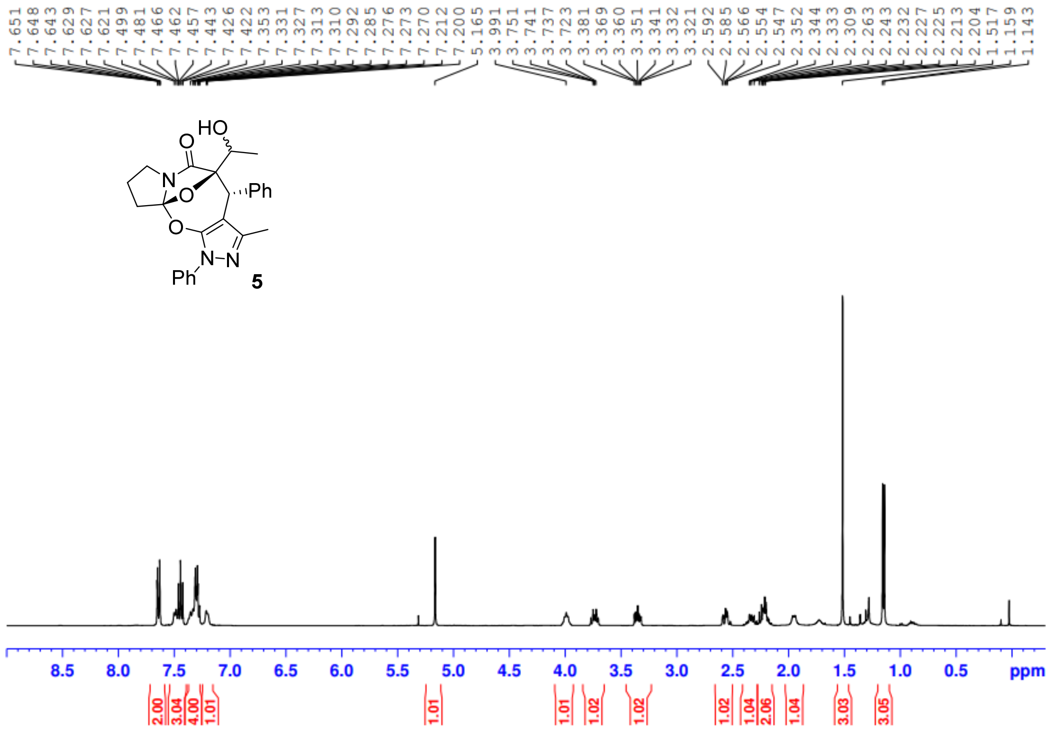

^{13}C NMR (100MHz,$CDCl_3$) of 5

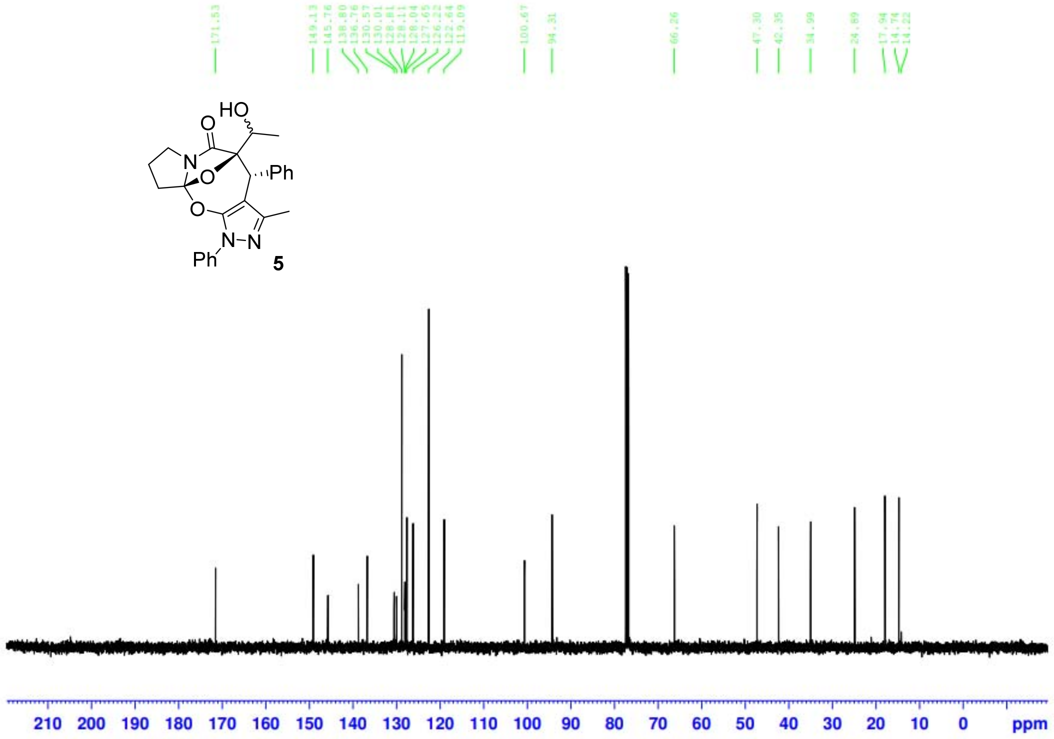

5-(2-hydroxybut-3-en-2-yl)-3-methyl-1,4-diphenyl-4,5,9,10-tetrahydro-8H-5,10a-epoxypyrazolo[4,3-g]pyrrolo[2,1-b][1,3]oxazocin-6(1H)-one (6):

^{1}H NMR (400MHz,CDCl$_3$) of 6

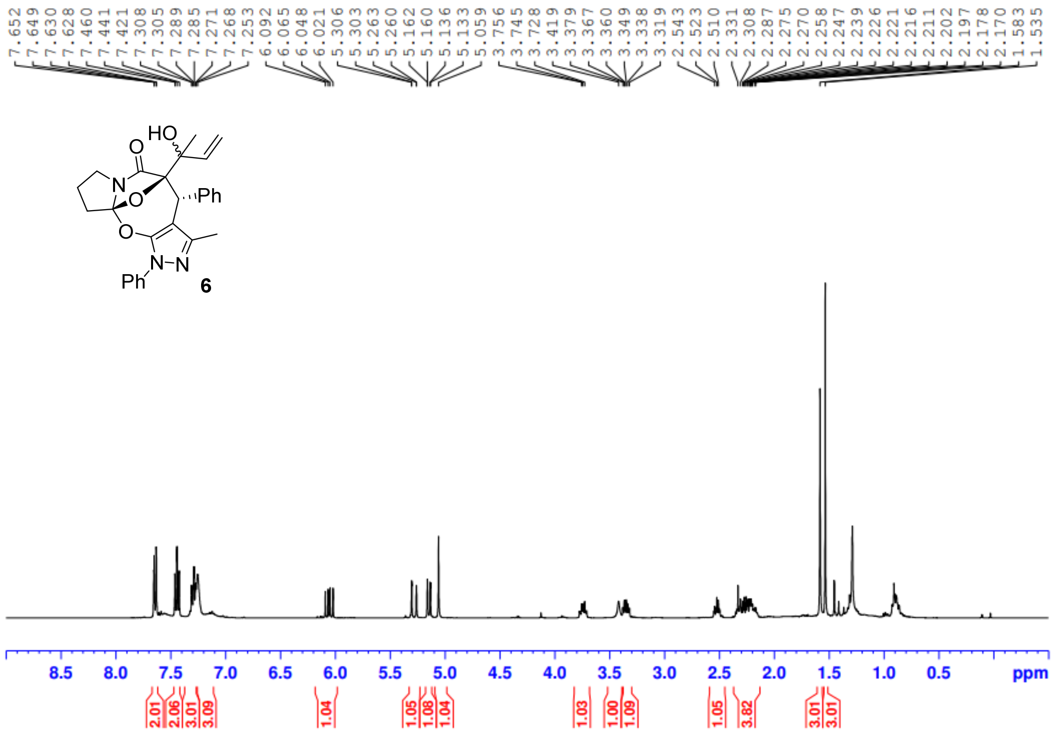

^{13}C NMR (100MHz,CDCl$_3$) of 6

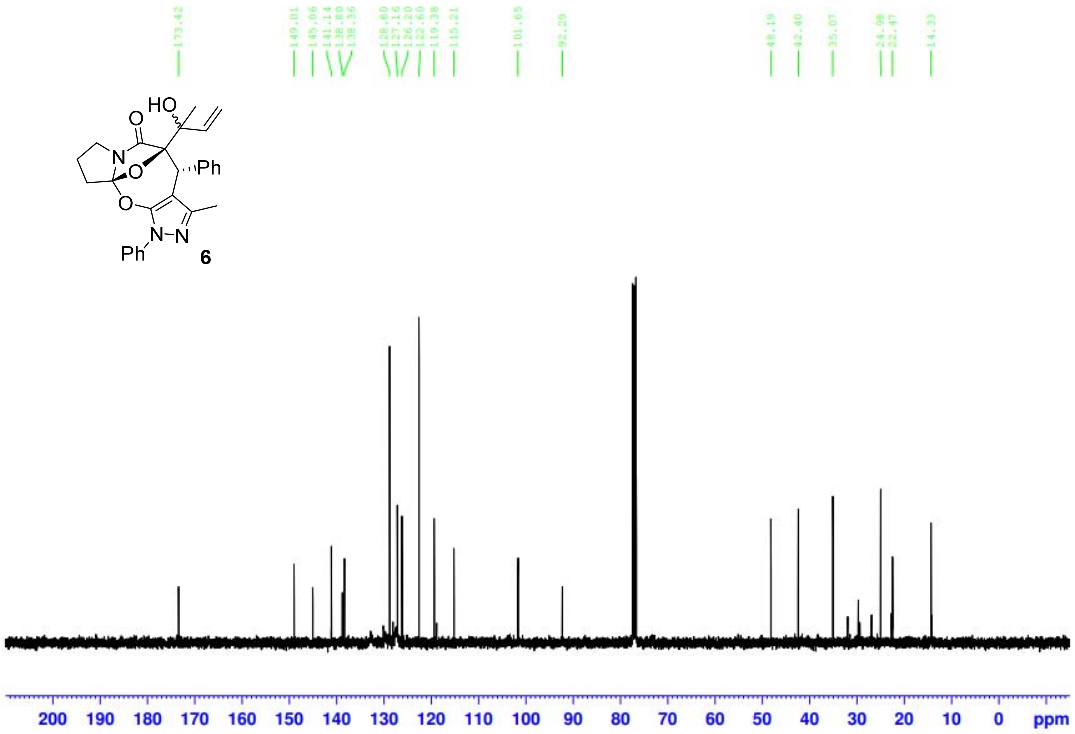

5-((E)-1-(methoxyimino)ethyl)-3-methyl-4-phenyl-1-(p-tolyl)-4,5,8,9,10,11-hexahydro-5,11a-epoxypyrazolo[4,3-g]pyrido[2,1-b][1,3]oxazocin-6(1H)-one (7):

^{1}H NMR (400MHz,$CDCl_3$) of 7

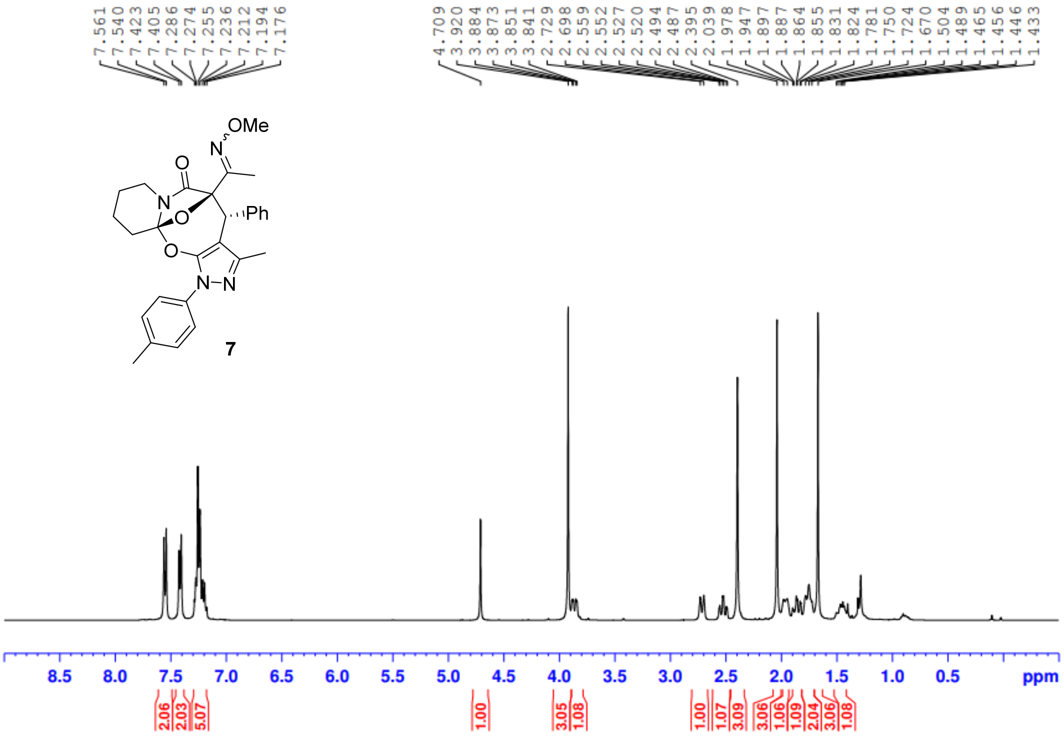

^{13}C NMR (100MHz,$CDCl_3$) of 7

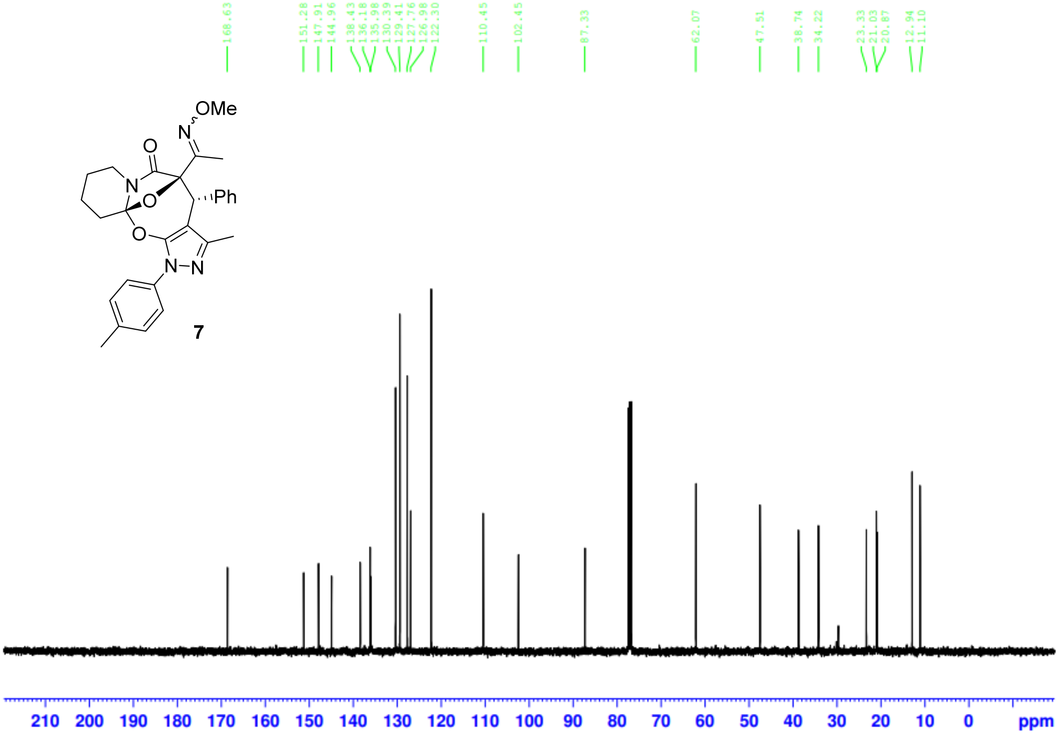

2. X-Ray Crystal Data of Compound 3aa and 4

Single crystal of **3aa** was obtained from the mixed solution of EtOH and petroleum ether via slow evaporation in a sample bottle. A suitable crystal was selected and its X-ray intensity data were measured at Rigaku-mm007 diffractometer with Mo Kα radiation. The crystal structure was solved by using the crysalispro and olex2 structure solution programs.

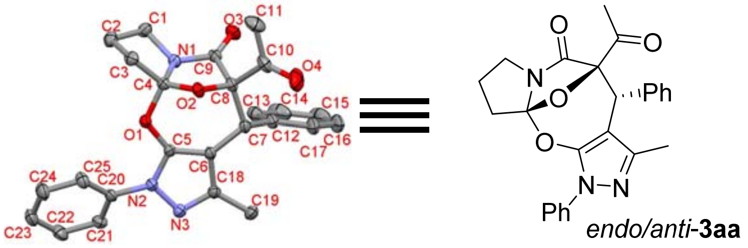

Figure S1. X-ray single crystal structure of **3aa** (with thermal ellipsoils shown at the 50% probability level)

Table S1. Crystal data and structure refinement for 3aa.

Identification code	**3aa**
Empirical formula	$C_{25}H_{23}N_3O_4$
Formula weight	429.46
Temperature/K	113.15
Crystal system	monoclinic
Space group	$P2_1/n$
a/Å	14.6107(6)
b/Å	9.0141(3)
c/Å	16.1963(6)
α /°	90
β /°	101.479(4)
γ /°	90
Volume/Å^3	2090.42(14)
Z	4
ρ calcg/cm^3	1.365
μ /mm^{-1}	0.094
F(000)	904.0
Crystal size/mm^3	0.24 × 0.22 × 0.18
Radiation	Mo Kα (λ = 0.71073)
2Θ range for data collection/°	4.194 to 65.782

Index ranges	$-21 \leqslant h \leqslant 18, -13 \leqslant k \leqslant 13, -24 \leqslant l \leqslant 24$
Reflections collected	25566
Independent reflections	7247 [R_{int} = 0.0640, R_{sigma} = 0.0532]
Data/restraints/parameters	7247/1/292
Goodness-of-fit on F^2	1.057
Final R indexes [I>=2σ (I)]	R_1 = 0.0581, wR_2 = 0.1407
Final R indexes [all data]	R_1 = 0.0809, wR_2 = 0.1609
Largest diff. peak/hole / e $Å^{-3}$	0.64/-0.47

Single crystal of **4** was obtained from the mixed solution of EtOH and petroleum ether via slow evaporation in a sample bottle. A suitable crystal was selected and its X-ray intensity data were measured at Rigaku-mm007 diffractometer with Mo Kα radiation. The crystal structure was solved by using the crysalispro and olex2 structure solution programs.

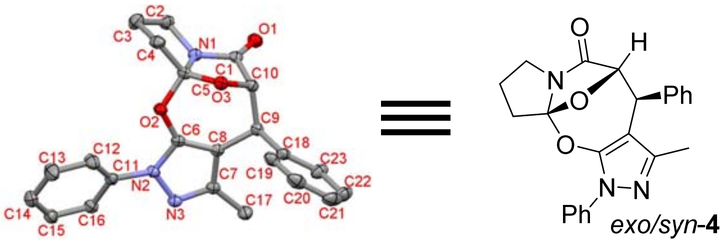

Figure S2. X-ray single crystal structure of **4** (with thermal ellipsoils shown at the 50% probability level)

Table S2. Crystal data and structure refinement for 3aa.

Identification code	**4**
Empirical formula	$C_{23}H_{21}N_3O_3$
Formula weight	387.43
Temperature/K	113.15
Crystal system	monoclinic
Space group	$P2_1/n$
a/Å	9.20310(10)
b/Å	17.82720(10)
c/Å	11.92480(10)
α /°	90
β /°	106.9210(10)
γ /°	90
Volume/Å^3	1871.75(3)
Z	4
ρ calcg/cm^3	1.375
μ /mm^{-1}	0.751
F(000)	816.0
Crystal size/mm^3	0.26 × 0.23 × 0.17
Radiation	Cu Kα (λ = 1.54184)
2Θ range for data collection/°	9.204 to 158.044
Index ranges	$-11 \leqslant h \leqslant 11, -22 \leqslant k \leqslant 22, -14 \leqslant l \leqslant 15$
Reflections collected	32598
Independent reflections	3966 [R_{int} = 0.0258, R_{sigma} = 0.0129]

Data/restraints/parameters	3966/0/264
Goodness-of-fit on F2	1.028
Final R indexes [I>=2σ (I)]	R_1 = 0.0362, wR_2 = 0.0923
Final R indexes [all data]	R_1 = 0.0366, wR_2 = 0.0926
Largest diff. peak/hole / e Å^{-3}	0.30/-0.20
